# Convergent Modifiers of Exposure Variability in Approved Cannabinoid Medicines: Pharmacogenomics, Drug–Drug Interactions, and Parent–Metabolite Divergence

**DOI:** 10.3390/pharmaceutics18101293

**Published:** 2026-10-12

**Authors:** Amina M. Bagher

**Affiliations:** Department of Pharmacology and Toxicology, Faculty of Pharmacy, King Abdulaziz University, Jeddah 21589, Saudi Arabia; abagher@kau.edu.sa; Tel.: +966-547187973

**Keywords:** cannabidiol, tetrahydrocannabinol, drug–drug interaction, pharmacogenomics, drug–drug–gene interaction, active metabolite, cytochrome P450

## Abstract

**Background/Objectives:** Systemic exposure to the approved cannabinoid medicines cannabidiol, nabiximols, dronabinol, and nabilone varies markedly among patients. Genotype and comedication may act at shared clearance steps and alter parent-drug and active-metabolite exposures differently. This review integrates pharmacogenomic, drug–drug interaction, and post-approval safety evidence within a convergent exposure-modifier (CEM) framework and develops testable hypotheses about their joint effects. **Methods:** A narrative, integrative review of bibliographic databases and regulatory documents for these medicines and key co-medications. Evidence was synthesized by pathway, product, analyte, interaction direction, directness, actionability, and limitations. **Results:** The framework distinguishes a victim branch, in which genotype and comedication modify cannabinoid exposure, from a perpetrator branch, in which cannabinoids alter co-medication exposure, with genotype potentially modifying it. Component evidence is strongest at CYP2C19 (cannabidiol–clobazam) and CYP2C9 (THC-containing medicines): cannabidiol increased N-desmethylclobazam exposure 3.4-fold in healthy volunteers while parent clobazam changed little, and CYP2C19 poor-metabolizer status independently increases baseline metabolite exposure. Comedication may phenoconvert genotype-predicted activity. No approved-product study identified in this review measured the joint genotype-by-comedication effect. Framework maturity is uneven: the predictions are cannabidiol-anchored; one relies on oral Δ^9^-THC evidence of unspecified formulation; nabiximols generates none, lacking genotype-stratified pharmacokinetics; and nabilone is excluded because its active-metabolite contributions are uncharacterized, although parent-drug activity is established. **Conclusions:** CEM is hypothesis-generating, not tested here: the three core predictions are falsifiable genotype-by-treatment tests requiring prospective validation. Label- and study-supported monitoring stands on its own evidence; the framework’s predictions do not, and genotype-guided dosing remains unvalidated.

## 1. Introduction

Four cannabinoid medicines hold regulatory approval in at least one major jurisdiction, and each is identified here by active ingredient, formulation, and product, since exposure depends on all three. Highly purified cannabidiol (CBD) oral solution (Epidiolex) is approved for Dravet syndrome, Lennox–Gastaut syndrome, and tuberous sclerosis complex [1]. Nabiximols (Sativex), a botanical oromucosal spray containing approximately equal Δ^9^-tetrahydrocannabinol (THC) and CBD, is approved for multiple sclerosis-related spasticity by national procedures in the United Kingdom and elsewhere, but not by the U.S. Food and Drug Administration (FDA) [2,3]. Dronabinol (synthetic Δ^9^-THC, Marinol) and nabilone (a synthetic THC analog, Cesamet), both oral capsules, are approved for chemotherapy-induced nausea and vomiting, and dronabinol is also approved for anorexia associated with acquired immunodeficiency syndrome [4,5,6].

None of the four was developed in populations stratified for pharmacogenomic variation, and use extends beyond the registration populations: routine-care cohorts show substantial CBD-exposure variability [7], older adults experience neuropsychiatric and fall-related effects [8], and polypharmacy with multimorbidity drives drug–drug–gene interactions [9]. Post-approval experience is therefore a principal source of incremental interaction and safety information [10,11].

The clinically salient common thread is variability in pharmacologically relevant systemic exposure, conditioned by food, formulation, route, hepatic function, inflammation, age, dose, and adherence, several of which may exceed the magnitude of the genotype [12,13,14]. Genotype and concurrent medication are examined here because they may affect shared metabolic or transport pathways and can be characterized when designing prospective studies of their joint effects.

**Figure 1:**
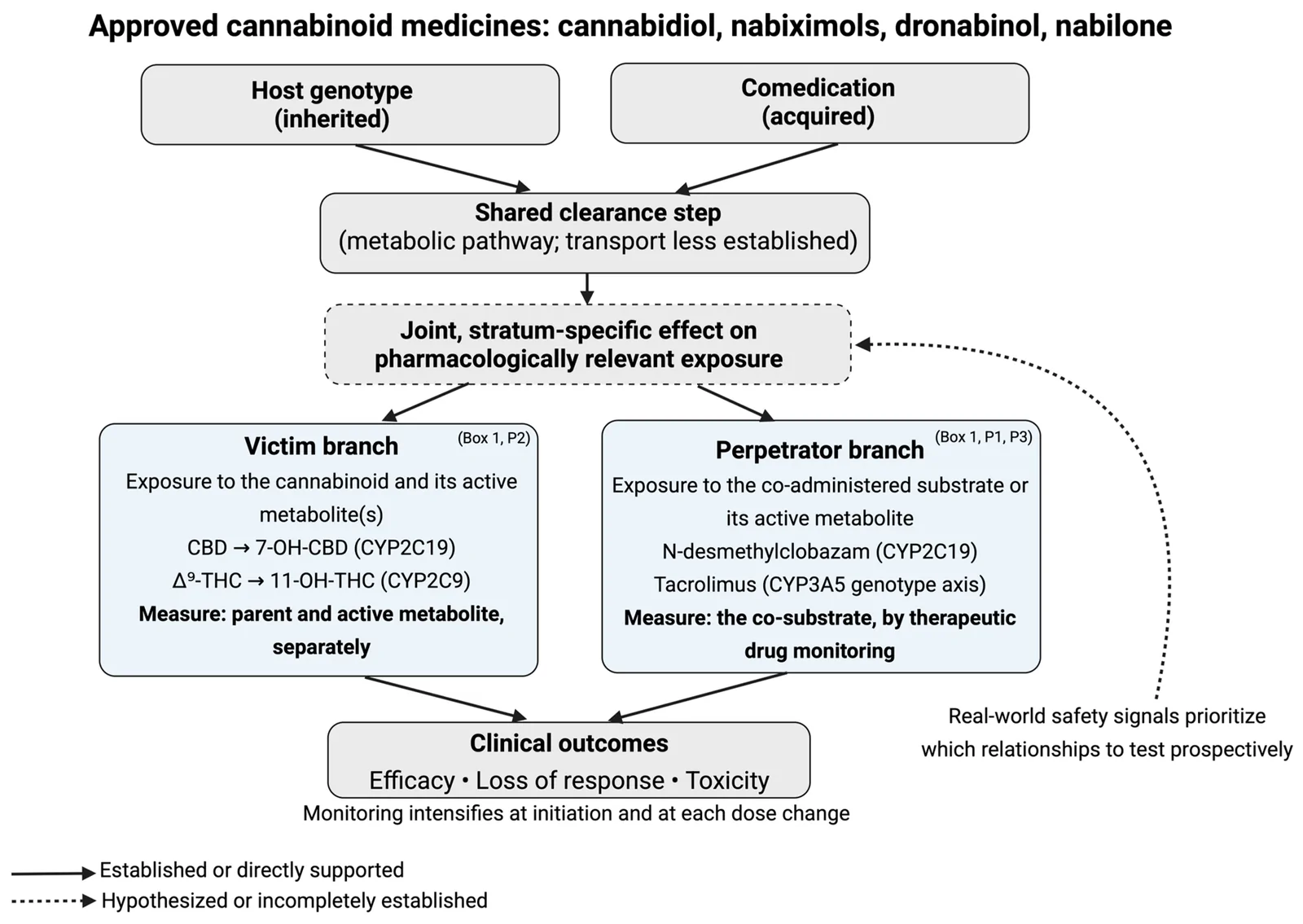
**The convergent exposure-modifier (CEM) framework.** Host genotype and comedication may act at the same clearance step; the resulting joint, stratum-specific effect on exposure is shown with a dashed outline because no approved-product study identified in this review has measured it (Box 1); P1 to P3 identify the corresponding predictions in that box. The component evidence supporting hypotheses of convergence is strongest at metabolic steps; transporter-mediated convergence is exploratory (Section 5.5). The two perpetrator examples shown have an established genotype axis. In both branches, the enzyme named identifies that genotype axis; it is not necessarily the only isoform contributing to the step, nor necessarily the isoform mediating the interaction. The arrows denote metabolic steps, not measured genotype effects: for Δ^9^-THC, the CYP2C9 genotype effect is established on parent exposure, whereas the effect on 11-OH-THC is not (Section 5.1; Figure 2B). Substrates with quantified evidence of interaction but no genotype axis, such as everolimus and mycophenolic acid, appear in the pathway and monitoring matrix (Section 7.2). The timing note follows the labeled monitoring schedule. Real-world safety signals prioritize which relationships to test prospectively; they do not measure exposure, and the resulting ordering is author-generated (Section 4.4). Nabilone appears in the banner of approved products but is excluded from the framework’s parent-plus-active-metabolite outputs, as its active metabolites are uncharacterized (Section 5.4). Contextual modifiers—food, formulation, organ function, age, and dose—also alter exposure but are not part of the convergence and are omitted here (Figure 2). Solid arrows, established or directly supported; dashed arrows, hypothesized or incompletely established; evidence sources in Table 1. The evidence-source markers of Figure 2 are not used here. CBD, cannabidiol; CYP, cytochrome P450; THC, Δ^9^-tetrahydrocannabinol; 7-OH-CBD, 7-hydroxy-cannabidiol; 11-OH-THC, 11-hydroxy-THC. Conceptual and monitoring aid, not a dosing algorithm. Created in BioRender. Bagher, A. (2026) https://BioRender.com/2aiahho.

These two modifiers and the exposure they act on define the convergent exposure-modifier (CEM) framework, a hypothesis-generating conceptual framework that requires prospective validation. It has two directions: a victim branch, in which genotype and comedication modify exposure to the cannabinoid and its active metabolites, and a perpetrator branch, in which the cannabinoid modifies exposure to a co-administered drug or its active metabolite, with genetically variable pathways potentially modifying that interaction. Post-approval safety signals are used here to prioritize which combinations merit prospective study; that prioritization is author-generated, reflecting an interpretation of the available pharmacovigilance, pharmacokinetic, and mechanistic evidence rather than a ranking the pharmacovigilance analysis establishes (Section 4.4). Figure 1 summarizes the framework and its two directions.

Drug–drug–gene interaction and phenoconversion are established concepts outside cannabinoid pharmacology, where such interactions have been formally classified [15], quantified in clinical populations [9], argued to warrant routine consideration [15,16], and incorporated into enzyme-specific dose-adaptation networks [17]. Within cannabinoid pharmacology, pharmacogenomic, interaction, and post-approval safety studies have largely been treated separately [11,18,19,20]; recent reviews have differed in emphasis: cataloging pharmacokinetic interactions [21], integrating CBD metabolism with antiseizure drug interactions in epilepsy [22], including phenoconversion as one determinant among several [23], or organizing evidence enzyme by enzyme across cannabis products more broadly [24]. An analogous integration in antiseizure pharmacology combines therapeutic drug monitoring, cytochrome P450 (CYP) 3A4/5 interactions, and CYP3A5*3 genotype within one dosing approach [25]. The present review builds on these contributions by organizing evidence for approved cannabinoid medicines around separate parent-drug and active-metabolite exposures and proposing candidate genotype-by-treatment hypotheses.

**Figure 2:**
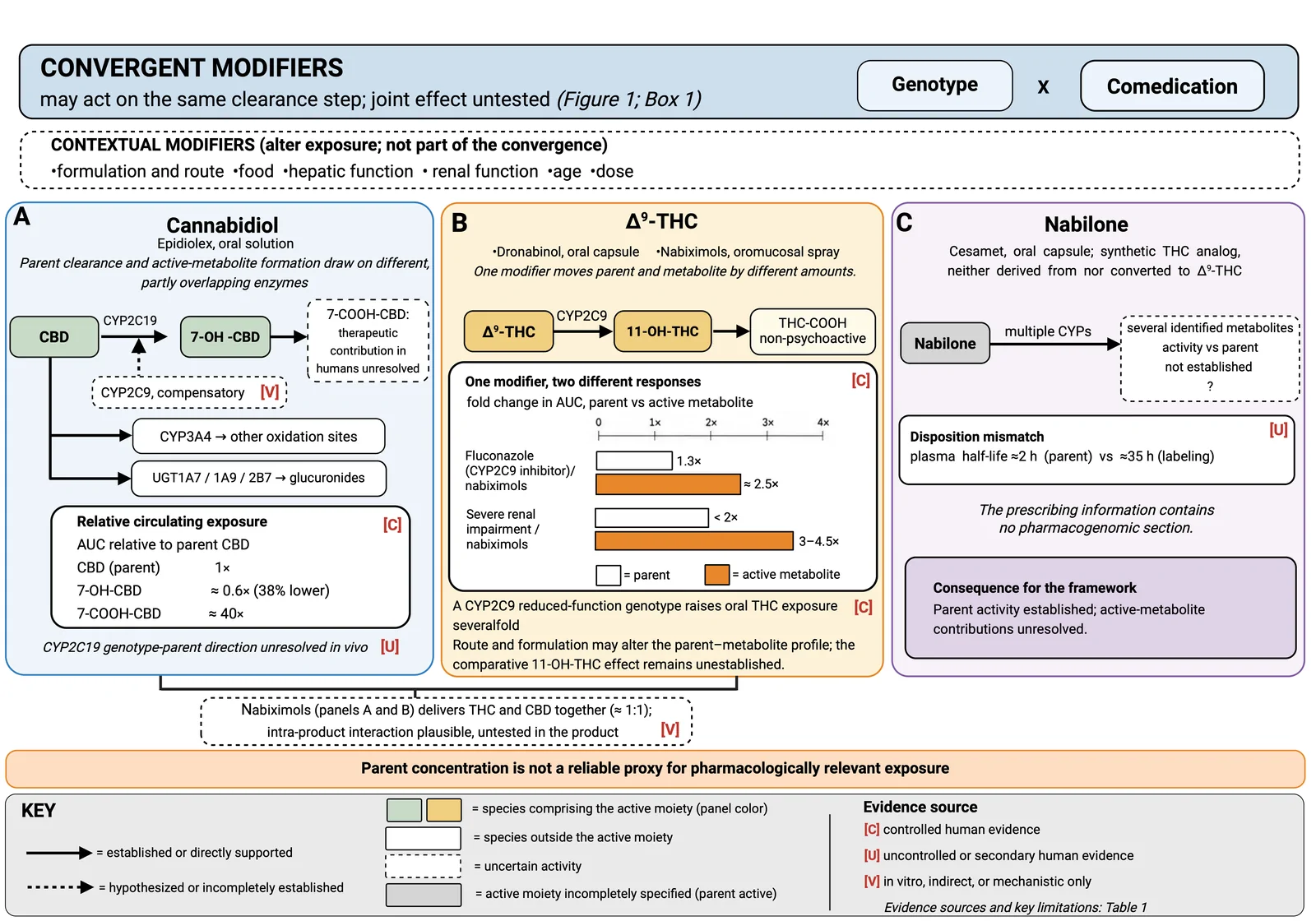
**Metabolic routes of the approved cannabinoid medicines and the modifiers acting on them.** Convergent modifiers (genotype, comedication) may act jointly at a single clearance step (Figure 1; Box 1); contextual modifiers alter exposure but are not part of that convergence. (**A**) **Cannabidiol.** The compensatory CYP2C9 contribution to 7-hydroxylation rests on in vitro evidence only (Section 5.2; Box 1). 7-Carboxy-cannabidiol (7-COOH-CBD) is the major circulating metabolite; its activity at therapeutic targets is unsettled, although it inhibits BCRP- and BSEP-mediated transport at clinically relevant concentrations, and its exclusion from the pharmacologically active species is provisional. The in vivo direction of the CYP2C19–parent relationship is paradoxical and is discussed in the text. (**B**) **Δ^9^-THC.** 11-Nor-9-carboxy-THC (THC-COOH) is non-psychoactive and is not among the pharmacologically active species but is retained as a disposition marker for the same CYP2C9-mediated step. Fluconazole and severe renal impairment each affect parent and metabolite exposures to different degrees; both were measured in nabiximols, an oromucosal product co-formulated with CBD, and should not be extrapolated to an oral capsule. The CYP2C9 genotype effect shown is on parent Δ^9^-THC exposure; parent and carboxy metabolite moved in opposite directions, and the effect on 11-OH-THC itself is not established. Route and formulation may alter the parent–metabolite profile; the comparative 11-OH-THC effect between oral dronabinol and oromucosal nabiximols remains unestablished (Section 3.2). (**C**) **Nabilone**. Parent-drug activity is established; the identities and contributions of its potentially active metabolites are not, so a parent-plus-active-metabolite profile cannot be specified, and nabilone is absent from the pathway matrix and the predictions (Section 7.2). Solid arrows, established or directly supported; dashed arrows, hypothesized or incompletely established. Shaded boxes, pharmacologically active species (parent with its active metabolite); open boxes, species outside it; dashed boxes marked “?”, uncertain activity; gray fill, used for nabilone alone: parent activity established, active-metabolite contributions unresolved. Bracketed markers apply to annotations rather than arrows, and in the panel B inset the bar fills identify the species compared: [C], controlled human evidence, meaning a prospective human study carrying a designed comparison, whether between groups or within subjects and whether or not blinded; a single-arm disposition or case series carrying no such comparison is marked [U]. The tier is assigned by the design of the underlying study, not by regulatory status: a labeled statement is marked [C] only where the study it reports is controlled, and is otherwise marked by that study’s own design, the label being the source of the report rather than a tier of evidence; the design behind each annotation is given in Table 1; [U], uncontrolled or secondary human evidence; [V], in vitro, indirect, or mechanistic only. Evidence sources and key limitations: Table 1. AUC, area under the concentration–time curve; CBD, cannabidiol; CYP, cytochrome P450; THC, Δ^9^-tetrahydrocannabinol; UGT, UDP-glucuronosyltransferase; 7-OH-CBD, 7-hydroxy-cannabidiol; 11-OH-THC, 11-hydroxy-THC. Conceptual and research aid, not a dosing algorithm. Created in BioRender. Bagher, A. (2026) https://BioRender.com/ph9umsy.

The review focuses on these four approved medicines. Evidence from other cannabis-derived products or formulations is considered only where relevant to their pharmacology, with limitations in product-specific applicability stated explicitly. In particular, the CYP2C9 genotype evidence for oral Δ^9^-THC of unspecified formulation is not treated as direct evidence for the approved dronabinol capsule. The evidence base is dominated by CBD, and the gaps for the other three differ in kind: dronabinol carries the only quantified label-level pharmacogenomic signal, at CYP2C9, with limited product-specific interaction data; nabiximols has direct controlled-interaction evidence but no genotype-stratified pharmacokinetics; and nabilone, although active as the parent compound, has metabolites whose contribution to its effects is not established. This review synthesizes pharmacogenomic, drug–drug interaction, and post-approval safety evidence for these medicines within a single exposure logic and proposes the prospective genotype-by-treatment studies through which the framework’s central proposition could be tested. No eligible approved-product study directly testing the proposed genotype-by-comedication effects was identified in the literature reviewed.

## 2. Methods

This is a narrative, integrative review. Its purpose is to synthesize evidence across three historically separate studies—cannabinoid pharmacogenomics, drug–drug interactions, and post-approval safety—and to develop hypotheses from that synthesis. It is not designed or reported as a systematic review [26]; thus, no claim of exhaustive retrieval is made. The convergent exposure-modifier framework set out below is a hypothesis-generating conceptual structure; nothing in this review validates it. Evidence from unregulated products, non-approved extracts, or recreational exposure was included only where relevant to an approved medicine and was identified as indirect. Off-label use of an approved product was classified by product provenance, with indication, population, and dose considered separately when assessing applicability. Search documentation and a structured appraisal of the pivotal studies are provided as Supplementary Tables S1 and S2.

PubMed/MEDLINE, Scopus, Web of Science, and Google Scholar were searched for literature published up to 13 August 2026. That date is the coverage end date of the review; it is not a record of the dates on which individual searches were run, which were not retained. Search terms combined the four approved medicines with pharmacokinetics, pharmacogenomics, drug–drug interactions, pharmacovigilance, and precision dosing; the representative string is reproduced in Supplementary Table S1. The database-specific adaptations of that string, and the numbers of records retrieved, screened, and excluded, were not retained and are therefore not reported; no study-flow diagram can be constructed for the original search. Adaptations of the representative string to the syntax of each indexed database are nevertheless given in Supplementary Table S1.2, so that a reader can run an equivalent search; they are reproduction aids written for that purpose and not a record of the strings executed. The eligibility and characterization logic applied to each evidence stream is set out in Supplementary Figure S1, which is a schema of that logic and not a study-flow diagram. Google Scholar was used as a supplementary check for records not retrieved by the three indexed databases rather than as a primary source; its queries, the number of records screened, and any stopping rule were not recorded, and no stopping rule was prespecified. Included records are not attributed by retrieval source, so the number of records that entered the review through Google Scholar alone cannot be stated. Retrieval was confined to English-language publications. The restriction is an operational limit of a single-reader review: no translation or non-English full-text verification was undertaken. It is not a judgment that the relevant evidence for these products is published in English, and it carries the attendant risk of language bias (Section 9.2). Citation chaining was bidirectional, and forward citation tracking was applied to the studies underpinning the load-bearing claims. Targeted update searches were repeated before the original submission for each relationship on which a negative claim rests; these relationships are listed in Supplementary Table S1, and the negative claims made in this review are bounded by these searches. A separate confirmatory search of those same relationships was run at the revision stage, on 1 October 2026, and is reported in Supplementary Table S1.5 with the six query strings, the record counts, and the outcome for each relationship. Its protocol was fixed before any record was screened, and it is reported apart from the original search, which it does not replace; the six approved-product evidence-gap statements were retained, and indirect evidence prompted qualification of two broader statements, in Section 5.5 and Section 5.6.

Eligibility was defined by evidence type before screening. Pharmacogenomic studies required genotype- or phenotype-stratified pharmacokinetic or clinical outcomes for an approved cannabinoid or a cannabinoid-relevant enzyme substrate; clinical pharmacokinetic and controlled interaction studies required a quantified change in human systemic exposure to an approved cannabinoid, its metabolites, or a co-administered substrate; and pharmacovigilance studies required disproportionality estimates from a named spontaneous-reporting database using a specified method. Regulatory sources, listed with their revision dates in Supplementary Table S1, were rechecked against the live agency or manufacturer record before submission. In vitro, preclinical, and animal evidence was included where it established pathway assignment or characterized metabolite pharmacology, but was not used to estimate the magnitude of clinical effects.

Searching, screening, full-text selection, and evidence characterization were performed by one author, without independent duplicate assessment. The review was undertaken as single-author work; no second reviewer was involved at any stage, so independent duplicate screening and characterization were not available. Titles and abstracts were screened against the criteria above, and full texts retrieved where a record met them or its eligibility was unclear from the abstract. Where several publications reported the same dataset or cohort, the most complete or most recent was used and the others cited only for analytic detail; such overlap was identified from author, center, and trial identifiers and is recorded in Table 1 footnotes and in Supplementary Table S2. No data were pooled. Single-reader screening and characterization carry a potential for selection and interpretive bias that cannot be quantified here. No formal appraisal instrument was applied at any of the three relevant levels: study-level risk of bias, review-level methodological assessment, or certainty of evidence across studies. This is a limitation of the review; it does not follow from the heterogeneity of the evidence or from the absence of meta-analysis. In place of a validated instrument, a structured descriptive critical appraisal of the studies underpinning the main quantitative claims and the framework hypotheses is provided in Supplementary Table S2, reporting design, population, dosing conditions, precision, replication, dataset overlap, and the inference each study supports. Comparability across the evidence streams is handled by constraint rather than by grading: the streams are not treated as interchangeable, and each is limited in what it may support. In vitro, preclinical, and animal studies establish pathway assignment and metabolite pharmacology but are not used to estimate the magnitude of clinical effects; regulatory documents supply labeling statements and recommended actions but do not stand above the studies they summarize; uncontrolled observations, whether single case reports or disproportionality estimates, are identified as such under evidence source and design, and the constraints on any value they contribute are recorded under key limitations and uncertainty; and the basis for clinical action is reported as a separate field, so that no action is attributed to evidence that does not support it. The manuscript drew informally on the domains of the Scale for the Assessment of Narrative Review Articles [27] to guide reporting; no scored self-assessment or external appraisal was obtained.

Table 1 characterizes each load-bearing relationship across four reported fields: the evidence source and design; its directness to the approved product; the basis, if any, for clinical action; and the key limitations and uncertainty attached to it. The basis for clinical action is reported in four actionability categories: label-supported action, where the product information states the action; controlled-PK-supported monitoring, where a dedicated human study supports it but no labeling does; author-generated vigilance, where neither does, and the suggestion is the author’s inference, a hypothesis-generating, research-oriented consideration rather than a validated clinical management recommendation; and research only or no action indicated, where no clinical action follows. Where two apply to one scenario, both are given. Direct evidence derives from the approved product itself, though not necessarily at labeled doses or in the labeled population; partially direct evidence derives from the approved active ingredient or a closely related clinical formulation; indirect evidence derives from unregulated or non-approved preparations. Directness describes the provenance of evidence and is independent of its methodological quality; the two are reported separately. Dose and population bear on the applicability of a finding rather than on its directness: a high dose of an approved product limits applicability to labeled dosing without thereby making the evidence indirect. A labeled statement and the study on which it rests are treated as one source rather than as independent replication. Regulatory documents are used here as primary sources for a product’s labeling statements and recommended actions, not as a tier of evidence standing above the studies they summarize. Their inclusion does not constitute independent replication of the underlying clinical or nonclinical evidence; when a publication and a label report the same dataset, they are treated as linked reports, and evidential weight derives from the design of the underlying study rather than its regulatory status. Where that study is identifiable, it is appraised on its own terms in Table 1 and Supplementary Table S2; where it is not, or where access to its methods and results is limited, that limitation is recorded in the corresponding row. No summary confidence grade is assigned, and no row of Table 1 should be read as carrying a certainty rating. Evidence from unregulated products, high-dose extracts, or general cannabis exposure is admitted only as indirect evidence, mechanistic or clinical, and is identified as such at the point of use. This review is reported as a narrative integrative synthesis and makes no claim of compliance with PRISMA or any other systematic-review reporting standard: no protocol was registered, no study-flow count can be given for the original search (Supplementary Figure S1), screening and characterization were single-reader, and no validated risk-of-bias or certainty instruments were applied. Supplementary Table S2 states which appraisal domains its columns cover and that it does not substitute for such an instrument. These are constraints on what the review can support, and each is stated at the point where it bears on a claim.

**Table 1:** Consolidated evidence maps for the convergent exposure-modifier (CEM) framework: load-bearing exposure relationships in approved cannabinoid medicines. *Research and interpretive aid; not a validated dosing algorithm*. (**A**) Component exposure relationships, with evidence source, directness, actionability, and key limitations. Rows range from controlled approved-product evidence to exploratory and indirect findings; placement in this table does not denote established status. (**B**) Untested convergence hypotheses.

Exposure Relationship	CEM Branch	Evidence Source (Directness) (a)	Actionability	Key Limitations and Uncertainty (e)	Representative Sources
(**A**) Component exposure relationships					
Reduced-function CYP2C9 → higher oral THC/dronabinol exposure	Victim	Small oral-THC pharmacogenomic study (partially direct); dronabinol labeling (direct)	Label-supported action	Single unreplicated study; 43 healthy volunteers selected to enrich variant genotypes (four CYP2C9*3/*3 versus nineteen CYP2C9*1/*1); single 15 mg dose of oral Δ^9^-THC of unspecified formulation; applicability to the approved dronabinol capsule is unconfirmed.	[28,29]
CBD → higher N-desmethylclobazam (CYP2C19 inhibition)	Perpetrator	Phase 1 controlled PK study in healthy volunteers; uncontrolled cohort in children under expanded access; double-blind, placebo-controlled trial in patients on stable clobazam; regulatory labeling (direct)	Label-supported action	The phase 1 trial and the labeling derived from it are one source, not independent replication; the clobazam arm comprised twelve healthy volunteers. The pediatric evidence is an uncontrolled cohort in which clobazam doses were reduced in most participants, which truncates the observable rise; reported magnitude therefore differs between the two designs. The phase 2, double-blind, placebo-controlled trial provides an independent estimate with formal confidence intervals; 20 patients were randomized (16 cannabidiol, 4 placebo) and the pharmacokinetic analysis sets comprised 10 and 3, respectively; its headline ratio is a within-group day 33 to day 1 comparison, with the placebo arm showing no effect on either analyte.	[5,30,31,32]
CBD → higher tacrolimus exposure (C_max_ ≈ 4.2×, AUC ≈ 3.1×) (c)	Perpetrator	Interim analysis of a controlled PK trial in healthy volunteers; a single uncontrolled case report during pharmaceutical CBD treatment at 20–25 mg/kg/day (reported as 2000–2900 mg/day); regulatory labeling of both products (direct)	Label-supported action	Prespecified interim analysis, 12 completers, single center; shares a parent trial with the CYP2C19/CYP3A row (footnote c) and is not independent of it. The case report describes one patient, with no cannabidiol concentration measurements and no dechallenge assessment. The labeling statements are not independent of the studies on which they rest.	[33,34,35]
CBD → higher everolimus exposure (≈2.5×), half-life not prolonged	Perpetrator	Phase 1 controlled PK study in healthy volunteers; a single case report; regulatory labeling (direct)	Label-supported action	One phase 1 trial under healthy-participant dosing conditions, supported by a single case report rather than by independent controlled replication.	[36,37]
CBD → higher mycophenolic acid exposure (UGT1A9; AUC +35%)	Perpetrator	Human probe-substrate study; regulatory labeling (direct)	Label-supported action	Single probe-substrate study in healthy participants; probe exposure, not the clinical co-medication setting.	[5]
CBD → higher zidovudine exposure (UGT2B7; C_max_ +7%, AUC +19%)	Perpetrator	Human probe-substrate study; regulatory labeling (direct)	Label-supported: no action indicated	Single probe-substrate study; effect magnitude small and of uncertain clinical relevance.	[5]
Strong CYP3A4/CYP2C19 inducer → lower CBD and active-metabolite exposure	Victim	Regulatory labeling (direct)	Label-supported action	Labeling statement; underlying study design and precision not separately reported here.	[5]
CYP2C9 inhibition → higher nabiximols THC (AUC +32%) and 11-OH-THC (AUC ≈ 2.5×)	Victim	Controlled PK study in labeling (direct)	Label-supported action	Pharmacological inhibition, not genotype; oromucosal product co-formulated with CBD, so the magnitude cannot be assumed to transfer to oral capsules.	[3]
Strong CYP3A inducer or inhibitor → altered nabiximols THC, CBD, 11-OH-THC	Victim	Controlled PK study; regulatory labeling (direct)	Label-supported action	Controlled studies reported in labeling; analyte-specific magnitudes vary; one source with the labeling.	[3,38]
Nabiximols → possible CYP1A2/CYP2B6 induction (in vitro, mRNA level); no human substrate exposure measured	Perpetrator	In vitro mRNA induction at clinically attainable concentrations, reported in labeling (mechanistic; not direct for substrate exposure)	Research only	In vitro mRNA induction only; no human substrate exposure measured; clinical relevance unestablished.	[3]
Severe renal impairment → higher nabiximols 11-OH-THC and 7-OH-CBD (3–4.5×) (d)	Victim (contextual)	Dedicated PK study in labeling (direct)	Label-supported action	Contextual modifier, not a convergent modifier; single dedicated study; renal-impairment population only.	[3]
CYP2C19/CYP3A variation → CBD and 7-OH-CBD exposure (c)	Victim (host genotype)	Small pharmacogenomic secondary analysis; supporting in vitro (direct)	Research only	Secondary analysis of a single-center trial, 33 participants with pharmacokinetics in 30; shares a parent trial with the tacrolimus row (footnote c); direction unexplained; concordant with an external regulatory pharmacology dataset but not independently replicated in a prospective study.	[39,40]
CBD → higher international normalized ratio (warfarin); mechanism unassigned	Perpetrator	Case reports (indirect overall) (b)	Author-generated vigilance	Case reports only; no controlled exposure data; mechanism unassigned; confounding by indication and comedication not excluded.	[20,41,42,43]
High-dose CBD-dominant extract → higher THC exposure; relevance to nabiximols untested	Perpetrator	High-dose CBD-dominant extract, not nabiximols (highly indirect)	Research only; potential intra-product interaction	High-dose CBD-dominant extract, not an approved product; dose and composition not comparable to nabiximols; single report.	[44]
(**B**) Untested convergence hypotheses					
Genotype × comedication strata → heterogeneous exposure (direction and magnitude stratum-specific)	Both branches	Mechanistic hypothesis; no direct data (indirect)	Research only	No direct data; stratum-specific direction and magnitude are predicted, not measured; no approved-product study identified in this review has tested the interaction.	Aligned from Table 1A
Real-world safety signals as prioritization signals for exposure hypotheses	Both branches	Pharmacovigilance disproportionality (indirect)	Research only	Disproportionality signals are not measures of exposure or of causation; reporting and indication bias not excluded; used only to prioritize questions.	[10,11]

Footnotes. (a) Directness follows Section 2: direct denotes evidence from the approved product, though not necessarily at labeled doses; partially direct denotes evidence from the approved active ingredient or a closely related clinical formulation; indirect denotes unregulated or non-approved preparations; dose and population bear on applicability rather than on directness (Section 2). (b) Includes two approved-product case reports; the broader supporting literature predominantly concerns other cannabis products. (c) The CBD-tacrolimus row and the CYP2C19/CYP3A row derive from the same single-center, open-label, three-period, fixed-sequence crossover phase I trial of cannabidiol and tacrolimus, which enrolled 57 participants across arms defined by kidney function and CYP3A5 expresser status (ClinicalTrials.gov NCT05490511) and is now completed. They are not independent lines of evidence. The CBD-tacrolimus row reports a prespecified interim analysis of the 12 participants who had completed all three periods; the CYP2C19/CYP3A row is a genotype-stratified secondary analysis of cannabidiol pharmacokinetics in the 33 participants who had received at least one cannabidiol dose, with paired single-dose and steady-state pharmacokinetic sampling in 30 and adverse-event reporting in all 33 participants. The two analyses therefore draw on overlapping subsets of differing size within one trial. (d) Organ function is a contextual modifier, not one of the two convergent modifiers; the row is included because it demonstrates analyte-specific divergence at label level. (e) Key limitations and uncertainty are recorded for the principal design, population, precision, replication, and dataset-overlap constraints bearing on each relationship, as detailed in Supplementary Table S2. No summary confidence grade is assigned. A labeled statement and the study on which it rests are counted as one source, not as independent replication. Limitations are reported independently of directness and of actionability; hypothesis-generating is an evidence role recorded under actionability as research only.

## 3. Cannabinoid Pharmacology and the Determinants of Exposure

The determinants of pharmacologically relevant systemic exposure must be specified before its variability can be examined. Both directions apply: victim exposure to the cannabinoid and its active metabolites, and perpetrator exposure to a co-administered drug or its active metabolite. Genotype and comedication are the core convergent modifiers in both, proposed to act on shared metabolic and, on more limited evidence, transport pathways; food, formulation, route, hepatic function, inflammation, age, dose, adherence, and intracellular binding-protein capacity are contextual modifiers that alter a convergence’s magnitude or interpretation without forming part of it. This section maps them across the four products. The metabolic architecture underlying these differences is summarized in Figure 2.

### 3.1. Cannabidiol

CBD is lipophilic, with low, variable oral bioavailability from incomplete absorption and first-pass metabolism [14,45]. Absorption is food-dependent: compared with fasting, a high-fat, high-calorie meal raises the area under the concentration–time curve (AUC) approximately fourfold and peak concentration approximately fivefold, while reducing variability [5,46,47,48,49]. This equals or exceeds the magnitude of genotype and interaction effects, so meal composition and timing must be standardized or recorded and modeled in genotype-stratified interaction studies, lest prandial state generates or masks target differences.

Parent CBD alone does not fully represent pharmacologically active exposure. CBD is metabolized to 7-hydroxy-cannabidiol (7-OH-CBD), an anticonvulsant in preclinical seizure models at potency comparable to the parent [39,50], then to 7-carboxy-cannabidiol (7-COOH-CBD), whose pharmacological status is unsettled. The labeling at primary analysis described 7-COOH-CBD as inactive [51]; the current revision reports it as anticonvulsant in one of two mouse models of acute seizure, though its relevance to human epilepsy is unknown [5]. It is excluded from the pharmacologically active species here because its activity is uncharacterized rather than absent, and that exclusion is provisional. The exclusion is also narrowly scoped. The pharmacologically active species is an efficacy construct, defined by activity at the therapeutic target, and membership of it governs only which analytes enter the parent–active metabolite profile and the predictions built on that profile. It carries no implication that an excluded metabolite is pharmacologically inert or toxicologically unimportant. 7-COOH-CBD illustrates the distinction: it is not counted toward therapeutic exposure, yet it is a P-glycoprotein substrate and inhibits BCRP- and BSEP-mediated transport at clinically relevant concentrations, which bears on canalicular and cholestatic liability and on bile-transporter interactions rather than on anticonvulsant effect (Section 4.1 and Section 5.5). Its transporter liability is therefore carried in the safety and transporter analyses, not in the active-species definition, and the two should not be read as one judgment. These transporter findings support a potential safety mechanism; they do not establish that 7-COOH-CBD causes cholestatic injury in patients. 7-COOH-CBD is nonetheless the most abundant circulating species by a wide margin: its geometric mean AUC to infinity exceeded that of parent CBD approximately 34-fold after a single 200 mg dose in adults with normal hepatic function [52], and it was reported 47-fold higher than CBD at steady state on 1500 mg twice daily, a dose above the labeled range, where it accounted for about 97% of the drug material measured [47]. Were it shown to contribute materially, both the operational definition and the parent-to-metabolite reasoning used here would require revision. The labeling reports that 7-OH-CBD circulates with an AUC roughly 38% lower than that of the parent compound, and that 7-COOH-CBD circulates at approximately 40-fold higher levels [5]. Reasoning from the parent alone is therefore inadequate, and three quantities must be kept apart. Parent exposure is the systemic concentration of CBD itself. Metabolite formation is the extent of conversion to 7-OH-CBD, governed by different enzymes and able to move opposite to parent exposure within one individual. Total pharmacological contribution is the amount the two species jointly produce at the target; it is the only one of the three that is currently not quantifiable, because no method has been validated to translate combined parent and active-metabolite concentrations into clinical effect. Exposure–response analyses have been reported for cannabidiol against seizure and adverse-event endpoints, but they relate single-analyte exposure to outcome and do not supply a combined parent–metabolite measure. That absence is the governing constraint on what the profile can support: a measured divergence between parent and metabolite is interpretable in direction, and in which analyte moves, but cannot, by itself, be converted into a target concentration, a dose adjustment, or a quantitative estimate of clinical consequence. The limit applies wherever such divergence is reported below and is not restated at each occurrence. The parent and its pharmacologically active metabolite are collectively referred to as the parent–active metabolite profile. This is never a summed or weighted quantity, and the two species are always measured and reported separately.

These enzymatic routes define where exposure is governed. CBD undergoes extensive oxidative metabolism, principally by CYP2C19 and CYP3A4, with glucuronidation via UGT1A7, UGT1A9, and UGT2B7 [5,53,54,55]. Reaction phenotyping indicates 7-OH-CBD is formed chiefly by CYP2C19, with CYP3A4 acting largely at other oxidation sites [54]. Because CYP2C19 is highly polymorphic and CYP3A4 is a frequent interaction target, these are the nodes at which inherited variation and comedication alter CBD exposure; their relative contributions are addressed in Section 5.2. Organ function bounds these routes asymmetrically: exposure to total CBD rises with hepatic impairment, the geometric mean ratio relative to normal function being 1.48 (90% CI 0.90–2.41) in mild, 2.45 (1.50–4.01) in moderate, and 5.15 (2.94–9.00) in severe impairment, with metabolite exposures rising less than the parent [52], whereas mild to severe renal impairment alters neither parent nor metabolite exposure [56]. The contrast is informative for the framework, since severe renal impairment raises nabiximols metabolite exposures several-fold (Section 3.2).

CBD occupies both branches. As a CYP2C19 and CYP3A4 substrate, it is primarily cleared by these enzymes, so inducers or inhibitors alter its exposure [5]. As a perpetrator, it inhibits several CYP enzymes in vitro, with CYP2C19 being the clinically consequential node, notably in the CBD–clobazam interaction [57], thereby increasing exposure to the active metabolite, N-desmethylclobazam [31]. Quantitative treatment is reserved for Section 6.

### 3.2. THC-Containing and THC-Analog Medicines: Dronabinol, Nabilone, and Nabiximols

Dronabinol is synthetic Δ^9^-THC, extensively metabolized and, like CBD, of low oral bioavailability (approximately 10% to 20%) from pronounced first-pass metabolism [12,29]. Nabilone is a synthetic THC analog distinct from dronabinol and, like it, given orally as a capsule; it is neither derived from nor converted to THC, so dronabinol’s disposition and bioavailability do not transfer [6]. Its parent half-life is short while total radioactivity (all labeled species, not active-metabolite exposure) persists much longer, so effects may outlast the parent. Which nabilone metabolites are active is not established. THC is a substrate of CYP2C9 and CYP3A4, converted to psychoactive 11-hydroxy-THC (11-OH-THC) and thereafter to 11-nor-9-carboxy-THC (THC-COOH) [58,59,60]. THC-COOH is non-psychoactive and is not among the pharmacologically active species, unlike parent THC and 11-OH-THC, but is measured throughout as a disposition marker for the same CYP2C9-mediated step, the pharmacogenomically consequential node (Section 5.1). What distinguishes these from CBD is not exposure variability but its consequences: raised THC exposure may translate more readily into dose-limiting psychoactive effects than an equivalent rise in non-psychoactive CBD.

Nabiximols occupies an intermediate position: among the approved THC-containing products it has direct product-specific victim-interaction evidence, unlike the indirect high-dose extract evidence sometimes used to infer its profile. The botanical oromucosal spray delivers 2.7 mg THC with 2.5 mg CBD per 100 µL actuation, and oromucosal absorption reduces, though it does not eliminate, first-pass metabolism [3,61]. Exposure is strongly food-dependent, with CBD AUC rising approximately fivefold [3,62].

Three further product-specific features documented in the labeling bear on the question of the analyte. Interindividual variability is high and, unusually, quantified; a dedicated hepatic impairment study permits mild impairment without adjustment while advising against moderate or severe impairment; and a severe renal impairment study found the active metabolites 11-OH-THC and 7-OH-CBD rose approximately three- to four-and-a-half-fold while parent exposure rose approximately twofold for CBD and less than twofold for THC, so lower daily doses may be needed [3]. The renal result is the clearest product-level demonstration in any approved cannabinoid that a single modifier can raise active-metabolite exposure substantially more than parent exposure, here roughly twice as much again. Organ function is a contextual rather than a convergent modifier, but the finding demonstrates, at the label level and in a THC-containing product, that parent concentration can be an unreliable proxy for active-metabolite exposure.

The two THC products differ in exposure, not merely formulation. Oral dronabinol undergoes first-pass metabolism, so bioavailability is low, and much becomes 11-OH-THC, so the metabolite contributes proportionally more to active exposure than after routes that bypass part of that first pass. CYP2C9 acts at that conversion step, which is the basis for dronabinol’s one label-level pharmacogenomic signal (Section 5.1). Oromucosal nabiximols bypass part of that first pass, which offers a plausible reason to expect proportionally less 11-OH-THC from a given THC dose. That expectation is not established, and the one direct human comparison does not support it: in a randomized, double-blind, double-dummy crossover in nine cannabis smokers, similar doses of synthetic oral THC and of the oromucosal extract produced no statistically significant differences in maximum plasma concentration, time to maximum concentration, or area under the curve to 10.5 h; the analytes quantified included 11-OH-THC, and the relative bioavailability of oral THC was between 93% and 99% of the corresponding oromucosal dose [63]. Nine participants cannot establish equivalence. The study compared oral synthetic Δ^9^-THC with the oromucosal extract under controlled conditions; the report does not identify the oral product as the approved dronabinol capsule, so the comparison is partially direct for dronabinol under the definitions in Section 2, and its applicability to that product is unconfirmed. The swallowed fraction, prandial state, dose, and dosing regimen all matter, and the findings should not be generalized across them. The route-based expectation is therefore a plausible explanation, not a property of the products. Two consequences are correspondingly unestablished: whether a CYP2C9 modifier shifts the parent–active-metabolite profile differently in nabiximols and dronabinol is a testable prediction rather than a finding; and nabiximols alone co-administer CBD, an inhibitor of THC-clearing enzymes, so its intra-product pharmacokinetics have no dronabinol counterpart. Evidence maturity runs opposite to route: dronabinol has the pharmacogenomic signal but no product-specific interaction data; nabiximols has the reverse.

The intra-product question remains open: because nabiximols co-administers CBD with THC, CBD may, in principle, raise THC exposure within the product itself. No study testing this in nabiximols was identified in this review, and the nearest indirect comparator addresses a different question.

## 4. Post-Approval and Real-World Safety Evidence

Registration trials establish a controlled-conditions safety profile but cannot represent routine-use heterogeneity. This section defines the outcomes that pharmacogenomic and drug-interaction analyses must explain, using controlled trials, reviews, observational studies, and pharmacovigilance, and closes by ordering them by proximity to a measurable exposure step. Coverage is selective and mechanism-oriented: substantial for prescription CBD, class-level for cardiovascular signals, and sparse elsewhere. The FDA Adverse Event Reporting System, EudraVigilance, and VigiBase receive reports from genetically diverse, polymedicated, off-label populations and may surface signals that are undercharacterized in trials [64,65]. In one tertiary-center cohort, 64.8% of adults given pharmaceutical CBD received it outside authorized indications [66]. Not every outcome is exposure-mediated: some reflect pharmacodynamics, comedication, or underlying disease.

### 4.1. Hepatic Effects and the Valproate–Clobazam Axis

Dose-dependent elevation of serum aminotransferases is the principal hepatic concern for CBD, evident in registration trials, occurring more frequently at higher doses and with concomitant liver-affecting antiseizure medications [67,68,69]. At CBD 10 to 20 mg/kg/day, alanine aminotransferase elevations exceeding three times the upper limit of normal occurred in approximately 4% of patients taking clobazam without valproate, 21% taking valproate without clobazam, and 30% taking both, compared with 3% taking neither [5]. Valproate is therefore the dominant modifier; clobazam’s incremental contribution is smaller, not absent. CBD does not alter systemic valproate exposure, nor does valproate materially alter CBD exposure [5,31,70], so the amplified hepatotoxicity is not explained by the measured systemic pharmacokinetics of either drug. The labeling adds a further measured negative: CBD reduced exposure to the putative hepatotoxic valproate metabolite by approximately 28% at maximum concentration and 33% in AUC, so its accumulation is not the explanation, either. Quantitative systems toxicology modeling of bile acid transporter inhibition, mitochondrial dysfunction, and reactive oxygen species production failed to reproduce the clinical signal [71], narrowing the candidate routes rather than excluding all of them, and a three-dimensional human hepatocyte spheroid model has since reproduced the enhanced injury, identifying a tractable in vitro system rather than a mechanism [72]. The interaction is therefore clinically established, unexplained by measured systemic pharmacokinetics, and mechanistically unresolved.

This is the framework’s boundary condition. Comedication-related safety risk can rise without a measured increase in systemic cannabinoid exposure, so the co-medication milieu rather than systemic CBD exposure appears to be the major determinant of hepatic risk here. The claim is narrower than it may appear; it does not exclude an exposure-related mechanism operating where measurements do not reach, in intrahepatic or subcellular compartments, unmeasured metabolites, or short-lived reactive intermediates, nor a toxicodynamic interaction at the hepatocyte level. It limits the systemic-exposure framework, not the relevance of exposure to this outcome. A phase 1 trial in sixteen healthy adults taking no antiseizure medication bears on this boundary from the other side: seven exceeded the upper limit of normal for alanine aminotransferase, and five met consensus criteria for drug-induced liver injury at CBD 1500 mg/day, with no correlation to CYP2C19 genotype or to CBD plasma concentration [73]. Hepatic susceptibility therefore varies between individuals for reasons that neither genotype nor measured systemic exposure explains, which places a second limit on what an exposure-based framework can predict for this outcome.

Consistent with this, the prescribing information recommends monitoring serum transaminases and total bilirubin before treatment and periodically thereafter, with vigilance for concomitant valproate or clobazam [5]. The labeling also records post-marketing reports of elevated ammonia in CBD-treated patients with transaminase elevations, most of whom, where data existed, were taking valproate, clobazam, or both, and advises discontinuing or dose-adjusting either if ammonia rises, reproducing the comedication dependence in a second parameter. These reports are uncontrolled and provide no incidence data.

A second hepatic phenotype has emerged post-approval. The labeling records post-marketing cases of cholestatic and mixed liver injury, classified by the alanine aminotransferase-to-alkaline phosphatase ratio, expressed as multiples of the upper limit of normal: below 2, cholestatic; 2 to 5, mixed [5]. These uncontrolled reports support neither causal attribution nor incidence estimation, and the phenotype differs from that of the controlled trial and from the quantitative systems toxicology endpoint, which assessed bile acid transporter inhibition relative to the valproate hepatocellular signal [71]. Unlike the valproate interaction, it poses a potential exposure risk: 7-COOH-CBD inhibits bile salt export pump (BSEP)-mediated transport at clinically relevant concentrations (Section 5.5). The hypothesis is untested.

### 4.2. Neuropsychiatric and Sedative Effects

Somnolence and sedation are consistent adverse effects of CBD-, THC-containing, and THC-analog medicines in controlled trials [4,67,69], and real-world data largely confirm this profile. One pharmacokinetically mediated contribution is worth isolating: a perpetrator-branch example in which the altered exposure is to the active metabolite of a co-administered drug rather than to the cannabinoid itself. CBD raises the active metabolite N-desmethylclobazam approximately 3.4-fold in healthy volunteers [31], and an open-label cohort of CBD added to established antiseizure regimens found sedation significantly more frequent at higher serum N-desmethylclobazam concentrations (*p* = 0.02) [74]; it is accordingly listed in the labeling among reactions for which clobazam co-administration warrants attention [5]. This single-cohort signal links to the perpetrator interaction of Section 6.1 and suggests that the operative quantity is active-metabolite exposure rather than co-prescription alone. Among the neuropsychiatric outcomes here, sedation has the clearest defined PK pathway.

In contrast, neuropsychiatric findings resting solely on pharmacovigilance data lack an established PK mechanism. A VigiBase disproportionality analysis restricted to five preferred terms selected a priori for serotonergic plausibility covered 820 of 12,702 CBD reports, 91.8% of which named Epidiolex, and returned significant signals for weight decreased (reporting odds ratio 5.19), hypophagia (3.68), and insomnia (1.60), whereas dizziness and palpitations were not disproportionately reported [11]. No metabolic pathway, genotype, or analyte has been linked to any of these signals, and none has been shown to be exposure-mediated. The two groups therefore sit at different distances from the exposure axis.

### 4.3. Cardiovascular and Fall-Related Outcomes in Older Adults

The most clinically relevant cardiovascular signal here is autonomic rather than ischemic. A meta-analysis of 47 randomized trials of medical cannabinoids (2800 patients, median cannabinoid exposure 15.8 days) associated cannabinoid use with orthostatic hypotension (risk ratio 3.16) and hypotension (risk ratio 3.55), with only a non-significant trend toward tachycardia [75]. No included study reported a serious cardiovascular event, 45% of trials excluded patients with pre-existing cardiovascular disease, and the pooled estimates are limited by source-trial quality. Because agents with differing cardiovascular profiles were pooled, the signal is best read at class level rather than attributed to THC or CBD individually, and because the pool is defined by drug class rather than regulatory status, the estimate is indirect for all four approved medicines (Section 2).

These autonomic effects matter most in older adults, underrepresented populations, and frail or fall-prone patients. A systematic review of THC-containing medicines in adults aged 50 and older advised caution because dizziness and light-headedness may increase fall risk [8], reinforced by frequent dizziness and drowsiness reports with dronabinol and nabilone [4]; risk may compound where sedation coincides with orthostatic change and sedatives, antihypertensives, or opioids are co-prescribed. Falls are multifactorial, and the source studies measured no exposure, so these outcomes are retained as lower-proximity exposure hypotheses rather than assessed here, although the orthostatic effect should track exposure if measured. The meta-analysis linked oral administration to more pronounced reactions than non-oral, but route covaries with product, dose, indication, and population, so first-pass variability is one account among several that the data cannot distinguish and should not be read as established.

### 4.4. Database Evidence, Its Limits, and Signal Prioritization

The pharmacovigilance signals reviewed above derive from disproportionality analyses, which quantify whether an event is reported more often than expected for a drug relative to the database. These measures do not estimate incidence, absolute risk, or causation, and are susceptible to reporting bias, confounding by comedication and comorbidity, the absence of a defined denominator, and variable event coding; reporting standards have been developed in response [64,65], and a recent cross-national analysis of nearly 10,000 cannabinoid-related reports illustrates both the reach of these databases and their limits [76]. More decisive is what they do not record: genotype, measured cannabinoid or metabolite concentrations, reliable dose and timing, confirmed product composition, or complete comedication histories, the variables on which the framework depends. These signals are therefore hypothesis-generating.

What they can do is set priorities. If exposure is the variable and genotype and comedication are its modifiers, post-approval reports are best read as clinical signals that help prioritize exposure-focused hypotheses in populations far more heterogeneous than any trial cohort. The ordering that follows is an author-generated research-prioritization hierarchy arranged by proximity to a measurable exposure pathway; it is not a validated evidence scale or a causal ranking, since proximity describes how readily an outcome could be tested against exposure, not how likely it is to be drug-caused. Highest priority attaches where a quantified exposure step and a narrow therapeutic margin coincide, as in the CBD interactions with tacrolimus and everolimus, both resting on controlled PK evidence with the approved product, though those studies measured exposure rather than toxicity. Sedation with CBD and clobazam ranks alongside them, for the reasons given in Section 4.2. Loss of efficacy under strong induction ranks next, plausibly an exposure signal in the opposite direction but also attributable to nonadherence, disease progression, and misattribution. Falls in older adults and insomnia- and weight-related signals rank below both due to the lack of a measured exposure step. Valproate-associated hepatic injury falls outside the ordering rather than at its foot, being the framework’s boundary condition.

The claim made here is correspondingly narrow. A genetically unselected pivotal trial, powered for average effects, is by design poorly placed to detect events concentrated in the tails of an exposure distribution, should they be so concentrated, which for these medicines is untested. Pharmacovigilance can identify which outcomes merit prospective, exposure-measured investigation once a medicine reaches a broader population; it cannot test genotype, measure exposure, or establish causality. Among the many determinants of the variability these signals reflect, the two sources whose hypothesized mechanistic convergence is examined next are host genetics and concurrent medication, taken in turn, beginning with the pharmacogenomic determinants of cannabinoid exposure.

## 5. Pharmacogenomic Determinants of Cannabinoid Exposure and Response

Host genotype can materially contribute to exposure variability at selected pathways, acting chiefly in the victim branch (altering cannabinoid and active-metabolite exposure) but also in the perpetrator branch, where a polymorphic enzyme sets the co-substrate’s baseline clearance (CYP3A5 and tacrolimus). Genotype is the least mature of these determinants; clinical studies are few, small, and mostly in healthy volunteers, so the section is organized by evidence strength rather than by enzyme. The set of pharmacologically active species is as defined above, extended to include THC-containing products: parent CBD with 7-OH-CBD and parent THC with the psychoactive 11-OH-THC, whose contribution may depend on route and formulation; a greater contribution after oral dronabinol than after oromucosal nabiximols has not yet been established. Because a genotype that slows parent clearance may also slow active metabolite formation, the two may move in opposite directions within one individual, so genotype effects are reported below for each analyte.

### 5.1. Label-Relevant Pharmacogenomics: CYP2C9 and Orally Administered THC

The best-characterized cannabinoid pharmacogenomic signal concerns oxidation of THC by CYP2C9, the more developed of two label-level genotype-linked statements (the second, a bilirubin caution in the nabiximols labeling). Two usages must be kept apart: oral THC denotes THC given by mouth in a study setting, as below, which does not specify a marketed formulation, whereas dronabinol denotes the approved capsule, an extrapolation across formulations the sources do not close. In 43 healthy volunteers selected from a larger pool to enrich for the variant genotypes and given a single oral 15 mg THC dose, the four CYP2C9*3/*3 homozygotes showed a median THC AUC approximately threefold higher, and THC-COOH exposure approximately 70% lower, than the nineteen CYP2C9*1/*1 homozygotes, with higher sedation scores, an association the investigators judged unproven given the limited sample size and nonblinded design; THC pharmacokinetics did not differ by CYP2C9*2 status, though that comparison was drawn across carriers and provides weaker evidence of no effect [28]. The opposing directions of parent and carboxy metabolite fix the interpretation: reduced CYP2C9 activity raises parent THC while lowering carboxy-metabolite formation, so parent THC, the psychoactive 11-OH-THC, and THC-COOH are three separate analytes. The active metabolite bears directly on the framework: 11-OH-THC did not fall with reduced CYP2C9 function but tended to rise without reaching significance, and when parent and active metabolite were summed, as the investigators did, reasoning that the sum may reflect the pharmacologically active principle, CYP2C9*3/*3 homozygotes attained nearly twice the concentrations of CYP2C9*1/*1 homozygotes [28].

This is the only human genotype-stratified evidence that reduced function may increase combined parent and active-metabolite exposure to the active ingredient of an approved cannabinoid medicine. It rests on a four-participant subgroup; the metabolite difference did not reach significance, and the summed measure is exploratory rather than validated, so the genotype-to-active-metabolite relationship is inadequately characterized rather than established. This is also why the three analytes must be specified separately in any study design.

The in vitro investigators drew a parallel caution: CYP2C9.2 and CYP2C9.3 retain about one third of wild-type efficiency for THC 11-hydroxylation, yet the consequences are hard to predict because both parent and 11-hydroxy metabolite possess psychotropic properties, the latter reported as indistinguishable in activity from the parent, though not necessarily solely responsible for it [58,77].

This maps onto the approved product: the dronabinol prescribing information states that dronabinol is metabolized principally by CYP2C9 and CYP3A4, that inhibitors and inducers may alter its exposure, and that individuals with genetic variants associated with diminished CYP2C9 function may show a two- to threefold higher dronabinol exposure, with monitoring for increased adverse reactions recommended [29]; mechanistically, this reflects reduced CYP2C9-mediated first-pass and systemic metabolism of orally administered THC. The in vitro data are a poor guide to the in vivo result: expressed CYP2C9.2 and CYP2C9.3 both retain approximately 30% of wild-type intrinsic clearance, yet only *3 produces a measurable in vivo difference [28,58], a discordance that parallels the CYP2C19 inversion in Section 5.2. Whether CYP2C9 acts primarily in the formation of 11-OH-THC or in its subsequent oxidation was long disputed [28,78]; systematic reaction phenotyping now assigns the formation step predominantly to CYP2C9, as set out below, while the subsequent oxidation step remains unresolved. This divergence between parent and metabolite is shown in Figure 2.

Box 1 **Key falsifiable predictions of the convergent exposure-modifier (CEM) framework.**

The framework’s central prediction is that pharmacologically relevant exposure, whether of the cannabinoid and its active metabolites, or of a co-administered substrate and its active metabolite, varies systematically across genotype and comedication combinations, and that those combinations explain exposure better than either modifier alone. Two distinct claims are involved and are tested separately. Effect modification, that the treatment-associated change in exposure differs by genotype, is what P1 to P3 test. Incremental explanatory value, that genotype and comedication together predict exposure better than either alone, is what P5 tests; two independent main effects could satisfy it without any interaction, so the two criteria are not interchangeable. This review identified no approved-product study testing either claim in an approved cannabinoid; the predictions below are assembled from independently supported observations (Table 1).

Each is stated as population, exposure endpoint, statistical test, and the result that would weaken or refute it; where relevant, a further line gives the mechanistic basis or the principal constraint. The core predictions are each framed around a within-patient change, because between-group differences measured only after treatment cannot separate a genotype main effect from the predicted interaction. Pathway rationale is given in Section 5 and Section 6; study design, feasibility, and priority in Section 8.2; neither is repeated here. No prediction is framed for nabilone, whose active metabolites are uncharacterized, or for nabiximols, for which no genotype-stratified pharmacokinetic data were identified.

Core predictions

**P1. CYP2C19, CBD, and clobazam, perpetrator branch. Population:** clobazam-treated patients, CYP2C19-genotyped before CBD is introduced. Exposure endpoint: within-patient change in dose-normalized N-desmethylclobazam exposure, analyzed on the log scale so that the treatment effect is expressed as a fold change, with parent clobazam, CBD, and 7-OH-CBD measured alongside. Test: genotype-by-treatment interaction across CYP2C19 phenotype groups; the same term distinguishes the multiplicative from the residual-enzyme model under the assumptions set out in Section 5.6, although an interaction test alone does not uniquely identify the underlying mechanism. Weakened if: the interaction term is compatible with no effect modification after adjustment for baseline exposure and treatment factors, with a confidence interval that excludes differences in the size that would matter clinically; a non-significant term alone does not establish independence. Bounded by: CBD is itself a CYP2C19 inhibitor, and the clobazam interaction is the in vivo demonstration of that inhibition (Section 6.1.1), so the treatment provoking the tested change also phenoconverts the strata being compared. After treatment, genotype-inferred status loses predictive value as inhibition approaches completeness, which is why the prediction is framed on the within-patient change. That contrast need not be abolished under the stated residual-enzyme assumptions, since it is generated by how much CYP2C19-mediated clearance each stratum has to lose (Section 5.6). However, it is compressed where inhibition is partial or variable across the titration range. Because the endpoint is dose-normalized, clinical clobazam dose reduction does not by itself erase the interaction under linear, steady-state conditions; it complicates estimation through the timing of the change, incomplete re-equilibration, and any departure from dose proportionality. On this scale, the multiplicative model corresponds to a null genotype-by-treatment interaction and the residual-enzyme model to a smaller fold change in reduced-function carriers, so the study must be powered on a prespecified residual-enzyme interaction effect. The sample size required follows from that effect size together with within- and between-patient variability, genotype frequencies, the inhibitor exposure actually achieved, and the extent of clinical clobazam dose adjustment, rather than from the choice of model alone; enrichment for reduced-function carriers (Section 8.2) is necessary but may not be sufficient. Sampling across the titration range rather than at a single steady-state dose, recording CBD and 7-OH-CBD as measures of inhibitor exposure, which index but do not directly quantify the degree of CYP2C19 inhibition achieved, and modeling clobazam dose changes keep the contrast interpretable. A null interaction term is correspondingly not uniquely interpretable: it is consistent with absent effect modification, with an interaction too small to resolve at the achieved precision, and with estimation distorted by time-varying dose adjustments, incomplete re-equilibration, or departures from dose proportionality.

**P2. CYP2C9, dronabinol and a CYP2C9 inhibitor, victim branch. Population:** CYP2C9-genotyped dronabinol recipients given a clinically relevant CYP2C9 inhibitor such as fluconazole. Exposure endpoint: within-patient change in parent Δ^9^-THC, 11-OH-THC, and THC-COOH, each measured separately. Test: genotype-by-inhibitor interaction, tested per analyte. Weakened if: no analyte shows a difference in inhibitor-related change across CYP2C9 strata; a genotype main effect alone would not demonstrate effect modification, and independence cannot be inferred from a non-significant interaction term, whose interpretation depends on the confidence interval and on whether clinically meaningful differences have been excluded. Bounded by: no available inhibitor is CYP2C9-selective, and the genotype signal derives from oral Δ^9^-THC of unspecified formulation, and its applicability to the approved dronabinol capsule is unconfirmed (Section 5.1). Mechanistic basis: human enzyme data predict that THC is cleared mainly by CYP3A and CYP2C19, where CYP2C9 function is reduced [79].

**P3. CYP3A5, CBD, and tacrolimus, perpetrator branch. Population: tacrolimus-treated patients, CYP3A5 expresser status recorded, before and after CBD initiation. Exposure endpoint:** within-patient change in dose-normalized tacrolimus exposure. Test: whether the magnitude of the CBD-related change differs between CYP3A5 expressers and non-expressers, with baseline exposure incorporated. Weakened if: the difference is compatible with no effect modification and its confidence interval excludes clinically meaningful differences; a null result could not distinguish absent convergence from a small CYP3A5 contribution, because the mediating pathway is undetermined (Section 6.1). Note: this prediction concerns the magnitude of an interaction already established, not whether it occurs. It is the most exploratory of the three mechanistically, and the most actionable clinically; tacrolimus is the only substrate here for which a measured interaction magnitude, a regulator-issued interaction action, and a guideline-issued genotype recommendation coexist.

**Secondary and framework-level predictions**

**P4. CYP2C19 and CYP2C9 acting jointly on CBD, gene–gene extension; not a test of the central proposition. Population:** CBD-treated patients genotyped for both enzymes. Exposure endpoint: the 7-OH-CBD-to-CBD ratio; because both species are active, no direction is predicted for the parent–active-metabolite profile overall. Test: whether CYP2C9 genotype adds explanatory value to CYP2C19 status. Weakened if: CYP2C9 adds nothing to CYP2C19 in explaining the ratio. This rests on an in vitro compensation hypothesis discordant with the only human observation available (Section 5.2). That discordance is a source of uncertainty about the hypothesis rather than a basis for expecting a particular result, and either outcome would be informative.

**P5. Incremental explanatory value of genotype–comedication strata, framework-level criterion. Population:** linked prospective datasets carrying genotype, dose, formulation, comedication, measured exposure and outcomes. Exposure endpoint: variability in pharmacologically relevant exposure in whichever branch is under test. Test: prespecified model comparison with out-of-sample validation, not improvement in apparent fit. Refuted if: combined strata do not improve out-of-sample explanation of measured exposure. Spontaneous reports cannot test this, carrying neither genotype nor exposure.

**Confirmation of these predictions would move the corresponding links from hypothesis toward evidence.**

A systematic reaction phenotyping study provides stronger evidence for assignment of the formation step and extends the framework. Across twelve recombinant enzymes, pooled human liver microsomes, and microsomes from 23 individual donors, CYP2C9 was confirmed as the principal route to 11-OH-THC, with a selective inhibitor reducing its formation by 65% to 78% in six of those donors, while CYP3A4 and CYP3A5 formed three further metabolites tentatively assigned to the cyclohexenyl ring; for CYP3A4 the unbound Michaelis constants, and the half-maximal constant for the one metabolite showing allosteric kinetics, were more than eightfold higher than for CYP2C9 or CYP2C19, from which the investigators expect the CYP3A contribution to rise at higher THC concentrations [79]. CYP2C19 formed 11-OH-THC with a turnover number comparable to CYP2C9 (14 versus 12 min^−1^) and an unbound intrinsic clearance roughly threefold lower, and contributed alongside CYP3A to one of the ring-hydroxylated metabolites, predominating in high-CYP2C19 donors; this is the first reported CYP2C19-mediated formation of a THC metabolite other than 11-OH-THC [79]. Two findings bear directly on convergence. First, CYP2C19 inhibition reduced 11-OH-THC formation by up to approximately 17% and did so most in the donors with high CYP2C19 activity, precisely those in whom CYP2C9 inhibition mattered least, so CYP2C19 may sustain part of active-metabolite formation where CYP2C9 function is low. The investigators explicitly state the consequence: in individuals carrying CYP2C9 variants, THC is predicted to be cleared primarily by CYP3A and CYP2C19, so inhibitors of those enzymes would be expected to reduce THC clearance [79]. That is a drug–gene–drug convergence prediction for the dronabinol victim branch, derived from human enzyme data rather than from the one proposed here, and it provides the mechanistic basis for prediction P2 in Box 1. Second, on this evidence, CYP2C19 is a clearance node shared by both cannabinoids rather than a CBD-specific one: a single reduced-function genotype would affect 7-OH-CBD formation and 11-OH-THC formation in different branches of the framework, which matters most for nabiximols, where both cannabinoids are co-administered. The evidence is in vitro throughout, and no in vivo study identified in this review has tested either consequence in an approved product.

The same work identifies a determinant that the framework does not currently accommodate. Liver fatty acid binding protein (FABP1), reported in hepatocyte cytosol at 0.7 to 1 mM, binds Δ^9^-THC, 11-OH-THC, and CBD with sub-micromolar affinity; in human liver microsomes it lowered free Δ^9^-THC by 86% and reduced formation of the two CYP3A4-specific metabolites by 65% to 85%, while the metabolite to which CYP2C19 also contributes fell less (49% to 68%) and 11-OH-THC formation was essentially unchanged (0% to 9%) [79]. Because the suppression is enzyme-selective, FABP1 does not simply scale down clearance; the investigators suggest it may increase the relative contribution of CYP2C9 and CYP2C19 to Δ^9^-THC metabolism, and that other lipophilic CYP3A4 and CYP2C19 substrates that bind FABP1 would be similarly affected. Intracellular binding-protein capacity is therefore a candidate contextual modifier of which enzyme carries a given step, demonstrated only in vitro, is unmeasured in any clinical study reviewed here, and is a source of exposure variability that genotype and comedication alone would not capture.

The CYP2C9 genotype signal is best classified as an established PK effect with label-level monitoring relevance but insufficient for genotype-guided dose adjustment: the underlying study was single, no replication was identified in this review, and neither the label nor guidelines specify a genotype-based dose. The signal is most relevant to oral dronabinol. One line of direct approved-product evidence supports the pathway, if not the genotype: in nabiximols, the CYP2C9 inhibitor fluconazole raised THC AUC by 32% and 11-OH-THC AUC by approximately 2.5-fold [3], the only demonstration in an approved cannabinoid that modulating CYP2C9 alters both THC and active metabolite exposure. Two qualifications bound its use: it is pharmacological rather than genetic and was obtained in an oromucosal product co-formulated with CBD, so its magnitude cannot be extrapolated to an oral capsule. What both products lack is the genotype axis: no genotype-stratified pharmacokinetic study of nabiximols was identified in the searches described in Section 2; a single study relates ABCB1 genotype to clinical response, but measured no cannabinoid concentrations [80]; and nothing here transfers to nabilone, which is not THC and whose active metabolites are unidentified.

### 5.2. Emerging Pharmacogenomics: CYP2C19/CYP3A Variation and CBD Exposure

7-OH-CBD is formed chiefly by CYP2C19, making CYP2C19 metabolizer status a plausible but not sole determinant of parent and active-metabolite exposure. A second enzyme contributes conditionally. Across individual human liver microsomes, CBD 7-hydroxylation correlated highly with CYP2C19 marker activity and was inhibited by omeprazole but not the CYP2C9-selective inhibitor sulfaphenazole [54]; however, in single-donor microsomes selected for low CYP2C19 activity, CYP2C9 inhibition significantly reduced 7-OH-CBD formation, and recombinant-enzyme phenotyping confirmed that both CYP2C19 and CYP2C9 can catalyze the 7-hydroxylation [39]. These complementary observations suggest that CYP2C9 may provide a compensatory route, more apparent when CYP2C19 activity is low. All three lines are in vitro; no in vivo study was identified showing that CYP2C9 sustains 7-OH-CBD formation when CYP2C19 is reduced, and none measured the two genotypes jointly. The compensation is accordingly treated throughout as mechanistic in vitro evidence, generating prediction P4 in Box 1 rather than supporting a claim.

Directionality matters. Because both enzymes catalyze the same formation step, dual reduced function would be expected to slow 7-OH-CBD formation, raising parent CBD exposure and lowering the 7-OH-CBD-to-CBD ratio rather than increasing metabolite exposure. The net consequence for total active exposure is uncertain, since the parent and metabolite are both active and move in opposite directions; the convergence predicts a shift in the parent–active-metabolite profile rather than a determinate change in overall exposure, and CYP2C19 status alone would predict the metabolite-to-parent ratio poorly wherever CYP2C9 function also varies. No study testing this in any approved cannabinoid product was identified in this review.

The available clinical data are not straightforward. A genotype-stratified secondary analysis of a single-center, fixed-sequence crossover phase I trial of cannabidiol and tacrolimus (Table 1, footnote (c)), in which 33 healthy participants received at least one cannabidiol dose and pharmacokinetic parameters were determined in 30, found that, counterintuitively, CBD exposure and parent-to-metabolite ratios varied with CYP2C19 status, both lowest in intermediate metabolizers after a single dose (*p* = 0.0143) and at steady state (*p* = 0.0033) [40]. The same direction appeared in an external validation dataset from the regulatory clinical pharmacology review, so the inversion is not confined to a single cohort; its mechanism remains unexplained, and the authors call it paradoxical. It cautions against deterministic genotype-to-dose inference: an active metabolite, parallel oxidative and glucuronidation routes, and concentration-dependent inhibition of these enzymes by CBD prevent a single genotype from mapping cleanly onto a single change in exposure. Participants received the approved product at 5 mg/kg once and twice daily to steady state, so the evidence is direct for the approved medicine and is classified as direct in Table 1. Its limitations are nevertheless substantial for reasons unrelated to directness: a secondary analysis in those 33 participants, with paired pharmacokinetic sampling in 30, needing replication in patients; pooled metabolizer categories; and one CYP2C19*2/*17 participant classified as normal rather than intermediate on a study-specific rationale from the labeling, not consensus phenotype translation [81], a placement not negligible in groups this size.

CYP3A likewise requires care. CYP3A-mediated oxidation and UGT-mediated glucuronidation both contribute to CBD disposition [54,55], but CYP3A5 genotype should not be presented as an established major pathway comparable to CYP3A4, and its clinical relevance remains preliminary. The CYP3A5 discussion here concerns the disposition of CBD and its metabolites, not its role in the perpetrator interactions discussed in Section 6. Taken together, the CBD pharmacogenomic relationship is biologically plausible but clinically unresolved.

The same study offers a rare genotype–clinical-event link. Diarrhea, the most frequent likely related adverse event, occurred in 7 of 18 participants (39%) who were both CYP3A5 non-expressers and CYP2C19 intermediate or normal metabolizers, compared with 1 of 15 (7%) with other genotype combinations (*p* = 0.0463), the same combination showing higher steady-state exposure to both 7-OH-CBD and 7-COOH-CBD [40]. The association required two genotypes, not one; the CYP2C19 categories could not be separated because of substantial overlap with CYP3A5 non-expresser status. The authors declined to infer causation, suggesting the concentrations might instead reflect intestinal metabolism, since both enzymes are expressed in the gut. This exploratory genotype–metabolite–adverse-event association in a small cohort of healthy volunteers is not causal and does not belong among the monitoring recommendations in Section 8.1.

### 5.3. UGT Pathways: Metabolism, Interaction, and the Pharmacogenomic Gap

Glucuronidation raises three questions at different stages of resolution, so the pathway’s maturity depends on which one is asked. The first, whether UGT enzymes participate in CBD metabolism, is established: UGT1A7, UGT1A9, and UGT2B7 all contribute to CBD glucuronidation [55].

The second question is whether CBD alters exposure of UGT substrates as a perpetrator, and some evidence exists. The labeling identifies CBD as an inhibitor of UGT1A9 and reports a modest interaction with the UGT2B7 substrate zidovudine, whereas in vitro assessment indicates that CBD does not inhibit UGT1A6; recombinant enzyme studies report CBD inhibition of UGT1A9, UGT2B4, UGT1A6, and UGT2B7 in that order of potency [5,82]. Dedicated human probe-substrate studies exist for both isoforms and differ in magnitude: CBD raised mycophenolic acid AUC by 35%, with the labeling advising that a dose reduction be considered for UGT1A9 substrates for which small concentration increases may cause serious adverse reactions, as clinically appropriate, whereas it raised zidovudine AUC by only 19%, judged not clinically significant [5]. The perpetrator component therefore rests on direct human evidence and is isoform-specific: UGT1A9 currently carries label-level actionability that UGT2B7 does not, and the UGT2B7 branch is substrate-dependent: modeling predicts a 20% to 30% rise in hydromorphone exposure under CBD [83], whereas the 19% rise reported for zidovudine is a measured human value [5]; model-predicted and measured exposure changes are not equivalent evidence. Physiologically based modeling of CBD with morphine is consistent with the smaller zidovudine effect [84], and UGT2B7 also mediates glucuronidation of clopidogrel carboxylic acid [85], so the perpetrator question is not confined to morphine.

The third question, whether UGT genotype predicts exposure to CBD or to a co-administered substrate, is unresolved: no clinically interpretable UGT genotype–CBD exposure relationship has been established. One labeling qualification applies. The nabiximols Summary of Product Characteristics reports inhibition of UGT1A9 and UGT2B7 at clinically attainable concentrations and states that patients with genetic glucuronidation disorders, such as Gilbert’s disease, a UGT1A1 variant phenotype, may show elevated serum bilirubin [3]. This is a genotype-conditioned caution in an approved cannabinoid label, the second alongside the CYP2C9 caution in the dronabinol labeling, but not a genotype-guided dose recommendation, naming a susceptible group without an adjustment or genotyping step, and not a convergence on a single named enzyme: the inhibited isoforms are UGT1A9 and UGT2B7, whereas bilirubin is glucuronidated principally by UGT1A1, which no source reports nabiximols to inhibit and CBD does not inhibit [5]. Genotype and stated inhibition may therefore converge at glucuronidation capacity rather than at a single demonstrated clearance step, unlike the CYP2C19-clobazam and CYP2C9-THC pairings, and are best read as a regulator-recognized, pathway-level caution. Such relationships exist for other substrates, notably mycophenolic acid, so the cannabinoid gap reflects a lack of dedicated study rather than a demonstrated absence of effect [86,87]; UGT1A9 remains a tractable candidate convergence node, yet no study identified in this review has examined CBD inhibition and UGT polymorphism together. In vitro work points to where such a study should look: cannabinoid inhibition of UGT2B7-mediated hydromorphone glucuronidation differed between the UGT2B7*2 variant and the wild-type enzyme [83], the expected signature of a genotype-by-inhibition convergence at a UGT node, though no human test of it was identified in this review.

### 5.4. Product-Specific Evidence Gaps: Nabilone and Nabiximols

Pharmacogenomic evidence for nabilone is very limited. It is extensively metabolized by multiple cytochrome P450 isoforms; its disposition is less well characterized than that of other agents; and its prescribing information contains no pharmacogenomic section [6]. A further obstacle is specific to this agent: the parent disappears rapidly from plasma, whereas total radioactivity persists much longer, and metabolites may accumulate above parent concentrations on repeated dosing, so Rubin and colleagues concluded that pharmacological effects may be exerted at least in part by active metabolites whose activity the labeling records as not established [6,88]. The parent-plus-active-metabolite form of the framework therefore cannot yet be operationalized for nabilone, though parent-drug exposure variability can be organized within it preliminarily; characterizing those metabolites is a prerequisite for any complete precision-dosing account, which is why nabilone does not appear in the pathway matrix. A recent therapeutic drug monitoring proposal reaches the same conclusion from the opposite direction, identifying only a single study relating nabilone concentration to effect [89]. The disposition data are correspondingly thin: eight male volunteers were given a single 2 mg oral dose of radiolabeled nabilone as a polyvinylpyrrolidone coprecipitate, not the marketed capsule, with a parent half-life of about 2 h against a total-radioactivity half-life of 20.6 h [88], for which the labeling reports approximately 35 h [6], and with the quantities recovered too small to permit identification of the metabolites concerned.

Because nabilone is a distinct synthetic analog, and CBD and THC differ in metabolism and receptor pharmacology, pharmacogenomic data should not be transferred unless explicitly identified as indirect and its basis stated. For nabiximols, the gap is narrower. Product-specific PK evidence is substantial: interaction studies with rifampicin, ketoconazole, omeprazole, and fluconazole; hepatic and renal impairment studies; a food effect; and exposure variability [3,38,62,90]. Two elements are missing. The first is pharmacogenomic: no genotype-stratified PK study identified in this review has assessed any analyte in the approved product. The second is analytical: for its four species (THC, 11-OH-THC, CBD, and 7-OH-CBD), there is neither a validated exposure–response relationship nor a scheme for combination, so active exposure is specifiable at the analyte level only (Section 3.1).

### 5.5. Membrane Transport

Membrane transport must be interpreted precisely, because four separable properties are involved: substrate status, inhibition, the compartment in which inhibition occurs, and genetic variation. The transporters concerned are P-glycoprotein (P-gp; encoded by ABCB1), the breast cancer resistance protein (BCRP), and BSEP. Substrate status is not inhibition: the abundant metabolite 7-COOH-CBD is a P-glycoprotein substrate, whereas labeling does not identify CBD itself as a substrate [5], although in silico docking has predicted that it could be [91]. In vitro, CBD inhibits P-glycoprotein-mediated transport at low-micromolar concentrations and is comparably potent to tariquidar in cultured brain cells [91,92]. The labeling nonetheless states that neither CBD nor 7-OH-CBD is anticipated to interact with P-glycoprotein, while noting that intestinal inhibition cannot be excluded and that in vivo data indicate an effect on intestinal efflux [5]. The positions are reconcilable with respect to concentration: steady-state plasma CBD at approved doses lies well below the inhibitory concentration, whereas luminal concentrations after an oral dose could plausibly exceed it, making an intestinal contribution more plausible than systemic inhibition.

The everolimus result is consistent with, but does not establish, that reading: exposure rose approximately 2.5-fold without a material change in half-life, consistent with a predominantly absorption-related mechanism, though transporter and mechanism remain unconfirmed, and no selective P-glycoprotein probe study for this dual P-glycoprotein/CYP3A4 substrate was identified in this review [37]. Separately, 7-COOH-CBD inhibits BCRP- and BSEP-mediated transport at clinically relevant concentrations, but neither is evidence of P-glycoprotein interaction. Intestinal P-glycoprotein inhibition is plausible and label-relevant, supporting advice that exposure to oral substrates (sirolimus, tacrolimus, digoxin) may increase, though this inference rests on concentration-based reasoning, not direct demonstration.

Transporter genotype is an exploratory extension only. ABCB1 is polymorphic, and P-glycoprotein function can also be modified pharmacologically, so the pathway formally satisfies the convergent-modifier definition used here, yet no study was identified that relates ABCB1 genotype to exposure to any approved cannabinoid. One study relates ABCB1 genotype to clinical response to nabiximols [80], but it measured no cannabinoid concentrations, so it addresses the response endpoint rather than exposure and cannot localize the effect to transport. Two further studies relate ABCB1 genotype to clinical endpoints rather than to exposure: in 600 patients treated with cannabis for chronic pain, ABCB1 was one of three polymorphic genes associated with the reduction in pain [93], and in a case–control comparison the C3435T CC genotype was over-represented among cannabis-dependent users [94]. Neither measured cannabinoid concentrations, so both address response or liability rather than exposure. Two small human studies do relate ABCB1 genotype to systemic cannabinoid exposure, but neither involved an approved medicine, and the two are not directly comparable: in 48 young adults smoking a single cannabis cigarette, carriers of the rs2235048 C allele had higher areas under the curve for 11-OH-THC and THC-COOH, with the association influenced by self-reported ethnicity [95]; in 39 heavy chronic users, carriers of the rs1045642 (C3435T) T allele had lower plasma Δ^9^-THC [96]. The analytes, exposure conditions, and variants differ, and the reported allele effects cannot be aligned without haplotype information, so the transporter hypothesis has indirect human support rather than none, but remains exploratory. It is not comparable in maturity to CYP2C19, CYP2C9, or CYP3A, and generates no prediction in Box 1.

### 5.6. Clinical Actionability and Phenoconversion

Genotype-determined activity and phenoconversion are distinct constructs and are treated separately here. Three quantities must be kept apart. Genotype-inferred enzyme function is the activity predicted from sequence before any drug is given; it is stable but predicts rather than determines a patient’s actual clearance. Actual functional activity is what occurs under current physiological and treatment conditions and may differ from the inferred value because of age, inflammation, hepatic disease, and comedication. Phenoconversion is the change from the first to the second produced by an extrinsic factor, most often an enzyme inhibitor or inducer; it leaves the genotype unchanged and is in principle reversible, though the functional effect need not end the moment the factor is withdrawn. A phenoconverted normal metabolizer and a genetically reduced-function carrier may transiently display similar activity while differing in baseline, in reversibility, and in the fold change the same inhibitor may produce in each. Genotype and phenoconversion are therefore separate inputs to one clearance step, not alternative descriptions of a single quantity. It follows that demonstrating phenoconversion does not demonstrate a genotype-by-treatment interaction: an inhibitor may shift every stratum without the shift differing by genotype, which is the multiplicative case in the following paragraphs. Any genotype-based prediction is provisional for this reason. Genotype-inferred metabolizer status remains the reference classification, whereas actual functional activity may depart from it under concurrent enzyme inhibitors or inducers, systemic inflammation, hepatic disease, and the polypharmacy typical of the populations using these medicines [97,98,99]. Genotype and comedication may therefore jointly determine absolute exposure: a reduced-function metabolizer also given an inhibitor of the same enzyme starts from a different baseline than a normal metabolizer given the same inhibitor. However, it should not be assumed that a reduced-function genotype plus enzyme inhibition necessarily produces a compounded or supra-additive increase. The joint effect may be additive, subadditive, or greater than additive, depending on the affected enzyme’s residual activity, the available parallel clearance pathways, and the inhibitor’s potency and mechanism.

Absolute exposure and inhibitor-related fold change are distinct quantities and must not be used interchangeably when predicting risk or designing studies. What the framework predicts is the joint determination of absolute exposure, not any arithmetic relationship between the two effectors. The distinction is empirically decidable: a multiplicative and a residual-enzyme model agree that absolute exposure is highest in reduced-function carriers but disagree on the inhibitor-related fold change, which is uniform across phenotype groups under the first and smaller in reduced-function carriers under the second. Expressed as a genotype-by-treatment interaction on the fold-change scale, the multiplicative model therefore corresponds to a null interaction and the residual-enzyme model to a smaller fold change in reduced-function carriers (Box 1, P1). Only a genotype-stratified before-and-after study distinguishes them; multiplying point estimates from different populations yields pseudo-precision. That joint determination has not been measured in any approved-product study identified in this review. The closest clinical illustration identified is a single case report: in a 78-year-old CYP2C19 intermediate metabolizer (*1/*2) stable on sertraline 50 mg daily for twenty years, addition of an over-the-counter CBD supplement was followed by progressive hyponatremia to a serum sodium of 120 mmol/L with cognitive impairment; cognition improved, and serum sodium rose to 132 mmol/L after withdrawal of the CBD supplement and subsequently of sertraline [100]. That report is hypothesis-generating only: no sertraline or CBD concentrations were measured, the product was unregulated and of unverified content, concomitant omeprazole is itself a CYP2C19 inhibitor, and sertraline-associated hyponatremia is not regarded as dose-dependent. It is consistent with the convergence the framework hypothesizes, without quantifying either input.

That divergence has a straightforward biochemical basis. In the static mechanistic model used in regulatory interaction assessment, the exposure ratio produced by a reversible inhibitor is governed by the unbound inhibitor concentration relative to the inhibition constant and by *f*_m_, the fraction of systemic clearance carried by the inhibited enzyme; as *f*_m_ falls, the achievable fold change contracts toward unity regardless of inhibitor potency [101]. Genotype does not enter through the inhibitor term. It enters through *f*_m_: in a reduced-function carrier, the variant enzyme carries a smaller share of an already smaller total clearance, so the same degree of enzyme inhibition produces a smaller fractional change in exposure, even though absolute exposure in that stratum is higher. The multiplicative model is the special case in which *f*_m_ is treated as genotype-independent. Framing the contrast in these terms makes the core genotype-by-treatment predictions in Box 1 statements about pathway contribution (*f*_m_) rather than arithmetic, and identifies the parameters that a stratified design must estimate: the unbound inhibitor concentration at the enzyme and *f*_m_ at the convergent node within each genotype stratum.

For the present, none of this supports routine genotype-guided prescribing. Although the CYP2C9–dronabinol pair is recognized in pharmacogenomic resources [18], and the dronabinol label recommends monitoring where CYP2C9 function is diminished, no Clinical Pharmacogenetics Implementation Consortium [81], Dutch Pharmacogenetics Working Group [102], or comparable guideline provides a genotype-based dose adjustment for any approved cannabinoid. Both groups issue such guidance for other CYP2C9 and CYP2C19 substrates, including nonsteroidal anti-inflammatory drugs and proton-pump inhibitors [103,104], making the cannabinoid absence a gap rather than an artifact of institutional scope. The decisive gap is that this review found no evidence that genotype-guided dosing improves efficacy or reduces harm. The tractable near-term direction is therefore not genotype alone but phenotype-guided monitoring that integrates genotype with measured parent and active-metabolite exposures, hepatic function, comedication, and adverse-event surveillance, analogous to model-informed precision dosing elsewhere [105]. Genotype thus sets a baseline that comedication perturbs; the next section turns to that perturbation, where the evidence is firmer.

## 6. Drug–Drug Interactions as Modifiers of Exposure

The preceding section treated host genotype as one exposure determinant; this one examines comedication as another. The victim and perpetrator endpoints are not interchangeable, and the best-quantified pairing below turns on this distinction. Both matter because these medicines serve polymedicated populations, developmental epilepsies, multiple sclerosis, and chemotherapy-induced nausea and vomiting, in which several substrates of the same CYP and glucuronidation pathways coexist. The evidence for interactions is comparatively mature and, for several pairs, well quantified [106]. Interactions are ordered below by the strength of their evidence, characterized as defined in Section 2 and recorded in Table 1. Where a pathway carries a genotype axis, that convergence is noted at the point the interaction is introduced.

### 6.1. Cannabinoid as a Perpetrator

#### 6.1.1. Quantified Interactions with Controlled Human Evidence

The CBD–clobazam interaction is the best-quantified of any approved cannabinoid, a perpetrator-branch example; the changed exposure is the substrate’s active metabolite, not the cannabinoid. It is also the framework’s strongest candidate convergence node, because the enzyme CBD inhibits, CYP2C19, is the same polymorphic enzyme that sets baseline N-desmethylclobazam clearance. In a twelve-participant arm of an open-label, fixed-sequence phase 1 trial, CBD 750 mg twice daily increased the active metabolite N-desmethylclobazam approximately 3.4-fold, while parent clobazam increased only 1.2-fold [31]; in children, the estimate was larger: N-desmethylclobazam increased approximately 500% despite clobazam dose reductions in most subjects, with a confidence interval that included the controlled adult estimate [30]. In a therapeutic drug monitoring cohort of 52 patients with refractory epilepsy, 24 of whom were on clobazam, the mean N-desmethylclobazam-to-clobazam ratio increased by 140% (from 7.29 to 17.5; *p* < 0.05), though variably [7]. CYP2C19 inhibition explains the selectivity: CBD inhibits the enzyme that clears N-desmethylclobazam, not the one that generates it, so the parent moves little and inconsistently, falling significantly in one open-label cohort as the CBD dose rose [74]. The magnitude was largely predictable: in vitro data supported a static projection of an approximately 3.1-fold increase in a CYP2C19 substrate [57], agreeing in direction and order of magnitude, though not validated. The adult estimate has since been reproduced independently under controlled conditions: in a phase 2, randomized, double-blind, placebo-controlled trial, 20 patients receiving stable clobazam were assigned to CBD (n = 16) or placebo (n = 4), and the pharmacokinetic analyses included 10 CBD-treated and three placebo-treated patients. Within the CBD group, day 33-to-day 1 geometric mean ratios for N-desmethylclobazam were 2.6 (90% CI 2.0–3.6) for AUC and 2.2 (90% CI 1.4–3.5) for maximum concentration, while clobazam exposure itself was unchanged (AUC ratio 1.1, maximum concentration ratio 1.0), and placebo had no effect on either analyte [32]. This is one of the few relationships reviewed here supported by two independent controlled human estimates with formal confidence intervals.

The clinical consequence is double-edged. Because N-desmethylclobazam is pharmacologically active, its accumulation can contribute to antiseizure efficacy and to somnolence and sedation; the sedative effect reflects changes in exposure, not co-prescription alone: in a study, sedation was significantly more frequent with higher serum N-desmethylclobazam concentrations (*p* = 0.02) [74]. It may also have contributed to responses in clobazam-treated subgroups of the pivotal trials, without excluding CBD’s independent antiseizure effects [107]. The monitoring implication, namely anticipating higher active-metabolite exposure, watching for sedation, and considering a clobazam dose reduction, turns on exposure, not co-prescription itself.

Among non-antiseizure medicines, the best-substantiated emerging interaction is tacrolimus, a narrow-therapeutic-index immunosuppressant. A prespecified interim analysis of the same trial (Table 1, footnote (c)), in the 12 participants who had completed all three periods, found that CBD titrated to 5 mg/kg twice daily increased the maximum concentration of tacrolimus by approximately 4.2-fold and the AUC by approximately 3.1-fold (both *p* < 0.0001), with unchanged half-life; the mechanism remains unclear [33]. This is consistent with a single case report of an approximately threefold increase in dose-normalized tacrolimus in a participant on pharmaceutical CBD at 20 to 25 mg/kg/day (reported as 2000 to 2900 mg/day), with rising serum creatinine and an approximately tenfold dose reduction [34]. The controlled magnitude establishes clinical relevance, and the recommendation is not inferential: the tacrolimus labeling carries a cannabidiol-specific warning directing close monitoring of tacrolimus blood levels and signs of toxicity, with a tacrolimus dose reduction to be considered as needed [35]. The CBD labeling independently lists tacrolimus as an oral P-glycoprotein substrate for which therapeutic drug monitoring and dose reduction should be considered [5]. This is the only relationship reviewed here that carries a label-level action in both the perpetrator’s and the victim’s labeling.

The mechanism is less well defined than the magnitude. CYP3A inhibition is plausible: CBD and 7-OH-CBD inhibit tacrolimus metabolism in vitro, and two independent lines indicate greater in vitro inhibition of CYP3A5 than of CYP3A4 [108,109]. These findings support involvement of both isoforms without quantifying their relative contributions in vivo, and the reported inhibition constants cannot be scaled against total plasma CBD, which ignores protein binding; 7-OH-CBD inhibited both isoforms similarly [33]. CYP3A5 is expressed on both sides, since expresser status also sets baseline tacrolimus clearance; the Clinical Pharmacogenetics Implementation Consortium recommends a 1.5- to 2-fold higher starting dose in expressers with known genotype, while declining to recommend testing [110]. That convergence grounds prediction P3 in Box 1 and is untested. Ancestry is not a substitute for genotype [110]. The interaction should not be described as exclusively or predominantly CYP3A5-mediated: the investigators state it may arise from CYP3A4, CYP3A5, hepatic or intestinal metabolism, or intestinal transporters, and that this remains to be determined [33,108].

The everolimus interaction likewise rests on a single dedicated controlled PK study: steady-state CBD at 12.5 mg/kg twice daily increased the maximum concentration and AUC of a single 5 mg everolimus dose by approximately 2.5-fold, prompting blood concentration monitoring and dose reduction [37]. A case report of tuberous sclerosis complex shows the same effect and its instability, with troughs roughly tripling after CBD initiation and rising further despite dose reduction [36]; the labeling advises reducing oral everolimus with monitoring and using a lower starting dose when adding everolimus to established CBD [5]. The mechanism may be predominantly absorption-related rather than metabolic, though this is not established: everolimus half-life was not prolonged at steady-state CBD (mean 40.0 h alone versus 33.2 h with CBD; n = 16) [37], and CBD’s failure to raise midazolam exposure argues against a substantial hepatic CYP3A4 contribution [5]. This is the closest approach here to a possibly transporter- rather than enzyme-mediated change, aligning with the labeled intestinal P-glycoprotein effect and the flagged oral P-glycoprotein substrates tacrolimus, sirolimus, and digoxin, though both absorption and P-glycoprotein inhibition remain inferred. Its actionability matches that of tacrolimus, but the two should not be merged into a single CYP3A interaction.

#### 6.1.2. Limited, Indirect, and Directional Signals

The remaining perpetrator signals rest on more limited or indirect evidence, and the four evidence levels must be kept apart. In vitro, CBD inhibits CYP2C19 [57], the CYP3A isoforms [109], and several UGTs, most potently UGT1A9 [82], while circulating THC metabolites competitively inhibit CYP2B6, CYP2C9, and CYP2D6 [111]. The CBD labeling is pathway-specific rather than uniform, advising dose modification for sensitive CYP1A2, CYP2B6, CYP2C8, CYP2C19, UGT1A9, and oral P-glycoprotein substrates while reporting negative dedicated studies for CYP2C9 and CYP3A4 (tolbutamide and midazolam unchanged) [5]. In vitro inhibition therefore does not entail a labeled action, and the perpetrator role is neither uniform across enzymes nor exclusively inhibitory. Nor is the branch confined to CBD, though the evidence tier differs sharply: the nabiximols labeling reports only that clinical concentrations could induce CYP1A2 and CYP2B6 at the mRNA level in vitro, with no human substrate exposure measured and no midazolam effect observed [3], a mechanistic possibility running in the opposite direction, not a labeled action. Separately, the CBD labeling reports no UGT1A6 inhibition in vitro [5]. Recombinant-enzyme work reports the opposite, with a binding-corrected IC_50_ of 0.40 µM [82]. The discordance therefore lies between in vitro datasets rather than between label and bench. Dedicated human glucuronidation probe studies extend the perpetrator evidence beyond these label statements (Section 5.3), a distinction that should not be confused with the pharmacogenomic gap.

Direct human probe studies are of moderate magnitude. Human studies of the approved product exist for only a subset of these pathways and do not scale with in vitro potency: CBD nearly doubled the caffeine AUC, consistent with inhibition of CYP1A2, whereas bupropion exposure decreased by approximately 20%, while exposure to its CYP2B6-formed active metabolite, hydroxybupropion, was unchanged [5,112]. The latter pattern cannot be interpreted as simple CYP2B6 inhibition and is consistent with labeling indicating that CBD may both inhibit and induce CYP2B6 at clinically relevant concentrations. A high-dose probe-cocktail study of a CBD-dominant extract (640 mg CBD with 20 mg Δ^9^-THC) gave an in vivo inhibition rank order of CYP2C19 > CYP2C9 > CYP3A > CYP1A2 with no CYP2D6 effect, while the Δ^9^-THC-only extract inhibited none [44]. The strongest demonstrated perpetrator effects at clinically evaluated doses are therefore attributable to CBD, though from a high-dose extract rather than an approved medicine; the extract increased midazolam exposure, whereas the approved product did not [5,44]. Clinical risk is concentrated wherever a narrow-therapeutic-index substrate shares a pathway with human PK evidence of a CBD effect. Two subsequent human studies extend this pattern in opposite directions. A regulatory-authored trial in healthy adults increased citalopram exposure by approximately 43% while leaving morphine exposure essentially unchanged [113], and a crossover study found that CBD at 30 mg, far below antiseizure doses, increased amitriptyline exposure by approximately 13% while leaving tramadol unchanged [114].

A directional case: prodrug substrates. A final perpetrator-branch feature is directional rather than magnitude-related, made explicit in the labeling: for most CYP2C19 substrates, CBD inhibition raises exposure and a dose reduction is considered; where activity resides in a CYP2C19-generated metabolite, the same inhibition lowers active-metabolite concentrations and may reduce efficacy, so the labeling advises considering a dosage increase in such substrates, as clinically appropriate, naming clopidogrel [5]. One enzyme thus carries two opposite recommendations depending on where activity resides, the parent-to-metabolite logic of Box 1. The pharmacogenomic layer is predicted to converge here: a patient with CYP2C19 intermediate- or poor-metabolizer status receiving clopidogrel and CBD faces two constraints on a single bioactivation reaction, since reduced CYP2C19 function alone lowers active metabolite formation and platelet inhibition, raises adverse cardiovascular event risk, and underlies an established genotype-guided antiplatelet recommendation [115]. The aggregate direction is not fixed by CYP2C19 alone, since clopidogrel bioactivation also involves CYP1A2, which the labeling describes as weakly inhibited by cannabidiol, and CYP2B6, where the labeling describes cannabidiol as both an inducer and an inhibitor, and it is distinct from UGT2B7-mediated glucuronidation of clopidogrel carboxylic acid. No study was identified that measured clopidogrel active metabolite concentrations or platelet reactivity under CBD in any genotype stratum, so the pairing is a research-only hypothesis and no prediction is framed for it in Box 1.

Case-based and indirect signals. The evidence for warfarin is weaker and largely case-based. International normalized ratio elevations, with occasional bleeding, have been described mainly with cannabis or non-approved products, frequently at high doses [43,116], and two low-certainty cases involve the FDA-approved product, both requiring warfarin dose reduction and one with alternative explanations identified [41,42]. The mechanism should not be assigned: cannabinoids inhibit CYP2C9-mediated S-warfarin 7-hydroxylation in vitro [116], but this is at odds with the negative tolbutamide probe study and with labeling that carries no CYP2C9 substrate recommendation. Warfarin’s racemate nature prevents assigning an enantiomer-selective effect without stereoselective concentrations, which no report identified in this review has provided. The pathway is therefore unassigned; no predictable magnitude has been established for the approved product, and the evidence supports vigilance over the international normalized ratio at initiation and during dose changes rather than an empirical dose reduction; systematic syntheses consistently flag these narrow-therapeutic-index interactions as among the greatest concerns [20,117].

### 6.2. Cannabinoid as a Victim

External inducers and inhibitors. Because CBD is cleared by CYP3A4 and CYP2C19, strong inducers lower exposure to both the parent and active metabolite: in the dedicated labeling study, rifampin reduced CBD AUC by 32% and the active metabolite 7-OH-CBD substantially more, by 63% [5], a direction reproduced in physiologically based modeling [118]. The prescribing information recommends considering an Epidiolex dose increase, up to twofold, with a strong CYP3A4 or CYP2C19 inducer, guided by clinical response rather than applied automatically [5]. The same mechanistic direction is plausible for other strong inducers, including some enzyme-inducing antiseizure medications, but this extension rests on shared pathway assignment rather than measured CBD exposure and is an inference; no dedicated study identified in this review has quantified the effect of such medications on CBD or 7-OH-CBD concentrations. This interaction is easy to overlook: its consequence is lost efficacy, not toxicity; reduced exposure produces no adverse event and, in epilepsy, may present as loss of seizure control attributed to the disease. Induction thus deserves the same counseling attention as inhibition, and unexplained loss of response in a stabilized patient should prompt review of concomitant inducers.

By contrast, in the same labeling, strong inhibitors of CBD’s clearance enzymes altered exposure marginally: itraconazole by less than 10%, and fluconazole raised CBD AUC by 22% while lowering 7-OH-CBD by 28%; the label judges these changes not clinically meaningful [5]. That asymmetry is not general: in nabiximols, CYP3A inhibition produced larger changes than CBD-clearance inhibition at Epidiolex doses [38]. The findings need not conflict: the studies differed in CBD dose, formulation, route, THC co-exposure, and conditions, none isolated. Across both, analyte specificity holds: fluconazole raised parent CBD while lowering 7-OH-CBD, and in nabiximols the active THC metabolite rose proportionally more than the parent; parent concentration is a poor proxy for the pharmacologically relevant quantity.

Nabiximols is the only approved THC-containing medicine for which victim-directed interaction evidence was generated with the product itself. In the Phase I study, enzyme induction lowered, and CYP3A inhibition raised all three analytes, with the active metabolite moving proportionally more than the parent in every arm, while omeprazole produced no significant change [38]. The labeling adds a CYP2C9 fluconazole arm in which THC AUC increased by 32% and 11-OH-THC AUC increased by approximately 2.5-fold [3]. Expressed as the ratio of the reported AUC fold-changes, the fold-increase in the active metabolite was approximately 1.9 times that in the parent (approximately 2.5-fold versus approximately 1.32-fold), comparing group-mean exposures within a single study arm rather than a measured individual-subject ratio. The analyte-specific point holds within a single labeled product.

The changes are consistent with CYP3A involvement in the disposition of both the parent cannabinoids and 11-OH-THC, though rifampicin is not CYP3A-selective, and the study neither isolated all contributing pathways nor separated metabolite formation from elimination. The study does not establish genotype–interaction convergence; therefore, the interaction arm is populated for nabiximols, whereas the pharmacogenomic arm is not.

Two features bound what the study can support. From the omeprazole arm, investigators concluded the analytes are CYP3A4, not CYP2C19, substrates, a conclusion stronger than the data allow: no sample-size calculation was reported, and although the group-mean changes with omeprazole were not statistically significant, the 90% confidence intervals for THC and CBD were too wide to fall within the pre-specified 0.80 to 1.25 no-interaction range, so the study does not establish the absence of a CYP2C19 contribution. The design gave a four-spray bolus, not divided across the day, in healthy men only, with substantial variability, and it was manufacturer-sponsored; it establishes the direction of CYP3A effects more securely than their magnitude during chronic titrated use.

Food and physiological modifiers: exposure is also shaped by non-comedication factors, label-quantified for these products rather than inferred. Of these, the severe renal impairment result splits parent from metabolite as sharply as any inhibitor.

Intra-product interaction and indirect evidence. These data are not equivalent to evidence from high-dose extracts. The nearest indirect comparator, in which 640 mg oral CBD in a CBD-dominant extract raised THC exposure by roughly 160% in healthy adults [44], address a different question: an intra-product CBD–THC interaction, not an external perpetrator. The nabiximols study is direct but small and single-dose; the extract study produced a larger effect but is indirect. Directness and dose relevance coincide in neither, and neither supplies what the other lacks.

## 7. The Convergent Exposure-Modifier Framework

The framework brings the two modifier classes together and rests on three propositions. First, the two modifier classes are pathway- and product-specific rather than uniform: the clearance nodes carrying genetic variation differ, though not completely, between CBD and the THC-based medicines, and the interactions acting on them differ accordingly. Second, their joint effect may alter exposure to the cannabinoid and its active metabolites, or to a co-administered substrate, so the relevant exposure endpoint is set by the victim’s or perpetrator’s direction rather than fixed in advance and may influence efficacy or toxicity according to which end of the exposure distribution a patient occupies. Third, post-approval safety signals prioritize which outcomes to test prospectively but do not, by themselves, validate convergence; that ordering is an author-generated research priority: it reflects an interpretation of the available evidence rather than a ranking established by the pharmacovigilance analysis itself, and is set out with its basis in Section 4.4. The framework therefore predicts heterogeneity across genotype and comedication combinations rather than a universal direction or magnitude and is offered as hypothesis-generating: support for the component relationships varies by pathway and product, and the central integrative claim that the modifiers converge has not been tested directly in any approved-product study identified in this review. The qualification applies at the node level. None of the three core predictions rests on evidence from unregulated products, high-dose extracts, or general cannabis exposure: P1 and P3 derive from studies of the approved cannabidiol product, and P2 from the dronabinol label together with a genotype study in oral Δ^9^-THC of unspecified formulation, an extrapolation across formulations that Section 5.1 states and Box 1 records as a boundary on the prediction. Evidence from non-approved or incompletely specified formulations contributes mechanistic context and may inform research hypotheses but does not establish the magnitude or clinical consequences of an interaction in an approved product. Where such evidence contributes to a prediction or a precaution, as it does for P2 and for the warfarin vigilance in Section 8.1, its indirectness and the assumptions required for extrapolation are stated at that point. Indirectness is a matter of product identity and formulation specification rather than of dose alone: a high dose of the approved product limits the applicability of a finding to labeled dosing but does not by itself make it indirect.

### 7.1. Genotype and Comedication as Convergent Modifiers

Inherited enzyme variation and concurrent medication may affect shared clearance pathways, providing a mechanistic rationale for investigating their joint effects: the pathways implicated in cannabinoid disposition, CYP2C9 and CYP3A4 for oral THC and dronabinol, and CYP2C19, CYP3A4, and UGT enzymes for CBD, are the same pathways at which the interactions reviewed above operate. Cannabinoid pharmacogenomic evidence is currently strongest for CYP2C9 and preliminary or absent for the others. Where a single pathway is both genetically variable and a target of interaction, a joint effect is mechanistically plausible, and the framework’s central, falsifiable prediction follows: pharmacologically relevant exposure, whether of the cannabinoid and its active metabolites or of a co-administered substrate and its active metabolite, and any exposure-mediated effect, should vary systematically across strata defined jointly by genotype and comedication, and those strata should explain exposure better than either modifier alone. These are two claims rather than one: that the treatment-associated change in exposure differs by genotype, which is effect modification and is tested by P1 to P3, and that the two modifiers together predict exposure better than either alone, which is incremental explanatory value and is tested by P5. Two independent main effects could satisfy the second without the first. The prediction concerns heterogeneity across strata, not enrichment in any particular one, since which stratum carries the highest exposure depends on pathway, product, and residual enzyme activity. That drug–drug–gene interactions matter in general is established elsewhere: potent CYP2D6 inhibition reduces endoxifen in extensive metabolizers to less than a third of its uninhibited concentration, phenocopying a poor-metabolizer genotype [119], an analogy that demonstrates the phenomenon without validating it for cannabinoids. The convergence is accordingly biologically plausible but unvalidated, and is presented here as a construct to be tested rather than as an established property of these medicines.

The logic is most concrete where the evidence for each link is strongest: the CYP2C19–clobazam node. CYP2C19 genotype sets the baseline clearance of the active metabolite N-desmethylclobazam, and CBD inhibits that same enzyme, so a reduced-function genotype and CBD-mediated inhibition act on the identical clearance step from two directions, one inherited and one pharmacological, with independent magnitude estimates for both. What the framework adds is a prediction neither component supplies: that CYP2C19 phenotype modifies the change in N-desmethylclobazam exposure a patient undergoes when CBD is introduced, and hence the patient’s position within the population exposure distribution. The same node also carries the framework’s sharpest methodological test, since absolute exposure and inhibitor-related fold change need not move together, and their relationship discriminates the two candidate models (Section 5.6). The example links the safety, pharmacogenomic, and interaction evidence into one testable chain, without implying any particular arithmetic combination.

Three core predictions instantiate this logic, one in each branch where evidence for both modifiers is strongest and not equally mature. The victim-branch prediction concerns CYP2C9 and dronabinol, where the genotype signal is quantified but derived from oral THC of unspecified formulation. The two perpetrator-branch predictions concern CYP2C19 with clobazam, the strongest node because both modifiers act on a single named enzyme, and CYP3A5 with tacrolimus, where the interaction is established but the contributing enzymes and compartments have not yet been identified. Each is stated formally, with its endpoint and its falsification criterion, in Box 1; the corresponding study designs are in Section 8.2. All three are prospective in their entirety. Each is assembled from components observed separately, and no approved-product study identified in this review directly tested the specified genotype-by-treatment exposure effect for any of them; the differences in maturity just described concern the quality of those components, not any partial validation of the predictions themselves. What makes the framework falsifiable rather than descriptive is that one design settles them: genotyping before an interacting drug is introduced, with exposure measured analyte by analyte, can establish whether the two modifiers together explain exposure better than either alone, and equally can show that they do not.

### 7.2. Operationalizing the Framework: Evidence Map, Matrix, and Schematic

Claims are separated into four categories, which cut across the artifacts rather than being assigned to them whole. Established evidence is a relationship adequately supported by the studies cited for it, judged on design, replication, precision, and the limitations recorded beside it. Product directness is a separate axis: a finding measured in an approved product may still be weakly supported, and a well-designed study in a closely related formulation is not thereby direct evidence for the approved product. Both axes are reported for every row of Table 1A. Plausible mechanistic convergence is where a genetically variable pathway and an observed interaction meet, carried by the convergence column of Table 2 and drawn in Figure 1. Untested hypotheses are the status of the predictions in Box 1 and of every row of Table 1B, none of which has been tested in an approved product. Section 8.1 and Table 3 present the clinical implications, with each measure labeled by whether the labeling, a controlled study, or the author’s inference supports it. Table 1 and Table 2 therefore hold rows in more than one category, which is why each row states its own evidence type. The categories are not interchangeable, and none of these artifacts is a validated dosing instrument. Section 8 translates the framework into research design, monitoring, and the interpretation of existing safety data.

## 8. Clinical Implications and a Precision-Dosing Research Agenda

### 8.1. What Clinicians Can Do Now

The framework’s immediate value is anticipatory, not prescriptive: it selects no doses but flags where monitoring should intensify. Each measure below is labeled by its support, using the actionability categories defined in Section 2 and applied in Table 2 and Table 3. With prescription CBD plus valproate, and less so clobazam, monitor transaminases; although not explained by CBD exposure, the implication is unchanged. Label-supported. When CBD is added to clobazam, anticipate increased levels of the active metabolite, N-desmethylclobazam, and monitor for somnolence and sedation [5,31]. Controlled-PK-supported and label-reinforced. Consider clobazam dose reduction if clobazam-related adverse reactions, particularly somnolence or sedation, occur; a uniform reduction at initiation is not indicated. Label-supported. Where N-desmethylclobazam concentrations are available, they may provide additional exposure information to inform that decision. Exposure-informed and author-generated: the metabolite rise is quantified, but no concentration threshold for dose adjustment has been established.

Before starting CBD, review co-prescribed narrow therapeutic index substrates. Tacrolimus carries the strongest regulatory standing of any interaction reviewed here: its labeling contains a cannabidiol-specific warning directing close monitoring of blood levels and for signs of toxicity, with a dose reduction to be considered as needed [35], and the CBD labeling names it among oral P-glycoprotein substrates warranting therapeutic drug monitoring and dose reduction [5]. Everolimus is supported by a controlled PK study and by the CBD labeling, which specifies a lower starting dose in the reverse sequence, with everolimus added to established CBD [5,37]. Warfarin is supported only by case reports drawn largely from unregulated or high-dose products [20,43]. For tacrolimus and everolimus, arrange early, frequent therapeutic drug monitoring after CBD initiation; for warfarin, monitor the international normalized ratio at initiation and during dose changes. Also cover sensitive UGT1A9 substrates such as mycophenolic acid, whose exposure CBD raised in a dedicated human study and for which the labeling advises considering a dose reduction, and oral P-glycoprotein substrates such as digoxin, sirolimus, and tacrolimus, for which the labeling advises considering therapeutic drug monitoring and a dose reduction on account of intestinal inhibition [5]. The tacrolimus, everolimus, UGT1A9, and P-glycoprotein actions are label-supported; the warfarin action is author-only vigilance.

For the THC-based agents, the dronabinol label advises caution where CYP2C9 activity is diminished, consistent with the higher oral THC exposure in CYP2C9*3 carriers [28,29]. That caution is label-supported; routine genotyping to guide dosing remains research-only. Caution in older, frail, or fall-prone patients on THC-based agents alongside sedatives, antihypertensives, or opioids is author-generated, resting on class-level signals rather than on measured exposure [8,75].

Nabiximols carries product-specific label-supported actions, none genotype-based: administration should be standardized for food; the product is not advised in moderate or severe hepatic impairment; lower daily doses may be needed in moderate or severe renal impairment, in which the active metabolites rise several times more than the parents; and a new titration may be required when a strong CYP3A4 inhibitor or inducer is started or stopped, with care alongside potent CYP2C9 inhibitors [3]. No genotype-stratified pharmacokinetic evidence was identified for this product, so no genotype-conditioned action follows. Preliminary therapeutic reference ranges have been proposed for Δ^9^-THC in nabiximols and dronabinol, and, separately, for nabilone itself, since nabilone is not converted to Δ^9^-THC [89]. These provide a concentration anchor for monitoring in the absence of genotype-specific action, but they are proposals rather than validated exposure–response targets, and the nabilone range rests on a single concentration-effect study. None addresses active-metabolite exposure, which for nabilone remains uncharacterized.

Finally, when strong CYP3A4 or CYP2C19 inducers, such as rifampin, are co-administered with CBD, watch for loss of seizure control rather than overt toxicity, since reduced CBD exposure may present as apparent non-response [5]; the extension to enzyme-inducing antiseizure medicines remains unquantified and is author-generated. These points, summarized in Table 3, are monitoring and risk-management implications, not validated precision-dosing rules.

### 8.2. Precision-Dosing Research Agenda

The framework’s forward value is the studies it prioritizes. Three designs would test its core claims directly, forming the first group in descending order of maturity; this ordering, like the prioritization in Section 4.4, is author-generated. The CYP2C19–CBD–clobazam node is the strongest: both modifiers are quantified at one named enzyme, and the interaction and the genotype gradient are each independently established. A CYP2C9 inhibitor study in an oral THC product rests on a comparable genotype signal but carries unresolved formulation and probe selectivity. The CYP3A5–CBD–tacrolimus node is the most exploratory mechanistically, with its contributing enzymes and compartments undetermined, though clinically the most actionable. The clobazam and dronabinol designs are prospective, genotype-stratified studies following genotype-guided precedents [121]; the tacrolimus design is observational.

First, genotype-before-interaction studies in patients with epilepsy would test P1. Of the two designs of unequal priority, the inhibition design is the primary validation study, in which CBD is added to an established clobazam regimen. The induction design is a lower-priority observational alternative: strong inducers act on multiple pathways simultaneously and could not ethically be introduced solely for study purposes, so it depends on patients already receiving one. Recruitment must be enriched for poor metabolizers, whose frequency in unselected epilepsy cohorts would otherwise leave the interaction term underpowered, and the model comparison satisfying P5 should be prespecified and validated out-of-sample [98,99,122].

Second, CBD-initiation therapeutic drug monitoring cohorts in transplant recipients would measure co-substrate exposure before and serially after CBD has started. Tacrolimus and everolimus should be studied as parallel cohorts: the co-substrates differ in genotype axis (CYP3A5 established for tacrolimus, absent for everolimus), and neither mechanism is resolved, so pooling would obscure both. The primary endpoint should be dose-normalized exposure rather than raw trough, which the interaction-driven dose adjustments alter; secondary endpoints would be the proportion requiring a dose adjustment and the interval to the first out-of-range measurement.

The tacrolimus cohort would additionally record CYP3A5 expresser status, permitting the prespecified analysis of Box 1 P3, whereas no CYP3A5 hypothesis should be prespecified for the everolimus cohort, where no genotype axis is established [5,37]. Both are narrow-therapeutic-index substrates with label-level CBD interactions, so the cohorts are clinically justified, but their results should not be pooled on mechanistic grounds. Across all three Tier 1 designs, the same covariates must be recorded and modeled rather than assumed to be balanced: prandial state and meal composition, whose effect on CBD exposure equals or exceeds that of genotype (Section 3.1); formulation and route; hepatic function; inflammatory status, which itself phenoconverts; the full comedication list; and adherence.

Third, a prospective CYP2C9-stratified PK study of oral dronabinol would test P2. The original study already used genotype-enriched recruitment, but its principal contrast included only four CYP2C9*3/*3 homozygotes. Larger enriched strata are needed to improve precision and support a prespecified genotype-by-inhibitor analysis [28,29]. Because these homozygotes are uncommon, the design requires genotype pre-screening of a substantially larger pool, and this determines feasibility more than any other constraint. A second period would add fluconazole, accepting that no available inhibitor is CYP2C9-selective. All three designs share the measurement requirements. Each analyte must be reported separately and never summed, for the exposure–response reason given in Section 3.1. Sampling must be dense enough to estimate exposure, rather than relying on a single trough. When in vitro constants are to be compared with observed magnitudes, unbound rather than total plasma concentrations are required (Section 6.1).

Tier 2. Three further studies are high value, but either depend on Tier 1 or lack a characterized endpoint. A nabiximols multi-analyte genotype study would measure Δ^9^-THC, 11-OH-THC, CBD, and 7-OH-CBD against CYP2C19 and CYP2C9 phenotypes in a single cohort; the inhibition side has already been characterized, leaving only genotype stratification. It would establish whether a single polymorphic enzyme modifies exposure to both cannabinoids: a positive result would identify a polymorphic clearance node shared by both analytes, although a genotype main effect alone would not establish the genotype-by-comedication interaction the framework predicts; a null result would not establish the absence of a CYP2C19 contribution, and its interpretation would depend on the precision achieved, the representation of reduced-function genotypes, and the effects excluded by the confidence intervals, notwithstanding the in vitro evidence that it forms 11-OH-THC [79]. Second, the UGT and transporter extensions need a first study, not a definitive one, since no study identified in this review has linked UGT genotype to co-substrate exposure under cannabinoid inhibition or ABCB1 genotype to cannabinoid exposure in an approved medicine; the indirect human exposure evidence discussed in Section 5.5 does not resolve either approved-product gap. Third, characterizing the activity of nabilone’s long-lived metabolites is a prerequisite for applying the parent-plus-active-metabolite framework there.

Secondary priorities: Population-specific studies are needed in Gulf, Arab, Middle Eastern, African, and Asian populations, where CYP2C9 and CYP2C19 allele frequencies differ markedly from the predominantly European cohorts behind present estimates [123,124,125,126]; in one Saudi cohort, none carried two reduced-function CYP2C9 alleles [127], and no genotype-stratified cannabinoid PK study in Gulf or Middle Eastern populations was identified in this review. Life-stage studies need explicit adult-data extrapolation limits, prioritizing pediatric epilepsy on CBD and geriatric patients on THC medicines. Three mechanistic questions rank lower, each on a single in vitro observation: whether CBD-associated diarrhea reflects local intestinal exposure or formulation vehicle [40]; whether Δ^9^-THC and CBD influence phenytoin exposure oppositely [57,58]; a question to which two recent cases of reduced phenytoin clearance under CBD now add low-certainty human evidence [128]. Whether CYP2C19 contributes materially to Δ^9^-THC 11-hydroxylation in vivo no longer ranks here: the kinetics are now characterized in vitro [79], and the question is whether that contribution is detectable in patients with reduced CYP2C9 function.

The endpoint of this agenda is the development and prospective validation of a first cannabinoid pharmacogenomic and interaction-aware dosing framework that applies model-informed precision dosing to this class [129]. A defining requirement across all designs is genotype stratification and exposure measurement, which tests the convergence prediction directly rather than inferring it by aligning separate datasets.

## 9. Discussion

### 9.1. Principal Findings and Contribution

Four constraints bound what this section can conclude. No study-level risk-of-bias instrument, review-level methodological assessment, or certainty-of-evidence framework was applied, so no finding below carries a graded certainty. The synthesis is single-author, and retrieval was not enumerated, which limits confidence in its completeness. Several load-bearing observations are indirect, deriving from non-approved or incompletely specified formulations, which limits product-specific applicability. No approved-product study identified in this review measured a genotype-by-treatment exposure effect, so the joint-effect hypotheses are unvalidated and the central claim rests on aligned datasets rather than on any single study. Section 9.2 gives these, and the remainder, in full.

**Observed findings.** Read along the chain that the framework specifies: pathway, genotype, comedication, analyte-specific exposure, consequence. One genotype-linked relationship carries label-level standing and is quantified: reduced-function CYP2C9 with the higher oral Δ^9^-THC exposure it confers [28,29]. That genotype study used oral Δ^9^-THC of unspecified formulation, and its applicability to the approved dronabinol capsule is unconfirmed, making it partially direct rather than direct evidence for dronabinol (Section 2 and Section 5.1). The CYP2C19- and CYP3A-mediated determinants of cannabidiol exposure are likewise observed rather than inferred, resting on human genotype-stratified data, but those data are preliminary and paradoxical in direction, with a concordant external observation but no independent prospective replication, and they carry no clinical action. Exposure does not explain all real-world variability; the valproate hepatic signal remains the framework’s boundary condition.

**Mechanistic rationale.** The plausibility of convergence rests on genotype and comedication acting at the same clearance steps, not on any study that has measured them together. The glucuronidation-genotype caution in the nabiximols labeling is a regulatory caution rather than a measured genotype–exposure relationship [3] and is unquantified. Where a genetically variable enzyme is also the site of a quantified interaction, as at CYP2C19 for cannabidiol and clobazam, separately supported genotype and comedication effects make a joint effect plausible without demonstrating it.

**Untested hypotheses.** The joint genotype-by-treatment exposure effect has not been measured in any approved-product study identified in this review, so the three core predictions of Box 1 and every row of Table 1B remain untested. CBD is the most consequential interaction node, acting as a perpetrator at CYP2C19 and at narrow-therapeutic-index substrates including tacrolimus and everolimus, by mechanisms that appear to differ [37,108]; whether genotype modifies any of those interactions is untested. Several safety signals, including sedation, transaminase elevation, and falls, arise where exposure-modifying interactions are plausible, but their dependence on genotype or measured exposure has largely been untested.

What the framework adds. Organizing these studies around pharmacologically relevant exposures, with parent and active metabolites reported separately, makes them mutually informative rather than parallel across both branches. That it may be quantitatively tractable is suggested at the CYP2C19 node, where an in vitro-derived projection anticipated, in direction and order of magnitude, the interaction later measured in a controlled study (Section 6.1). A single retrospective concordance is not validation, but it identifies where prospective quantitative prediction should first be attempted. One qualification is owed to the regulatory literature: the clobazam labeling already recognizes both modifiers acting on a single clearance step [120], without equating them (Section 5.6), and the nabiximols labeling adds a genotype-conditioned bilirubin caution at the pathway level. No current label specifies what follows quantitatively when both are present in a single patient.

Where it is most testable. The framework is most readily tested where a quantified interaction, a relevant genotype axis, and a measurable exposure endpoint coincide, with parent and active metabolites assessed separately where relevant, since a genotype-stratified design requires it. Their coincidence indicates testability rather than support: it is met for CBD and, on one axis only, for each THC product, and the gradient is set out in Section 1.

**Clinical implications.** For research purposes, the framework translates a diffuse call for pharmacogenomic study into three specified designs with prespecified interaction terms. For clinical interpretation, two categories must be kept apart. Monitoring actions already supported by labeling or by controlled pharmacokinetic data rest on that evidence and remain unchanged by this review. What the framework adds beyond them is author-generated vigilance, which is research-oriented rather than a validated clinical management recommendation: it organizes existing advice around exposure and indicates in advance where exposure is likely to be atypical and which analyte would be informative. No untested prediction of the framework independently justifies a new clinical action, and none changes what is prescribed.

### 9.2. Limitations

This review has limitations that bound its conclusions. It is a narrative, integrative synthesis, with no quantitative pooling applied. The sole author performed searching, screening, full-text selection, and evidence characterization. The review was undertaken as a single-author work, with no second reviewer involved at any stage, so independent duplicate screening and characterization were not available to it; the resulting risk of selection and confirmation bias is present and cannot be quantified from the present record. The procedures adopted to constrain, though not to remove, the attendant risk are set out in Section 2 and are not restated here. None of them substitutes for independent duplicate screening, and the characterization of each relationship remains the author’s judgment; independent appraisal is warranted. The structured descriptive appraisal in Supplementary Table S2 does not substitute for a formal instrument. Retrieval was confined to English-language publications as an operational limit of a single-reader review, not on the premise that the relevant evidence is published in English; relevant non-English reports may therefore have been missed, and the negative claims in this review are bounded by English-language retrieval as well as by the searches described in Section 2. The supporting evidence is markedly unequal across the four products, so the framework’s apparent generality reflects the distribution of available evidence rather than uniform support. The pharmacology of the active metabolites is incompletely characterized, and no validated combined parent–active-metabolite exposure–response model is available to translate these profiles into individualized clinical effects or dosing decisions (see Section 3.1). Three consequences follow for interpretation. First, no statement here about parent–metabolite divergence is a statement about total active exposure; every such claim is analyte-level. Second, if 7-COOH-CBD contributes materially to therapeutic effect, interpretations resting on CBD and 7-OH-CBD alone would require reassessment; the measured exposure changes and the perpetrator-branch predictions would stand, but their translation into a combined pharmacological effect would be limited. Third, nabilone’s parent-drug activity is established whereas the identities and contributions of its potentially active metabolites are not, so its parent–active-metabolite profile cannot yet be specified, which is why nabilone is absent from the pathway matrix and generates no prediction.

Three further constraints concern the sources rather than the synthesis. The first is formulation. Oral cannabidiol solution, the oral dronabinol capsule and oromucosal nabiximols differ in absorption and in the fraction of dose subject to first-pass metabolism, and nabilone, although also given orally, is a distinct analog neither derived from nor converted to THC (Section 3.2). Whether a finding obtained in one product applies to another depends on the extent of first-pass metabolism, the swallowed fraction of an oromucosal dose, the dose, prandial state and, for nabiximols alone, co-administration of cannabidiol with THC. A modifier quantified in one product cannot therefore be assumed to transfer to another at the same magnitude, and whether the balance between parent and active metabolite shifts differently between products is a testable prediction rather than a demonstrated comparative finding (Section 3.2). Findings used to reason across products are identified as indirect at the point of use. The second and third concern the regulatory sources. Labeling differs across jurisdictions, so label-derived statements may not hold universally. Labels are also living documents requiring version control: during preparation of this review both the nabiximols Summary of Product Characteristics and the CBD labeling were revised, without alteration to the quantitative data relied on here [3,5,51]. Regulatory statements should therefore be cited to a dated revision and re-verified before submission. The predictions of Box 1 remain sensitive to uncertainty about the direction of active-metabolite formation (Section 3.1).

## 10. Conclusions

Approved cannabinoid medicines have generally been studied one determinant at a time. Read together through the common logic of pharmacologically relevant exposure, their pharmacogenomic, drug–drug interaction, and post-approval safety studies become mutually informative: the convergent exposure-modifier framework identifies where host genotype and comedication may converge on the exposure that matters in each direction, converts separate observations into falsifiable genotype-by-treatment hypotheses, and marks where monitoring is already supported by evidence. Monitoring actions supported by product labeling or controlled pharmacokinetic studies stand on that evidence; author-generated vigilance is identified separately as a hypothesis-generating, research-oriented consideration and does not constitute a validated clinical management recommendation (Section 8.1; Table 3). Its limits should be stated as plainly as its claims: it supports no genotype-guided dosing of any approved cannabinoid, defines no exposure threshold for action, and does not substitute for the prospective, genotype-stratified, exposure-measured studies that would establish either. The evidence supporting the proposed framework is predominantly cannabidiol-based. Its extension to dronabinol relies partly on CYP2C9 findings from oral THC of unspecified formulation; nabiximols lacks genotype-stratified pharmacokinetic evidence, and the contribution of nabilone metabolites to its effects remain unresolved. Accordingly, CEM is a hypothesis-generating conceptual model requiring prospective validation, not an established precision-dosing framework; its product-specific applicability remains to be established.

## Figures and Tables

**Table 2:** Pathway-based hypothesis and monitoring matrix: genotype and comedication as convergent modifiers of pharmacologically relevant exposure to approved cannabinoid medicines.

Pathway and Branch	Genotype Axis (Maturity)	Comedication Evidence (Type Stated per Row)	Convergence: PREDICTED, Untested	Monitoring Priority
CYP2C9 → oral THC/dronabinol (victim)	ESTABLISHED. Reduced-function CYP2C9 (*3) raises parent THC roughly threefold; the effect on 11-OH-THC is not established [28,29]	Measured in nabiximols; no inhibitor study in an oral product. In nabiximols, fluconazole raised THC exposure and raised 11-OH-THC exposure proportionally more (Table 1)	Genotype-by-inhibitor interaction in an oral product; direction and magnitude unknown for each of the three analytes (Box 1, P2)	Monitor psychoactive and sedative effects: label-supported, the dronabinol labeling advising monitoring for increased adverse reactions where CYP2C9 function is diminished. Cautious titration in that setting is author-generated, not a labeled rule
CYP2C19 → CBD + clobazam (perpetrator)	ESTABLISHED. Poor- and intermediate-metabolizer status raises baseline N-desmethylclobazam three- to fivefold and about twofold [120]	Measured in controlled human studies. CBD raises N-desmethylclobazam exposure while parent clobazam moves little; magnitudes in Table 1	CYP2C19 phenotype modifies the CBD-related change; the highest absolute exposure stratum need not show the largest fold-change (Box 1, P1)	Somnolence and sedation; consider clobazam dose reduction if clobazam-related adverse reactions occur; a raised metabolite concentration alone is not the labeled trigger. Controlled-PK-supported monitoring; the dose-reduction advice is label-supported
CYP2C19 → CBD + clopidogrel (perpetrator; prodrug direction)	ESTABLISHED in the clopidogrel field. Intermediate and poor metabolism reduces active-metabolite formation and platelet inhibition [115]	Inferred from enzyme inhibition plus labeling; not measured for this substrate: no study identified in this review measured a cannabinoid effect on clopidogrel active-metabolite exposure or on platelet reactivity. CBD is a moderate CYP2C19 inhibitor, and the labeling advises considering a dosage increase in CYP2C19 substrates whose efficacy depends on active metabolites, including clopidogrel, as clinically appropriate, the opposite in direction to its advice for other CYP2C19 substrates [5]	If the interaction occurs, both modifiers would constrain one bioactivation step in the same direction, and the lowest active-metabolite exposure would be expected in that stratum; the direction is not fixed by CYP2C19 alone, since bioactivation also involves CYP1A2, which CBD weakly inhibits, and CYP2B6, of which CBD is both an inducer and an inhibitor (Section 6.1.2). The established genotype relationship belongs to clopidogrel pharmacology; no direct clinical evidence identified in this review establishes the CBD-clopidogrel interaction or its genotype dependence, and no prediction is framed for this row in Box 1	Loss of antiplatelet efficacy rather than toxicity. This review proposes no additional monitoring or dosing recommendation beyond existing labeling; the pairing remains a research priority
CYP3A4/CYP3A5 (genotype axis; mediating pathway undetermined) → CBD + tacrolimus (perpetrator)	ESTABLISHED. CYP3A5 expresser status sets baseline tacrolimus clearance [110]	Measured in a controlled trial. CBD raises tacrolimus exposure severalfold; mechanism undetermined (Table 1)	Interaction magnitude may differ by expresser status; direction of the differential unknown (Box 1, P3)	Intensive therapeutic drug monitoring with individualized dose adjustment when CBD is started. Label-supported: the tacrolimus labeling carries a cannabidiol-specific warning advising close monitoring of blood levels and for signs of toxicity, with a dose reduction to be considered as needed [35]
Intestinal absorption/transport → CBD + everolimus (perpetrator)	NONE ESTABLISHED. ABCB1 is polymorphic, but no study identified relates ABCB1 genotype to everolimus exposure under CBD	Measured in a controlled trial. CBD raises everolimus exposure with half-life not prolonged, consistent with a predominantly absorption-related mechanism, although the transporter and mechanism remain unconfirmed (Table 1)	No genotype-stratified convergence can be specified while the genetic modifier is unresolved; the transporter hypothesis is the same as in the intestinal P-glycoprotein row, and the two should not be counted as independent evidence	Trough monitoring with individualized dose adjustment when CBD is started. Label-supported, and supported by a controlled PK study
UGT1A9/UGT2B7 → CBD + UGT substrates (perpetrator)	NONE ESTABLISHED for cannabinoids. UGT polymorphisms are themselves determinants of mycophenolic acid exposure [86,87].	Measured in dedicated human probe studies. In these studies, CBD raised mycophenolic acid AUC +35% (58% predicted at the higher steady-state dose) and zidovudine AUC +19%; only UGT1A9 carries a labeled recommendation to consider a dose reduction, for substrates for which small concentration increases may cause serious adverse reactions, as clinically appropriate [5]	UGT genotype × CBD inhibition on a UGT1A9 substrate; an additional untested convergence hypothesis	Consider a dose reduction in UGT1A9 substrates, such as mycophenolic acid, for which small concentration increases may cause serious adverse reactions, as clinically appropriate. Label-supported
Glucuronidation capacity → nabiximols + bilirubin (pathway-level caution; not a core CEM relationship)	NOT A CONVERGENCE NODE. Gilbert’s disease reduces UGT1A1-mediated bilirubin glucuronidation, but UGT1A1 is not among the isoforms the labeling reports as inhibited	In vitro, with a regulatory caution. Nabiximols inhibits UGT1A9 and UGT2B7, not UGT1A1, at clinically attainable concentrations; the labeling advises caution in genetic glucuronidation disorders [3]	Pathway-level, not enzyme-level: bilirubin is cleared principally by UGT1A1, which the labeling does not report as inhibited. A regulator-stated caution, not a measured interaction	The labeling advises caution in genetic glucuronidation disorders; it does not direct bilirubin measurement. Any monitoring is author-generated, and no genotyping is indicated
Intestinal P-glycoprotein → CBD + oral P-gp substrates (perpetrator)	NONE ESTABLISHED. ABCB1 is polymorphic, but no study identified relates ABCB1 genotype to exposure for any approved cannabinoid or to co-substrate exposure under CBD	Labeled advice with an inferred compartment. Labeling advises therapeutic drug monitoring and dose reduction for orally administered P-gp substrates, citing in vivo data on intestinal efflux; steady-state plasma CBD lies below the inhibitory concentration, supporting a predominantly intestinal contribution [5]	ABCB1 genotype × intestinal CBD inhibition on an oral P-gp substrate; no data, and the compartment-restricted mechanism makes the genotype axis harder to isolate than at a hepatic enzyme node	Consider therapeutic drug monitoring and a dose reduction in oral P-glycoprotein co-substrates such as digoxin, sirolimus, and tacrolimus. Label-supported
BCRP and BSEP → 7-COOH-CBD (metabolite-mediated; exploratory)	NONE ESTABLISHED. No study identified relates ABCG2 (encoding BCRP) or ABCB11 (encoding BSEP) genotype to any cannabinoid-related outcome	In vitro only. 7-COOH-CBD, the most abundant circulating species, inhibits BCRP- and BSEP-mediated transport at clinically relevant concentrations [5]. There is no evidence of a P-glycoprotein interaction, and the substrate sets differ from P-gp	None specified. BSEP inhibition bears on cholestatic liability rather than on co-substrate exposure and is better read alongside the hepatic signal than as an exposure-modifier node	None. Exploratory; generates no prediction in Box 1

*Notes.* This is the primary operational table of the framework. Rows are organized by clearance pathway and product. The genotype column is tagged by maturity: ESTABLISHED where a genotype-exposure or genotype-clearance relationship is quantified, and NONE ESTABLISHED where no study identified relates genotype to exposure for that pairing. One row is tagged NOT A CONVERGENCE NODE: the genotype and the inhibited isoforms differ, so it is a pathway-level regulatory caution and not a core CEM relationship (Section 5.3). The comedication column is headed by evidence type rather than by design, because its rows differ: some report effects measured in controlled human studies, others rest on in vitro findings, labeled advice, or inference from enzyme inhibition. Each row states which applies. The convergence column is predicted and untested for every row, since no approved-product study identified in this review has measured a genotype-by-treatment effect. Rows tagged NONE ESTABLISHED therefore carry a populated interaction axis and an empty genotype axis, and should not be read as equal in maturity to the CYP2C19-clobazam row. Maturity is also not transitive along a row: an established genotype relationship together with a measured comedication effect strengthens the biological rationale for the convergence entry in the same row, but does not validate it, because that entry is a prediction about their joint effect and no such effect has been measured. The three column tags are to be read independently, not accumulated across the row. Where a prediction is framed for a row, its Box 1 identifier is given in the convergence cell, so the prediction and its falsification criterion travel together; rows carrying no identifier have no prediction framed in Box 1. The monitoring column labels every measure with the actionability categories defined in Section 2, as Table 3 does. Box 1 identifiers link each prediction to its falsification criterion.

**Table 3:** Exposure-informed monitoring scenarios for approved cannabinoid medicines.

Clinical Scenario	Mechanistic Concern	Evidence Level	Monitoring Action	Actionability
CBD + valproate	Hepatotoxicity not explained by measured systemic exposure of either drug; mechanism unexplained	Controlled trials; label	Transaminases and total bilirubin before treatment, at 1, 3, and 6 months, periodically thereafter, and within 1 month of any dosage change or addition of a medicine known to affect the liver; consider more frequent monitoring on valproate	Label-supported action
CBD + clobazam	CYP2C19 inhibition raises exposure to the active metabolite N-desmethylclobazam	Controlled PK study; label	Monitor somnolence and sedation; consider clobazam dose reduction if they occur	Controlled-PK-supported monitoring; label-supported action
CBD + tacrolimus	CBD raises tacrolimus exposure severalfold; mechanism undetermined	Controlled PK study; labels of both products	Closely monitor tacrolimus whole-blood concentrations and for signs of tacrolimus toxicity when CBD is started; consider a tacrolimus dose reduction as needed	Label-supported action
CBD + everolimus	Everolimus exposure rises ≈2.5-fold with half-life not prolonged; mechanism unconfirmed	Controlled PK study; label	Monitor everolimus therapeutic drug levels and adjust the dosage accordingly; in patients on stable CBD, initiate everolimus at a lower starting dosage and titrate on monitoring	Label-supported action
CBD + mycophenolate mofetil or another sensitive UGT1A9 substrate	UGT1A9 inhibition raises mycophenolic acid exposure (AUC +35%; 58% predicted at the higher steady-state dose)	Human probe study; label	Consider a dose reduction in the UGT1A9 substrate where small concentration increases may cause serious adverse reactions, as clinically appropriate	Label-supported action
CBD + zidovudine (UGT2B7 substrate)	UGT2B7 inhibition raises zidovudine exposure (AUC +19%), judged not clinically significant	Human probe study	The labeling describes the zidovudine exposure change as not clinically significant and specifies no interaction-specific monitoring or dose adjustment; routine care for the substrate is unaffected. Not extended to other UGT2B7 substrates, which were not studied (Section 5.3)	Label-supported: no interaction-specific action indicated
CBD + oral P-glycoprotein substrate (digoxin, sirolimus, tacrolimus)	Intestinal P-gp inhibition; the labeling cites in vivo data on intestinal efflux, but no dedicated probe study in an approved product was identified	Label-supported; no dedicated study	Consider therapeutic drug monitoring and dosage reduction in the P-gp substrate	Label-supported action
CBD + warfarin	Mechanism unassigned; CYP2C9 inhibition not supported by the negative tolbutamide probe study	Two approved-product case reports; predominantly indirect literature	Monitor the international normalized ratio at initiation and at each dose change	Author-generated vigilance
CBD + rifampin or another strong CYP3A4/CYP2C19 inducer; and, by pathway assignment only, enzyme-inducing antiseizure medicines	Induction lowers CBD AUC 32% and 7-OH-CBD AUC 63% with rifampin. For enzyme-inducing antiseizure medicines, the pathway assignment is the same, but the effect on CBD and 7-OH-CBD is unquantified	Dedicated study for rifampin; mechanistic inference only for antiseizure inducers	Watch for loss of seizure control rather than toxicity; consider a CBD dose increase up to twofold, guided by clinical response and tolerability. Unexplained loss of response should prompt review of concomitant inducers, though no threshold can be set for the antiseizure agents	Label-supported action for strong CYP3A4 or CYP2C19 inducers as a class, of which rifampin is the studied example; the drug-specific magnitude is unquantified for the others, and the extension to enzyme-inducing antiseizure medicines, which are not all strong inducers, is research only
Administration relative to food (CBD and nabiximols)	A high-fat, high-calorie meal raises CBD AUC ≈ 4× and C_max_ ≈ 5×; for nabiximols, food raises THC AUC ≈ 2.8× and CBD AUC ≈ 5.1×	Dedicated food-effect studies	Dose consistently with respect to meals; standardize administration as far as possible in relation to food intake, to reduce variability in plasma exposure	Label-supported action
Nabiximols + a CYP2C9 inhibitor (fluconazole)	THC exposure rises, and 11-OH-THC exposure rises proportionally more, so the active metabolite moves further than the parent	Controlled PK study in label	Take care when co-administering; anticipate increased psychoactive and sedative effects	Label-supported action
Nabiximols + a strong CYP3A4 inhibitor or inducer	Inhibition raises, and induction lowers, THC, CBD and 11-OH-THC	Direct product-specific PK study	A new dose titration may be required when the interacting drug is started or stopped	Label-supported action
Nabiximols in moderate or severe hepatic impairment	Substantially reduced clearance and prolonged elimination half-life	Dedicated PK study in label	Administration is not advised; use in mild impairment requires no dose adjustment	Label-supported action
Nabiximols in moderate or severe renal impairment	11-OH-THC and 7-OH-CBD rise three- to four-and-a-half-fold, while parent exposure rises approximately twofold for CBD and less than twofold for THC	Dedicated PK study in label	Lower daily doses may be needed	Label-supported action
Oral dronabinol or THC in likely reduced CYP2C9 function, including CYP2C9*3 carriers	Reduced CYP2C9 activity raises parent THC exposure severalfold; parent THC, 11-OH-THC and THC-COOH must be measured separately	Small pharmacogenomic study; label	Monitor for increased adverse reactions; the label advises caution rather than genotyping and defines no genotype-guided dose	Label-supported action
Older or fall-prone patients receiving THC-containing or THC-analog medicines	Orthostatic hypotension, sedation and dizziness may raise fall risk; exposure was not measured in the source studies	Systematic review and meta-analysis	Review balance-impairing comedications and postural symptoms	Author-generated vigilance

*Notes.* Table 3 translates the evidence characterized in Table 1 into practical monitoring scenarios. It reports the principal evidence source and actionability but does not repeat all four reported fields. Evidence from controlled studies of an approved product supports the listed monitoring actions, whereas evidence carrying the limitations recorded in Table 1 supports vigilance and prospective study rather than firm recommendations. The final column uses the four actionability categories defined in Section 2. Where two apply to one scenario, both are given. No additional or composite evidence scale is used. The table prioritizes monitoring and study design. It retains the dose adjustments the labeling already specifies, such as considering an increase of up to twofold with a strong inducer; what the framework adds is no additional quantitative dosing rule, because no method has been validated for translating a parent–active-metabolite profile into an individualized concentration target or dose (Section 3.1). No genotype-guided dosing recommendation currently exists for any approved cannabinoid. Rows marked as author-generated vigilance are research-oriented considerations, not validated clinical management recommendations.

## Data Availability

No new data were created or analyzed in this study. Data sharing is not applicable to this article.

## Supplementary Materials

The following supporting information can be downloaded at https://www.mdpi.com/article/10.3390/pharmaceutics18101293/s1: Table S1: Search documentation; Figure S1: Evidence-selection and characterization schema for the narrative integrative review; Table S2: Structured descriptive critical appraisal of pivotal studies.

## List of Abbreviations

The following abbreviations are used in this manuscript:

7-COOH-CBD	7-carboxy-cannabidiol
7-OH-CBD	7-hydroxy-cannabidiol
11-OH-THC	11-hydroxy-Δ^9^-tetrahydrocannabinol
ABCB1	ATP-binding cassette subfamily B member 1 (gene encoding P-glycoprotein)
ABCB11	ATP-binding cassette subfamily B member 11 (gene encoding the bile salt export pump)
ABCG2	ATP-binding cassette subfamily G member 2 (gene encoding the breast cancer resistance protein)
AUC	area under the concentration–time curve
BCRP	breast cancer resistance protein
BSEP	bile salt export pump
CBD	cannabidiol
CEM	convergent exposure-modifier
C_max_	maximum plasma concentration
CYP	cytochrome P450
FABP1	liver fatty acid-binding protein 1
FDA	U.S. Food and Drug Administration
IC_50_	half-maximal inhibitory concentration
P-gp	P-glycoprotein (encoded by ABCB1)
PK	pharmacokinetic
PRISMA	Preferred Reporting Items for Systematic Reviews and Meta-Analyses
THC	Δ^9^-tetrahydrocannabinol
THC-COOH	11-nor-9-carboxy-Δ^9^-tetrahydrocannabinol
UGT	UDP-glucuronosyltransferase

## References

[1] Thiele E.A., Bebin E.M., Bhathal H., Jansen F.E., Kotulska K., Lawson J.A., O’Callaghan F.J., Wong M., Sahebkar F., Checketts D. (2021). Add-on Cannabidiol Treatment for Drug-Resistant Seizures in Tuberous Sclerosis Complex: A Placebo-Controlled Randomized Clinical Trial. JAMA Neurol..

[2] Novotna A., Mares J., Ratcliffe S., Novakova I., Vachova M., Zapletalova O., Gasperini C., Pozzilli C., Cefaro L., Comi G. (2011). A Randomized, Double-Blind, Placebo-Controlled, Parallel-Group, Enriched-Design Study of Nabiximols* (Sativex^®^), as Add-on Therapy, in Subjects with Refractory Spasticity Caused by Multiple Sclerosis: Sativex for Refractory Spasticity in MS. Eur. J. Neurol..

[3] SVX Therapeutics Limited Sativex Oromucosal Spray: Summary of Product Characteristics. https://www.medicines.org.uk/emc/product/602/smpc.

[4] Bajtel Á., Kiss T., Tóth B., Kiss S., Hegyi P., Vörhendi N., Csupor-Löffler B., Gede N., Hohmann J., Csupor D. (2022). The Safety of Dronabinol and Nabilone: A Systematic Review and Meta-Analysis of Clinical Trials. Pharmaceuticals.

[5] U.S. Food and Drug Administration EPIDIOLEX (Cannabidiol) Oral Solution: Prescribing Information, Revised 05/2026. https://www.accessdata.fda.gov/drugsatfda_docs/label/2026/210365s024s029s030lbl.pdf.

[6] U.S. Food and Drug Administration CESAMET (Nabilone) Capsules: Prescribing Information. https://www.accessdata.fda.gov/drugsatfda_docs/label/2022/018677Orig1s017lbl.pdf.

[7] Johannessen Landmark C., Sætre J., Gottås A., Wolden M., McQuade T.A., Kjeldsen S.F., Våtevik A., Sætre E., Svendsen T., Burns M.L. (2025). Pharmacokinetic Variability and Use of Therapeutic Drug Monitoring of Cannabidiol in Patients with Refractory Epilepsy. Epilepsia.

[8] Velayudhan L., McGoohan K.L., Bhattacharyya S. (2021). Evaluation of THC-Related Neuropsychiatric Symptoms Among Adults Aged 50 Years and Older: A Systematic Review and Metaregression Analysis. JAMA Netw. Open.

[9] Turner R.M., De Koning E.M., Fontana V., Thompson A., Pirmohamed M. (2020). Multimorbidity, Polypharmacy, and Drug-Drug-Gene Interactions Following a Non-ST Elevation Acute Coronary Syndrome: Analysis of a Multicentre Observational Study. BMC Med..

[10] Ammendolia I., Mannucci C., Cardia L., Calapai G., Gangemi S., Esposito E., Calapai F. (2023). Pharmacovigilance on Cannabidiol as an Antiepileptic Agent. Front. Pharmacol..

[11] Calapai F., Mannucci C., McQuain L., Salvo F. (2023). Pharmacological Evaluation of Signals of Disproportionality Reporting Related to Adverse Reactions to Antiepileptic Cannabidiol in VigiBase. Pharmaceuticals.

[12] Huestis M.A. (2007). Human Cannabinoid Pharmacokinetics. Chem. Biodivers..

[13] Lucas C.J., Galettis P., Schneider J. (2018). The Pharmacokinetics and the Pharmacodynamics of Cannabinoids. Br. J. Clin. Pharmacol..

[14] Millar S.A., Stone N.L., Yates A.S., O’Sullivan S.E. (2018). A Systematic Review on the Pharmacokinetics of Cannabidiol in Humans. Front. Pharmacol..

[15] Malki M.A., Pearson E.R. (2020). Drug–Drug–Gene Interactions and Adverse Drug Reactions. Pharmacogenomics J..

[16] Bruckmueller H., Cascorbi I. (2021). Drug-Drug-Gene Interactions: A Call for Clinical Consideration. Clin. Pharmacol. Ther..

[17] Rüdesheim S., Loer H.L.H., Feick D., Marok F.Z., Fuhr L.M., Selzer D., Teutonico D., Schneider A.R.P., Solodenko J., Frechen S. (2025). A Comprehensive CYP2D6 Drug–Drug–Gene Interaction Network for Application in Precision Dosing and Drug Development. Clin. Pharmacol. Ther..

[18] Dean L., Kane M., Pratt V.M., Scott S.A., Pirmohamed M., Esquivel B., Kattman B.L., Malheiro A.J. (2020). Dronabinol Therapy and CYP2C9 Genotype. Medical Genetics Summaries.

[19] Hryhorowicz S., Walczak M., Zakerska-Banaszak O., Słomski R., Skrzypczak-Zielińska M. (2018). Pharmacogenetics of Cannabinoids. Eur. J. Drug Metab. Pharmacokinet..

[20] Nachnani R., Knehans A., Neighbors J.D., Kocis P.T., Lee T., Tegeler K., Trite T., Raup-Konsavage W.M., Vrana K.E. (2024). Systematic Review of Drug-Drug Interactions of Delta-9-Tetrahydrocannabinol, Cannabidiol, and Cannabis. Front. Pharmacol..

[21] Papakyriakopoulou P., Valsami G., Ismailos G. (2026). Cannabinoids and Drug–Drug Pharmacokinetic Interactions: Deciphering the Risks. Br. J. Clin. Pharmacol..

[22] Na J.-H., Lee Y.-M. (2025). Pharmacological and Pharmacokinetic Profile of Cannabidiol in Human Epilepsy: A Review of Metabolism, Therapeutic Drug Monitoring, and Interactions with Antiseizure Medications. Biomolecules.

[23] Kalak M., Brylak-Błaszków A., Błaszków Ł., Kalak T. (2025). Medical Marijuana and Treatment Personalization: The Role of Genetics and Epigenetics in Response to THC and CBD. Genes.

[24] Caicedo D.A., Pérez-Mañá C., Farré M., Papaseit E. (2025). An Overview of the Potential for Pharmacokinetic Interactions Between Drugs and Cannabis Products in Humans. Pharmaceutics.

[25] Guo H.-L., Xu Y.-Y., Li Y., Chen F. (2026). Toward Individualized Dosing of Perampanel: Integrating Therapeutic Drug Monitoring, Drug–Drug Interactions, and Pharmacogenetics. Eur. J. Pharm. Sci..

[26] Page M.J., McKenzie J.E., Bossuyt P.M., Boutron I., Hoffmann T.C., Mulrow C.D., Shamseer L., Tetzlaff J.M., Akl E.A., Brennan S.E. (2021). The PRISMA 2020 Statement: An Updated Guideline for Reporting Systematic Reviews. BMJ.

[27] Baethge C., Goldbeck-Wood S., Mertens S. (2019). SANRA—A Scale for the Quality Assessment of Narrative Review Articles. Res. Integr. Peer Rev..

[28] Sachse-Seeboth C., Pfeil J., Sehrt D., Meineke I., Tzvetkov M., Bruns E., Poser W., Vormfelde S., Brockmöller J. (2009). Interindividual Variation in the Pharmacokinetics of Δ9-Tetrahydrocannabinol as Related to Genetic Polymorphisms in CYP2C9. Clin. Pharmacol. Ther..

[29] U.S. Food and Drug Administration MARINOL (Dronabinol) Capsules: Prescribing Information. https://www.accessdata.fda.gov/drugsatfda_docs/label/2026/018651s036lbl.pdf.

[30] Geffrey A.L., Pollack S.F., Bruno P.L., Thiele E.A. (2015). Drug–Drug Interaction between Clobazam and Cannabidiol in Children with Refractory Epilepsy. Epilepsia.

[31] Morrison G., Crockett J., Blakey G., Sommerville K. (2019). A Phase 1, Open-Label, Pharmacokinetic Trial to Investigate Possible Drug-Drug Interactions Between Clobazam, Stiripentol, or Valproate and Cannabidiol in Healthy Subjects. Clin. Pharmacol. Drug Dev..

[32] VanLandingham K.E., Crockett J., Taylor L., Morrison G. (2020). A Phase 2, Double-Blind, Placebo-Controlled Trial to Investigate Potential Drug-Drug Interactions Between Cannabidiol and Clobazam. J. Clin. Pharma.

[33] So G.C., Lu J.B.L., Koyama S., Cheng Y., Gisch D.L., McClara K., Dexter P.R., Sharfuddin A.A., Etkins J., Tillman E.M. (2025). A Phase I Trial of the Pharmacokinetic Interaction Between Cannabidiol and Tacrolimus. Clin. Pharmacol. Ther..

[34] Leino A.D., Emoto C., Fukuda T., Privitera M., Vinks A.A., Alloway R.R. (2019). Evidence of a Clinically Significant Drug-Drug Interaction between Cannabidiol and Tacrolimus. Am. J. Transplant..

[35] U.S. Food and Drug Administration PROGRAF (Tacrolimus) Capsules, for Oral Use; PROGRAF (Tacrolimus) Injection, for Intravenous Use; PROGRAF Granules (Tacrolimus for Oral Suspension): Prescribing Information. https://www.accessdata.fda.gov/drugsatfda_docs/label/2023/050708s055,210115s007lbl.pdf.

[36] Wiemer-Kruel A., Stiller B., Bast T. (2019). Cannabidiol Interacts Significantly with Everolimus—Report of a Patient with Tuberous Sclerosis Complex. Neuropediatrics.

[37] Wray L., Berwaerts J., Critchley D., Hyland K., Chen C., Thai C., Tayo B. (2023). Pharmacokinetic Drug-Drug Interaction with Coadministration of Cannabidiol and Everolimus in a Phase 1 Healthy Volunteer Trial. Clin. Pharmacol. Drug Dev..

[38] Stott C., White L., Wright S., Wilbraham D., Guy G. (2013). A Phase I, Open-Label, Randomized, Crossover Study in Three Parallel Groups to Evaluate the Effect of Rifampicin, Ketoconazole, and Omeprazole on the Pharmacokinetics of THC/CBD Oromucosal Spray in Healthy Volunteers. SpringerPlus.

[39] Beers J.L., Fu D., Jackson K.D. (2021). Cytochrome P450–Catalyzed Metabolism of Cannabidiol to the Active Metabolite 7-Hydroxy-Cannabidiol. Drug Metab. Dispos..

[40] Etkins J., So G.C., Lu J.B.L., Koyama S., Gisch D.L., Melo Ferreira R., Cheng Y., McClara K., Jun J., Miller M. (2026). Genotype–Specific Safety and Pharmacokinetics of Cannabidiol in Healthy Volunteers. Clin. Transl. Sci..

[41] Cortopassi J. (2020). Warfarin Dose Adjustment Required after Cannabidiol Initiation and Titration. Am. J. Health Syst. Pharm..

[42] Grayson L., Vines B., Nichol K., Szaflarski J.P. (2018). An Interaction between Warfarin and Cannabidiol, a Case Report. Epilepsy Behav. Case Rep..

[43] Smythe M.A., Wu W., Garwood C.L. (2023). Anticoagulant Drug–Drug Interactions with Cannabinoids: A Systematic Review. Pharmacotherapy.

[44] Bansal S., Zamarripa C.A., Spindle T.R., Weerts E.M., Thummel K.E., Vandrey R., Paine M.F., Unadkat J.D. (2023). Evaluation of Cytochrome P450-Mediated Cannabinoid-Drug Interactions in Healthy Adult Participants. Clin. Pharmacol. Ther..

[45] Cáceres Guido P., Riva N., Caraballo R., Reyes G., Huaman M., Gutierrez R., Agostini S., Fabiana Delaven S., Pérez Montilla C.A., García Bournissen F. (2021). Pharmacokinetics of Cannabidiol in Children with Refractory Epileptic Encephalopathy. Epilepsia.

[46] Birnbaum A.K., Karanam A., Marino S.E., Barkley C.M., Remmel R.P., Roslawski M., Gramling-Aden M., Leppik I.E. (2019). Food Effect on Pharmacokinetics of Cannabidiol Oral Capsules in Adult Patients with Refractory Epilepsy. Epilepsia.

[47] Taylor L., Gidal B., Blakey G., Tayo B., Morrison G. (2018). A Phase I, Randomized, Double-Blind, Placebo-Controlled, Single Ascending Dose, Multiple Dose, and Food Effect Trial of the Safety, Tolerability and Pharmacokinetics of Highly Purified Cannabidiol in Healthy Subjects. CNS Drugs.

[48] Thiele E.A., Marsh E.D., French J.A., Mazurkiewicz-Beldzinska M., Benbadis S.R., Joshi C., Lyons P.D., Taylor A., Roberts C., Sommerville K. (2018). Cannabidiol in Patients with Seizures Associated with Lennox-Gastaut Syndrome (GWPCARE4): A Randomised, Double-Blind, Placebo-Controlled Phase 3 Trial. Lancet.

[49] Crockett J., Critchley D., Tayo B., Berwaerts J., Morrison G. (2020). A Phase 1, Randomized, Pharmacokinetic Trial of the Effect of Different Meal Compositions, Whole Milk, and Alcohol on Cannabidiol Exposure and Safety in Healthy Subjects. Epilepsia.

[50] Ujváry I., Hanuš L. (2016). Human Metabolites of Cannabidiol: A Review on Their Formation, Biological Activity, and Relevance in Therapy. Cannabis Cannabinoid Res..

[51] U.S. Food and Drug Administration EPIDIOLEX (Cannabidiol) Oral Solution: Prescribing Information, Revised 06/2025. https://www.accessdata.fda.gov/drugsatfda_docs/label/2025/210365s023lbl.pdf.

[52] Taylor L., Crockett J., Tayo B., Morrison G. (2019). A Phase 1, Open-Label, Parallel-Group, Single-Dose Trial of the Pharmacokinetics and Safety of Cannabidiol (CBD) in Subjects With Mild to Severe Hepatic Impairment. J. Clin. Pharma.

[53] Bansal S., Ladumor M.K., Paine M.F., Unadkat J.D. (2023). A Physiologically-Based Pharmacokinetic Model for Cannabidiol in Healthy Adults, Hepatically-Impaired Adults, and Children. Drug Metab. Dispos..

[54] Jiang R., Yamaori S., Takeda S., Yamamoto I., Watanabe K. (2011). Identification of Cytochrome P450 Enzymes Responsible for Metabolism of Cannabidiol by Human Liver Microsomes. Life Sci..

[55] Mazur A., Lichti C.F., Prather P.L., Zielinska A.K., Bratton S.M., Gallus-Zawada A., Finel M., Miller G.P., Radomińska-Pandya A., Moran J.H. (2009). Characterization of Human Hepatic and Extrahepatic UDP-Glucuronosyltransferase Enzymes Involved in the Metabolism of Classic Cannabinoids. Drug Metab. Dispos..

[56] Tayo B., Taylor L., Sahebkar F., Morrison G. (2020). A Phase I, Open-Label, Parallel-Group, Single-Dose Trial of the Pharmacokinetics, Safety, and Tolerability of Cannabidiol in Subjects with Mild to Severe Renal Impairment. Clin. Pharmacokinet..

[57] Jiang R., Yamaori S., Okamoto Y., Yamamoto I., Watanabe K. (2013). Cannabidiol Is a Potent Inhibitor of the Catalytic Activity of Cytochrome P450 2C19. Drug Metab. Pharmacokinet..

[58] Bland T.M., Haining R.L., Tracy T.S., Callery P.S. (2005). CYP2C-Catalyzed Delta(9)-Tetrahydrocannabinol Metabolism: Kinetics, Pharmacogenetics and Interaction with Phenytoin. Biochem. Pharmacol..

[59] Watanabe K., Yamaori S., Funahashi T., Kimura T., Yamamoto I. (2007). Cytochrome P450 Enzymes Involved in the Metabolism of Tetrahydrocannabinols and Cannabinol by Human Hepatic Microsomes. Life Sci..

[60] Wolowich W.R., Greif R., Kleine-Brueggeney M., Bernhard W., Theiler L. (2019). Minimal Physiologically Based Pharmacokinetic Model of Intravenously and Orally Administered Delta-9-Tetrahydrocannabinol in Healthy Volunteers. Eur. J. Drug Metab. Pharmacokinet..

[61] Qian L., Beers J.L., Jackson K.D., Zhou Z. (2024). CBD and THC in Special Populations: Pharmacokinetics and Drug-Drug Interactions. Pharmaceutics.

[62] Stott C.G., White L., Wright S., Wilbraham D., Guy G.W. (2013). A Phase I Study to Assess the Effect of Food on the Single Dose Bioavailability of the THC/CBD Oromucosal Spray. Eur. J. Clin. Pharmacol..

[63] Karschner E.L., Darwin W.D., Goodwin R.S., Wright S., Huestis M.A. (2011). Plasma Cannabinoid Pharmacokinetics Following Controlled Oral Delta9-Tetrahydrocannabinol and Oromucosal Cannabis Extract Administration. Clin. Chem..

[64] Fusaroli M., Salvo F., Begaud B., AlShammari T.M., Bate A., Battini V., Brueckner A., Candore G., Carnovale C., Crisafulli S. (2024). The Reporting of a Disproportionality Analysis for Drug Safety Signal Detection Using Individual Case Safety Reports in PharmacoVigilance (READUS-PV): Development and Statement. Drug Saf..

[65] Caster O., Aoki Y., Gattepaille L.M., Grundmark B. (2020). Disproportionality Analysis for Pharmacovigilance Signal Detection in Small Databases or Subsets: Recommendations for Limiting False-Positive Associations. Drug Saf..

[66] Calonge Q., Besnard A., Bailly L., Damiano M., Pichit P., Dupont S., Gourfinkel-An I., Navarro V. (2024). Cannabidiol Treatment for Adult Patients with Drug-Resistant Epilepsies: A Real-World Study in a Tertiary Center. Brain Behav..

[67] Chesney E., Oliver D., Green A., Sovi S., Wilson J., Englund A., Freeman T.P., McGuire P. (2020). Adverse Effects of Cannabidiol: A Systematic Review and Meta-Analysis of Randomized Clinical Trials. Neuropsychopharmacology.

[68] Devinsky O., Patel A.D., Cross J.H., Villanueva V., Wirrell E.C., Privitera M., Greenwood S.M., Roberts C., Checketts D., VanLandingham K.E. (2018). Effect of Cannabidiol on Drop Seizures in the Lennox-Gastaut Syndrome. N. Engl. J. Med..

[69] Devinsky O., Cross J.H., Laux L., Marsh E., Miller I., Nabbout R., Scheffer I.E., Thiele E.A., Wright S. (2017). Trial of Cannabidiol for Drug-Resistant Seizures in the Dravet Syndrome. N. Engl. J. Med..

[70] Ben-Menachem E., Gunning B., Arenas Cabrera C.M., VanLandingham K., Crockett J., Critchley D., Wray L., Tayo B., Morrison G., Toledo M. (2020). A Phase II Randomized Trial to Explore the Potential for Pharmacokinetic Drug–Drug Interactions with Stiripentol or Valproate When Combined with Cannabidiol in Patients with Epilepsy. CNS Drugs.

[71] Lakhani V.V., Generaux G., Howell B.A., Longo D.M., Watkins P.B. (2023). Assessing Liver Effects of Cannabidiol and Valproate Alone and in Combination Using Quantitative Systems Toxicology. Clin. Pharmacol. Ther..

[72] Beers J.L., Harvey S.L., Lanphier R.M., Rushing B.R., McRitchie S.L., Sumner S.J., Jackson K.D. (2025). Evaluating Cannabidiol-Induced Liver Injury with and without Valproate Using a Three-Dimensional Human Hepatocyte Spheroid Model. Toxicol. Vitr..

[73] Watkins P.B., Church R.J., Li J., Knappertz V. (2021). Cannabidiol and Abnormal Liver Chemistries in Healthy Adults: Results of a Phase I Clinical Trial. Clin. Pharma. Ther..

[74] Gaston T.E., Bebin E.M., Cutter G.R., Liu Y., Szaflarski J.P. (2017). The UAB CBD Program Interactions between Cannabidiol and Commonly Used Antiepileptic Drugs. Epilepsia.

[75] Watanabe A.H., Navaravong L., Sirilak T., Prasitwarachot R., Nathisuwan S., Page R.L., Chaiyakunapruk N. (2021). A Systematic Review and Meta-Analysis of Randomized Controlled Trials of Cardiovascular Toxicity of Medical Cannabinoids. J. Am. Pharm. Assoc..

[76] Schiro P., Gálvez Pacoricona M., Le Guilloux A., Déguilhem A., Merad S., Bouquet E., Auffret M., Micallef J., Lapeyre-Mestre M., Jouanjus E. (2026). Cannabis and Cannabinoid-Related Suspected Adverse Drug Reactions: Global Patterns and Cross-National Analysis Based on VigiBase^®^ Data. CNS Drugs.

[77] Hollister L.E., Gillespie H.K. (1975). Action of Delta-9-tetrahydrocannabinol An Approach to the Active Metabolite Hypothesis. Clin. Pharmacol. Ther..

[78] Patilea-Vrana G.I., Unadkat J.D. (2019). Quantifying Hepatic Enzyme Kinetics of (-)-∆9-Tetrahydrocannabinol (THC) and Its Psychoactive Metabolite, 11-OH-THC, through In Vitro Modeling. Drug Metab. Dispos..

[79] Yabut K.C.B., Winnie Wen Y., Simon K.T., Isoherranen N. (2024). CYP2C9, CYP3A and CYP2C19 Metabolize Δ9-Tetrahydrocannabinol to Multiple Metabolites but Metabolism Is Affected by Human Liver Fatty Acid Binding Protein (FABP1). Biochem. Pharmacol..

[80] Gemma A., Mauri M., Banfi P., Versino M., Zollo A., Boneschi F.M., Marino F., Cosentino M., Ferrari M. (2025). ABCB1 Polymorphisms Are Associated with Clinical Response to Nabiximols in Patients with Multiple Sclerosis-Related Spasticity. J. Cannabis. Res..

[81] Caudle K.E., Dunnenberger H.M., Freimuth R.R., Peterson J.F., Burlison J.D., Whirl-Carrillo M., Scott S.A., Rehm H.L., Williams M.S., Klein T.E. (2017). Standardizing Terms for Clinical Pharmacogenetic Test Results: Consensus Terms from the Clinical Pharmacogenetics Implementation Consortium (CPIC). Genet. Med..

[82] Nasrin S., Watson C.J.W., Bardhi K., Fort G., Chen G., Lazarus P. (2021). Inhibition of UDP-Glucuronosyltransferase Enzymes by Major Cannabinoids and Their Metabolites. Drug Metab. Dispos..

[83] Coates S., Bardhi K., Zhao M., Lazarus P. (2025). UGT2B7-Mediated Drug–Drug Interaction between Cannabinoids and Hydromorphone. Drug Metab. Dispos..

[84] Coates S., Bardhi K., Prasad B., Lazarus P. (2024). Evaluation of the Drug–Drug Interaction Potential of Cannabidiol Against UGT2B7-Mediated Morphine Metabolism Using Physiologically Based Pharmacokinetic Modeling. Pharmaceutics.

[85] Kahma H., Filppula A.M., Neuvonen M., Tarkiainen E.K., Tornio A., Holmberg M.T., Itkonen M.K., Finel M., Neuvonen P.J., Niemi M. (2018). Clopidogrel Carboxylic Acid Glucuronidation Is Mediated Mainly by UGT2B7, UGT2B4, and UGT2B17: Implications for Pharmacogenetics and Drug-Drug Interactions. Drug Metab. Dispos..

[86] Na Takuathung M., Sakuludomkan W., Koonrungsesomboon N. (2021). The Impact of Genetic Polymorphisms on the Pharmacokinetics and Pharmacodynamics of Mycophenolic Acid: Systematic Review and Meta-Analysis. Clin. Pharmacokinet..

[87] Xie X., Li J., Wang H., Li H., Liu J., Fu Q., Huang J., Zhu C., Zhong G., Wang X. (2015). Associations of UDP-Glucuronosyltransferases Polymorphisms with Mycophenolate Mofetil Pharmacokinetics in Chinese Renal Transplant Patients. Acta Pharmacol. Sin..

[88] Rubin A., Lemberger L., Warrick P., Crabtree R.E., Sullivan H., Rowe H., Obermeyer B.D. (1977). Physiologic Disposition of Nabilone, a Cannabinol Derivative, in Man. Clin. Pharmacol. Ther..

[89] Amann F., Hefner G., Cremer-Schaeffer P., Gründer G., Häuser W., Hart X.M., Petzke F., Zernig G., Havemann-Reinecke U. (2026). Therapeutic Drug Monitoring of Cannabinoids: Therapeutic Reference Ranges for Delta-9-Tetrahydrocannabinol in Medical Cannabis, Nabiximols, Dronabinol and Nabilone. Pharmacopsychiatry.

[90] Stott C.G., White L., Wright S., Wilbraham D., Guy G.W. (2013). A Phase I Study to Assess the Single and Multiple Dose Pharmacokinetics of THC/CBD Oromucosal Spray. Eur. J. Clin. Pharmacol..

[91] Auzmendi J., Palestro P., Blachman A., Gavernet L., Merelli A., Talevi A., Calabrese G.C., Ramos A.J., Lazarowski A. (2020). Cannabidiol (CBD) Inhibited Rhodamine-123 Efflux in Cultured Vascular Endothelial Cells and Astrocytes Under Hypoxic Conditions. Front. Behav. Neurosci..

[92] Zhu H.-J., Wang J.-S., Markowitz J.S., Donovan J.L., Gibson B.B., Gefroh H.A., DeVane C.L. (2006). Characterization of P-Glycoprotein Inhibition by Major Cannabinoids from Marijuana. J. Pharmacol. Exp. Ther..

[93] Poli P., Peruzzi L., Maurizi P., Mencucci A., Scocca A., Carnevale S., Spiga O., Santucci A. (2022). The Pharmacogenetics of Cannabis in the Treatment of Chronic Pain. Genes.

[94] Benyamina A., Bonhomme-Faivre L., Picard V., Sabbagh A., Richard D., Blecha L., Rahioui H., Karila L., Lukasiewicz M., Farinotti R. (2009). Association between ABCB1 C3435T Polymorphism and Increased Risk of Cannabis Dependence. Prog. Neuro-Psychopharmacol. Biol. Psychiatry.

[95] Matheson J., Zhang Y.J., Brands B., Wickens C.M., Tiwari A.K., Zai C.C., Kennedy J.L., Le Foll B. (2022). Association between ABCB1 Rs2235048 Polymorphism and THC Pharmacokinetics and Subjective Effects Following Smoked Cannabis in Young Adults. Brain Sci..

[96] Kebir O., Lafaye G., Blecha L., Chaumette B., Mouaffak F., Laqueille X., Benyamina A. (2018). ABCB1 C3435T Polymorphism Is Associated with Tetrahydrocannabinol Blood Levels in Heavy Cannabis Users. Psychiatry Res..

[97] Nahid N.A., Johnson J.A. (2022). CYP2D6 Pharmacogenetics and Phenoconversion in Personalized Medicine. Expert Opin. Drug Metab. Toxicol..

[98] Shah R.R., Smith R.L. (2015). Inflammation-Induced Phenoconversion of Polymorphic Drug Metabolizing Enzymes: Hypothesis with Implications for Personalized Medicine. Drug Metab. Dispos..

[99] Shah R.R., Smith R.L. (2015). Addressing Phenoconversion: The Achilles’ Heel of Personalized Medicine. Br. J. Clin. Pharmacol..

[100] Nanan J., Crosby S., Schuh M.J. (2022). Hyponatremic Cognitive Dysfunction Resulting from Drug-Drug-Gene Interaction between Sertraline and Cannabidiol in an Intermediate CYP2C19 Metabolizer Patient. Innov. Pharm..

[101] U.S. Food and Drug Administration In Vitro Drug Interaction Studies–Cytochrome P450 Enzyme- and Transporter-Mediated Drug Interactions: Guidance for Industry. https://www.fda.gov/media/135587/download.

[102] Matic M., Nijenhuis M., Soree B., De Boer-Veger N.J., Buunk A.-M., Houwink E.J.F., Mulder H., Rongen G.A.P.J.M., Weide J.V.D., Wilffert B. (2022). Dutch Pharmacogenetics Working Group (DPWG) Guideline for the Gene–Drug Interaction between CYP2D6 and Opioids (Codeine, Tramadol and Oxycodone). Eur. J. Hum. Genet..

[103] Lima J.J., Thomas C.D., Barbarino J., Desta Z., Van Driest S.L., El Rouby N., Johnson J.A., Cavallari L.H., Shakhnovich V., Thacker D.L. (2021). Clinical Pharmacogenetics Implementation Consortium (CPIC) Guideline for *CYP2C19* and Proton Pump Inhibitor Dosing. Clin. Pharmacol. Ther..

[104] Theken K.N., Lee C.R., Gong L., Caudle K.E., Formea C.M., Gaedigk A., Klein T.E., Agúndez J.A.G., Grosser T. (2020). Clinical Pharmacogenetics Implementation Consortium Guideline (CPIC) for *CYP2C9* and Nonsteroidal Anti-Inflammatory Drugs. Clin. Pharmacol. Ther..

[105] Wicha S.G., Märtson A., Nielsen E.I., Koch B.C.P., Friberg L.E., Alffenaar J., Minichmayr I.K. (2021). The International Society of Anti-Infective Pharmacology (ISAP), the PK/PD study group of the European Society of Clinical Microbiology, Infectious Diseases (EPASG) From Therapeutic Drug Monitoring to Model-Informed Precision Dosing for Antibiotics. Clin. Pharmacol. Ther..

[106] Patsalos P.N., Szaflarski J.P., Gidal B., VanLandingham K., Critchley D., Morrison G. (2020). Clinical Implications of Trials Investigating Drug-drug Interactions between Cannabidiol and Enzyme Inducers or Inhibitors or Common Antiseizure Drugs. Epilepsia.

[107] Strzelczyk A., Schubert-Bast S. (2022). A Practical Guide to the Treatment of Dravet Syndrome with Anti-Seizure Medication. CNS Drugs.

[108] So G.C., Lu J.B.L., Cheng Y., Gisch D.L., Koyama S., Melo Ferreira R., Beamon T.R., Desta Z., Eadon M.T. (2025). Inhibition of Tacrolimus Metabolism by Cannabidiol and Its Metabolites In Vitro. Clin. Transl. Sci..

[109] Yamaori S., Ebisawa J., Okushima Y., Yamamoto I., Watanabe K. (2011). Potent Inhibition of Human Cytochrome P450 3A Isoforms by Cannabidiol: Role of Phenolic Hydroxyl Groups in the Resorcinol Moiety. Life Sci..

[110] Birdwell K., Decker B., Barbarino J., Peterson J., Stein C., Sadee W., Wang D., Vinks A., He Y., Swen J. (2015). Clinical Pharmacogenetics Implementation Consortium (CPIC) Guidelines for *CYP3A5* Genotype and Tacrolimus Dosing. Clin. Pharmacol. Ther..

[111] Nasrin S., Watson C.J.W., Perez-Paramo Y.X., Lazarus P. (2021). Cannabinoid Metabolites as Inhibitors of Major Hepatic CYP450 Enzymes, with Implications for Cannabis-Drug Interactions. Drug Metab. Dispos..

[112] Thai C., Tayo B., Critchley D. (2021). A Phase 1 Open-Label, Fixed-Sequence Pharmacokinetic Drug Interaction Trial to Investigate the Effect of Cannabidiol on the CYP1A2 Probe Caffeine in Healthy Subjects. Clin. Pharm. Drug Dev..

[113] Salcedo P., Volpe D.A., Chaturbedi A., Shah A., Krishna A., Hyland P.L., Vegesna G., Hsiao C., De Palma R., Fein M. (2026). Clinical Study to Evaluate Drug Interactions of Cannabidiol with Citalopram and Morphine in Healthy Adults. Clin. Pharmacol. Ther..

[114] Gorbenko A.A., Post T.E., Strugala P.K., Klaassen E.S., Klumpers L.E., De Visser S.J., Sempio C., Klawitter J., Heuberger J.A.A.C., Groeneveld G.J. (2026). Low-dose Cannabidiol Increases Plasma Concentrations of Amitriptyline: A Clinical Drug–Drug Interaction Study. Br. J. Clin. Pharmacol..

[115] Lee C.R., Luzum J.A., Sangkuhl K., Gammal R.S., Sabatine M.S., Stein C.M., Kisor D.F., Limdi N.A., Lee Y.M., Scott S.A. (2022). Clinical Pharmacogenetics Implementation Consortium Guideline for *CYP2C19* Genotype and Clopidogrel Therapy: 2022 Update. Clin. Pharmacol. Ther..

[116] Damkier P., Lassen D., Christensen M.M.H., Madsen K.G., Hellfritzsch M., Pottegård A. (2019). Interaction between Warfarin and Cannabis. Basic Clin. Pharmacol. Toxicol..

[117] Ho J.J.Y., Goh C., Leong C.S.A., Ng K.Y., Bakhtiar A. (2024). Evaluation of Potential Drug–Drug Interactions with Medical Cannabis. Clin. Transl. Sci..

[118] Yeung C.H.T., Beers J.L., Jackson K.D., Edginton A.N. (2023). Verifying in Vitro-determined Enzyme Contributions to Cannabidiol Clearance for Exposure Predictions in Human through Physiologically-based Pharmacokinetic Modeling. CPT Pharmacomet. Syst. Pharmacol..

[119] Borges S., Desta Z., Li L., Skaar T., Ward B., Nguyen A., Jin Y., Storniolo A., Nikoloff D., Wu L. (2006). Quantitative Effect of CYP2D6 Genotype and Inhibitors on Tamoxifen Metabolism: Implication for Optimization of Breast Cancer Treatment. Clin. Pharmacol. Ther..

[120] U.S. Food and Drug Administration ONFI (Clobazam) Tablets, for Oral Use; ONFI (Clobazam) Oral Suspension: Prescribing Information. https://www.accessdata.fda.gov/drugsatfda_docs/label/2024/202067s008%2C203993s010lbl.pdf.

[121] Van Der Wouden C.H., Böhringer S., Cecchin E., Cheung K.-C., Dávila-Fajardo C.L., Deneer V.H.M., Dolžan V., Ingelman-Sundberg M., Jönsson S., Karlsson M.O. (2020). Generating Evidence for Precision Medicine: Considerations Made by the Ubiquitous Pharmacogenomics Consortium When Designing and Operationalizing the PREPARE Study. Pharmacogenet. Genom..

[122] Cicali E.J., Elchynski A.L., Cook K.J., Houder J.T., Thomas C.D., Smith D.M., Elsey A., Johnson J.A., Cavallari L.H., Wiisanen K. (2021). How to Integrate CYP2D6 Phenoconversion into Clinical Pharmacogenetics: A Tutorial. Clin. Pharmacol. Ther..

[123] Saeed L.H., Mayet A.Y. (2013). Genotype-Phenotype Analysis of CYP2C19 in Healthy Saudi Individuals and Its Potential Clinical Implication in Drug Therapy. Int. J. Med. Sci..

[124] Zhou Y., Nevosadová L., Eliasson E., Lauschke V.M. (2023). Global Distribution of Functionally Important CYP2C9 Alleles and Their Inferred Metabolic Consequences. Hum. Genom..

[125] Zhou Y., Lauschke V.M. (2022). Population Pharmacogenomics: An Update on Ethnogeographic Differences and Opportunities for Precision Public Health. Hum. Genet..

[126] Rajman I., Knapp L., Morgan T., Masimirembwa C. (2017). African Genetic Diversity: Implications for Cytochrome P450-Mediated Drug Metabolism and Drug Development. eBioMedicine.

[127] Alzahrani A.M., Ragia G., Hanieh H., Manolopoulos V.G. (2013). Genotyping of *CYP2C9* and *VKORC1* in the Arabic Population of Al-Ahsa, Saudi Arabia. Biomed. Res. Int..

[128] Blond B.N., Cardoza C., Mattson R.H. (2026). Cannabidiol Inhibits Phenytoin Clearance and Can Result in Clinical Changes: Two Cases. Seizure.

[129] Darwich A.S., Polasek T.M., Aronson J.K., Ogungbenro K., Wright D.F.B., Achour B., Reny J.-L., Daali Y., Eiermann B., Cook J. (2021). Model-Informed Precision Dosing: Background, Requirements, Validation, Implementation, and Forward Trajectory of Individualizing Drug Therapy. Annu. Rev. Pharmacol. Toxicol..

