# Supplementary material for: Convergent Modifiers of Exposure Variability in Approved Cannabinoid Medicines: Pharmacogenomics, Drug–Drug Interactions, and Parent–Metabolite Divergence
Source: Pharmaceutics. 2026 Oct 12;18(10):1293. doi: 10.3390/pharmaceutics18101293 (PMC13708062; doi:10.3390/pharmaceutics18101293)
Supplement: Supplementary file 1 [file pharmaceutics-18-01293-s001.zip › pharmaceutics-4566934-supplementary.pdf]

Convergent Modifiers of Exposure Variability in Approved Cannabinoid Medicines: Pharmacogenomics, Drug–Drug Interactions, and Parent–Metabolite Divergence

Amina M. Bagher

Table S1. Search documentation.

Coverage end date: 13 August 2026.

S1.1. Sources and role

Source	Role	Coverage end	Limits applied
PubMed/MEDLINE	Primary retrieval	13 August 2026	English language; no lower date limit
Scopus	Primary retrieval	13 August 2026	English language; no lower date limit
Web of Science	Primary retrieval	13 August 2026	English language; no lower date limit
Google Scholar	Supplementary check for records not retrieved by the three indexed databases	13 August 2026	English language
Regulatory sources	Primary documents, consulted directly (S1.3); re-checked against the live agency or manufacturer record before submission	—	—

For all four databases, including Google Scholar, the dates on which individual searches were run, the numbers of records retrieved, screened and excluded, and any stopping rule were not retained and are therefore not reported; no stopping rule was prespecified. Limits beyond those stated were not recorded. Accordingly, the original search is not fully reproducible, and no study-flow diagram can be constructed.

S1.2. Representative search string and database adaptations

("cannabidiol" OR "Epidiolex" OR "nabiximols" OR "Sativex" OR "dronabinol" OR "nabilone") AND ("CYP2C19" OR "CYP3A4" OR "CYP2C9" OR "UGT" OR pharmacogenetic* OR "drug interaction" OR "pharmacovigilance" OR "adverse event" OR "precision dosing")

Database adaptations. The strings below express the representative string above in the syntax of each database. They are provided so that a reader can run an equivalent search; they are not a record of the strings originally executed, which were not retained. Running them now will return a larger set than the original search, because the literature has grown since the coverage end date of 13 August 2026.

PubMed/MEDLINE: (("cannabidiol"[tiab] OR "Epidiolex"[tiab] OR "nabiximols"[tiab] OR "Sativex"[tiab] OR "dronabinol"[tiab] OR "nabilone"[tiab]) AND ("CYP2C19"[tiab] OR "CYP3A4"[tiab] OR "CYP2C9"[tiab] OR "UGT"[tiab] OR pharmacogenetic*[tiab] OR "drug interaction"[tiab] OR "pharmacovigilance"[tiab] OR "adverse event"[tiab] OR "precision dosing"[tiab])) AND English[lang]

Scopus: TITLE-ABS-KEY(("cannabidiol" OR "Epidiolex" OR "nabiximols" OR "Sativex" OR "dronabinol" OR "nabilone") AND ("CYP2C19" OR "CYP3A4" OR "CYP2C9" OR "UGT" OR pharmacogenetic* OR "drug interaction" OR "pharmacovigilance" OR "adverse event" OR "precision dosing")) AND LANGUAGE(english)

Web of Science: TS=(("cannabidiol" OR "Epidiolex" OR "nabiximols" OR "Sativex" OR "dronabinol" OR "nabilone") AND ("CYP2C19" OR "CYP3A4" OR "CYP2C9" OR "UGT" OR pharmacogenetic* OR "drug interaction" OR "pharmacovigilance" OR "adverse event" OR "precision dosing")) AND LA=(English)

No adaptation is given for Google Scholar. Its interface does not support field-restricted Boolean queries of this form, the terms were entered as free text, and the queries actually used were not recorded; a reconstruction would therefore be a fabrication rather than a reproduction.

Truncation. The wildcard in pharmacogenetic* must be left unquoted. Quoting a term disables truncation in all three databases, and square brackets in PubMed are field-tag delimiters rather than grouping operators; grouping requires parentheses.

S1.3. Regulatory sources

Document	Product / role	Revision date [ref.]
FDA Prescribing Information, EPIDIOLEX	Cannabidiol oral solution	05/2026 [5]
FDA Prescribing Information, MARINOL	Dronabinol capsules	03/2026 [29]
FDA Prescribing Information, CESAMET	Nabilone capsules	04/2022 [6]
UK Summary of Product Characteristics, Sativex	Nabiximols oromucosal spray	04/2026 [3]
FDA Prescribing Information, ONFI	Clobazam, interacting co-medication	03/2024 [120]
FDA Prescribing Information, PROGRAF	Tacrolimus, interacting co-medication	08/2023 [35]
Superseded 2025 EPIDIOLEX label	Retained solely to document a change in the regulatory characterization of 7-COOH-CBD	06/2025 [51]

Dates are the revision date printed on the document consulted. CESAMET prints "Rev. 11/2021"; the version cited is the labeling approved under NDA 018677/S-017 on 28 April 2022, and that approval date is given here. Each document was rechecked against the live agency or manufacturer record before submission.

S1.4. Relationships covered by targeted update searches before the original submission

Relationship	Negative claim it bounds
CYP2C19 and cannabidiol	No approved-product genotype-by-comedication study identified
CYP2C9 and dronabinol or oral Δ^9-THC	No genotype-stratified interaction study in the approved capsule
CYP3A5, cannabidiol, and tacrolimus	No completed genotype-stratified analysis beyond the interim programme
UGT1A9 or UGT2B7 and cannabidiol	No study relating UGT genotype to co-substrate exposure under cannabinoid inhibition
ABCB1 or P-glycoprotein and cannabinoid exposure	No study relating ABCB1 genotype to cannabinoid exposure in an approved product was identified
Nabiximols pharmacogenomics	No genotype-stratified pharmacokinetic study in the approved product

Negative claims in the review are bounded by these searches, which were run before the original submission. Dates run and counts screened were not recorded. A separate revision-stage verification search of these same six relationships was run on 1 October 2026 and is reported in S1.5.

S1.5. Revision-stage verification search (run 1 October 2026)

Relationship	PubMed query as run	Records identified / assessed	Outcome
CYP2C19 and cannabidiol	("cannabidiol"[tiab] OR "Epidiolex"[tiab]) AND "CYP2C19"[tiab] AND (genotype[tiab] OR genotypes[tiab] OR polymorphism[tiab] OR polymorphisms[tiab] OR metabolizer[tiab] OR metabolizers[tiab] OR phenotype[tiab] OR phenoconversion[tiab]) AND English[lang]	11 / 11	Claim holds. No approved-product genotype-by-comedication study identified. One case report of CYP2C19 phenoconversion by CBD added to Section 5.6 as hypothesis-generating.
CYP2C9 and dronabinol or oral Δ⁹-THC	("dronabinol"[tiab] OR "Marinol"[tiab] OR "oral THC"[tiab] OR "oral tetrahydrocannabinol"[tiab] OR "nabilone"[tiab]) AND "CYP2C9"[tiab] AND English[lang]	2 / 2	Claim holds. No genotype-stratified interaction study in the approved capsule identified.
CYP3A5, cannabidiol and tacrolimus	"cannabidiol"[tiab] AND "tacrolimus"[tiab] AND English[lang]	12 / 12	Claim holds. No completed genotype-stratified analysis beyond the interim programme identified.
UGT1A9 or UGT2B7 and cannabidiol	"cannabidiol"[tiab] AND ("UGT1A9"[tiab] OR "UGT2B7"[tiab] OR "glucuronidation"[tiab] OR "glucuronosyltransferase"[tiab]) AND (genotype[tiab] OR genotypes[tiab] OR polymorphism[tiab] OR polymorphisms[tiab] OR pharmacogenetic[tiab] OR pharmacogenomic[tiab]) AND English[lang]	0 / 0	Claim holds. No record retrieved.
ABCB1 or P-glycoprotein and cannabinoid exposure	("ABCB1"[tiab] OR "P-glycoprotein"[tiab] OR "MDR1"[tiab]) AND ("cannabinoid"[tiab] OR "cannabinoids"[tiab] OR "cannabidiol"[tiab] OR "tetrahydrocannabinol"[tiab] OR "cannabis"[tiab]) AND (genotype[tiab] OR genotypes[tiab] OR polymorphism[tiab] OR polymorphisms[tiab] OR pharmacokinetics[tiab] OR "plasma concentration"[tiab] OR "blood levels"[tiab]) AND English[lang]	20 / 20	Claim holds for approved products. Two human studies relating ABCB1 genotype to cannabinoid exposure were identified, both in unregulated cannabis and not directly comparable in analyte, exposure condition or variant; added to Section 5.5 as indirect evidence. Two further genotype studies address analgesic response or dependence rather than exposure and are also now cited in Section 5.5.
Nabiximols pharmacogenomics	("nabiximols"[tiab] OR "Sativex"[tiab]) AND (pharmacogenetic[tiab] OR pharmacogenetics[tiab] OR pharmacogenomic[tiab] OR pharmacogenomics[tiab] OR genotype[tiab] OR genotypes[tiab] OR polymorphism[tiab] OR polymorphisms[tiab] OR "CYP2C19"[tiab] OR "CYP2C9"[tiab] OR "CYP3A4"[tiab] OR pharmacokinetics[tiab]) AND English[lang]	21 / 21	Claim holds. No genotype-stratified pharmacokinetic study in the approved product identified.

Purpose and protocol. This search was confirmatory and is reported separately from the original search; it is not the search from which the review was written. The protocol was fixed before any record was screened: six relationship-specific PubMed queries, the eligibility criteria of Section 2, and the rule that a claim would be rewritten only where a qualifying study was identified. PubMed/MEDLINE only; English language; no lower date limit. Across the six queries 66 records were identified, all unique, and all 66 were assessed: 64 on title and abstract, and two (PMID 31550100 and PMID 33211456) from the indexed record alone, these being chapters of Medical Genetics Summaries (NCBI Bookshelf) that are indexed without an abstract. Neither is a primary study; the dronabinol chapter (PMID 33211456) is cited in this review as reference [18]. The six approved-product evidence-gap statements were retained; indirect evidence prompted qualification of two broader statements, in Sections 5.5 and 5.6.

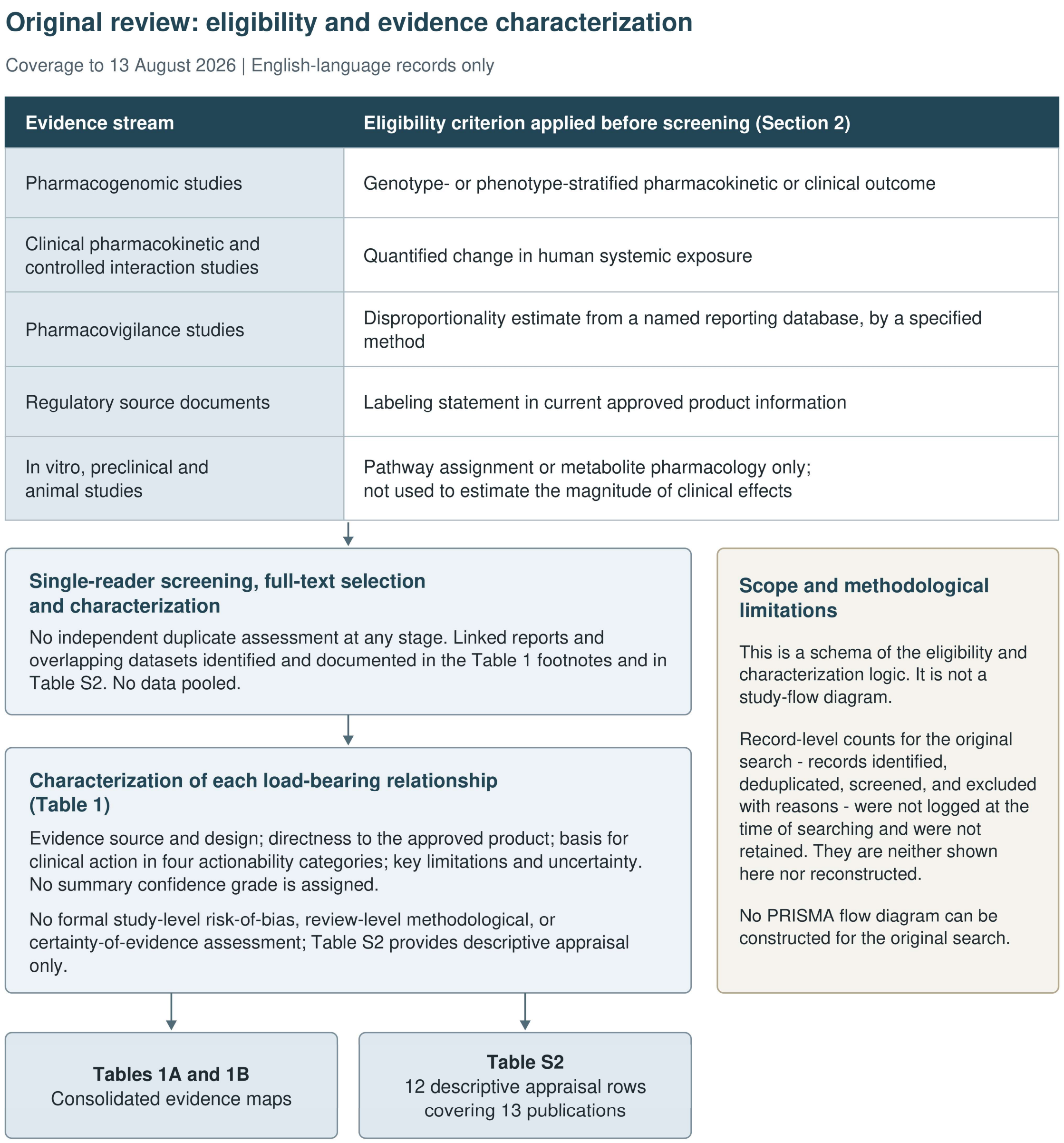

Figure S1. Evidence-selection and characterization schema for the narrative integrative review.

The figure summarizes the eligibility, single-reader selection, and evidence-characterization procedures described in Section 2. Original record-level identification, screening, and exclusion counts were not retained; this schema does not reconstruct a study-flow diagram, and no PRISMA flow diagram can be constructed for the original search. Table S2 contains 12 descriptive appraisal rows covering 13 publications and does not constitute a validated risk-of-bias or certainty-of-evidence assessment. The targeted revision-stage verification search of 1 October 2026 is reported separately in S1.5.

Table S2. Structured descriptive critical appraisal of pivotal studies.

Source (ref.)	Design [status]	Population and n	Formulation and dosing	Effect size and precision	Replication	Dataset overlap	Inference supported	Key limitations
Sachse-Seeboth et al., 2009 [28]	Genotype-stratified single-dose pharmacokinetic study in healthy volunteers; nonblinded, as stated by the authors [V]	43 healthy volunteers (16 female, 27 male), selected from a larger pool to enrich variant genotypes; CYP2C9 *1/*1 n = 19, *1/*2 n = 6, *2/*2 n = 4, *1/*3 n = 9, *2/*3 n = 1, *3/*3 n = 4 [V]	Single oral 15 mg dose of Δ^9-THC; marketed formulation not specified [V]	Median THC AUC approximately threefold higher and THC-COOH approximately 70% lower in *3/*3 versus *1/*1; median clearance 932, 473 and 365 L/h across *1/*1, *1/*3, *3/*3; Jonckheere-Terpstra trend test [V]	None identified [V]	None	Reduced-function CYP2C9 raises oral THC exposure	Single unreplicated study; the principal contrast rests on four *3/*3 carriers against nineteen *1/*1; genotype-enriched sampling, so allele frequencies are not population estimates; single 15 mg dose of oral Δ^9-THC of unspecified formulation; applicability to the approved dronabinol capsule is unconfirmed. The authors attribute the uncertain sedation association to limited sample size and nonblinded design; blinding does not bear on the plasma concentration measurements [V]
Morrison et al., 2019 [31]	Phase 1, open-label, fixed-sequence drug–drug interaction trial in healthy volunteers, conducted 4 November 2015 to 18 November 2016 at a single early-development unit [V]	78 healthy subjects enrolled across all arms, of whom 1 withdrew before treatment; the clobazam victim arm comprised 12 subjects and the reciprocal cannabidiol victim arm 15 [V]	Cannabidiol oral solution 750 mg twice daily with clobazam 5 mg twice daily, both to steady state [V]	N-desmethylclobazam Cmax and AUC increased 3.4-fold; parent clobazam 1.2-fold; treatment ratios reported with 90% confidence intervals [V]	Directionally supported by Geffrey 2015 in a different population [V]	Labeling statement [5] derives from this programme; counted as one source	CBD raises N-desmethylclobazam exposure via CYP2C19 inhibition	Open-label; healthy volunteers rather than patients; the clobazam arm rests on 12 subjects; sponsor-conducted, and the labeling statement derives from this programme rather than from independent replication [V]
VanLandingham et al., 2020 [32]	Phase 2, randomized, double-blind, placebo-controlled drug–drug interaction trial [V]	Twenty adult patients with uncontrolled epilepsy on stable clobazam, randomized 4:1 to cannabidiol (n = 16) or placebo (n = 4); the pharmacokinetic analysis sets comprised 10 cannabidiol-treated and 3 placebo-treated patients, seven being excluded for misdosing, withdrawal of consent, treatment-emergent adverse events, or clobazam dose reduction [V]	Plant-derived highly purified cannabidiol oral solution, 20 mg/kg/day, 21-day maintenance dosing [V]	Within the cannabidiol group (n = 10), N-desmethylclobazam geometric mean ratio, day 33 to day 1: AUC 2.6 (90% CI 2.0–3.6); maximum concentration 2.2 (90% CI 1.4–3.5). Clobazam itself unchanged: AUC 1.1 (90% CI 0.9–1.2); maximum concentration 1.0 (90% CI 0.8–1.2). Placebo group (n = 3) showed no effect: N-desmethylclobazam AUC 1.0 (90% CI 0.8–1.3) [V]	Independent controlled estimate of the effect reported in the phase 1 trial [31]	Independent of the phase 1 healthy-volunteer programme [31]	Confirms and quantifies the CBD effect on the active metabolite N-desmethylclobazam while parent clobazam exposure is unaffected	Ten patients contributed cannabidiol-group pharmacokinetic data and three the placebo group; the headline ratio is a within-group day 33 to day 1 comparison rather than a cannabidiol-versus-placebo contrast, although the placebo group showed no effect on either analyte; interpatient variability was moderate to high; the trial was sponsor-conducted
Geffrey et al., 2015 [30]	Observational cohort within an expanded-	13 children with refractory epilepsy taking clobazam [V]	Cannabidiol as adjunct; clobazam doses reduced	Mean N-desmethylclobazam increase 500 ± 300% (95% CI +90 to +610% at 4 weeks);	Directionally consistent with	None	Magnitude of the clobazam interaction in children under	Uncontrolled; small; concomitant antiepileptic drugs; clobazam dose reduction in most

Source (ref.)	Design [status]	Population and n	Formulation and dosing	Effect size and precision	Replication	Dataset overlap	Inference supported	Key limitations
	access IND; not a controlled PK trial [V]		in 10 of 13 during follow-up [V]	clobazam 60 ± 80% (95% CI -2 to +91%) [V]	Morrison 2019 [V]		clinical dose adjustment	subjects truncates the observable rise; wide confidence interval
So et al., 2025 [33]	Prespecified interim analysis of an open-label, three-period, fixed-sequence crossover phase I trial [V]	12 participants completed all periods [V]	Tacrolimus 5 mg single oral dose; cannabidiol (Epidiolex) titrated to 5 mg/kg twice daily for 14 days [V]	Tacrolimus Cmax 4.2-fold (40.2 ± 13.5 vs 9.85 ± 4.63 ng/mL, P < 0.0001); AUC 3.1-fold (P < 0.0001); half-life unchanged [V]	None; interim analysis of a single trial [V]	Same parent trial (NCT05490511) as Etkins 2026 [40]; not independent evidence [V]	CBD raises tacrolimus exposure	Interim; 12 completers; single centre; healthy volunteers, not transplant recipients; mechanism (CYP3A4 vs CYP3A5, hepatic vs intestinal vs transporter) not resolved by the authors
Wray et al., 2023 [37]	Single-centre, open-label, fixed-sequence phase 1 drug–drug interaction study in healthy adults [V]	Sixteen healthy adult volunteers contributed pharmacokinetic data in both periods [V]	Everolimus 5 mg single oral dose on day 1; after a 7-day washout, cannabidiol oral solution (100 mg/mL) 12.5 mg/kg twice daily on days 9–17, with a second single everolimus 5 mg dose on day 13; doses taken after a standardized meal [V]	Geometric mean ratios, everolimus with steady-state cannabidiol versus alone: maximum concentration 2.50 (90% CI 2.12–2.94); AUC to the last measurable concentration 2.55 (90% CI 2.23–2.90); AUC extrapolated to infinity 2.45 (90% CI 2.15–2.80); apparent oral clearance 23.2 to 9.45 L/h; mean terminal half-life 40.0 (SD 5.10) h alone and 33.2 (SD 4.17) h with cannabidiol, not prolonged; median tmax delayed 0.75 h [V]	None identified	None	Steady-state cannabidiol raises everolimus systemic exposure; the authors advise everolimus blood concentration monitoring with appropriate dose reduction on coadministration	Healthy volunteers rather than patients with tuberous sclerosis complex; single centre; open-label, fixed-sequence design without a randomized comparator; a single 5 mg everolimus dose; 16 participants; sponsor-conducted. The lower apparent oral clearance and absence of half-life prolongation are compatible with an absorption-related mechanism but do not establish it
Leino et al., 2019 [34]	Case report; indexed as Case Reports [V]	One participant in a CBD epilepsy trial also receiving tacrolimus [V]	Pharmaceutical cannabidiol 20–25 mg/kg/day (reported as 2,000–2,900 mg/day), within the approved dosage range: maximum recommended 20 mg/kg/day for Lennox–Gastaut and Dravet syndromes and recommended maintenance 25 mg/kg/day for tuberous sclerosis complex [V]	Approximately threefold rise in dose-normalized tacrolimus concentration; no dispersion statistics [V]	Not applicable	Independent of the So/Etkins programme	Corroborates direction of the CBD-tacrolimus interaction	Provenance, indication and dose applicability are separate here. Provenance is a pharmaceutical cannabidiol product at a dose within the approved range, so the report is direct approved-product evidence; the treated indication was epilepsy within a trial, not routine labeled use. The design is a single uncontrolled case with no dispersion statistics, so it corroborates direction only and is not controlled evidence and supports no magnitude estimate
Etkins et al., 2026 [40]	Genotype-stratified secondary analysis of the same open-label, fixed-sequence, single-centre phase 1 trial [V]	33 participants received at least one CBD dose; pharmacokinetic parameters determined in 30 (2 withdrawals, 1 incomplete sampling); CYP2C19	Cannabidiol (Epidiolex) 5 mg/kg single dose, then 2.5 mg/kg for 3 days and 5 mg/kg twice daily for 11 days; standardized high-fat meal [V]	Parent-to-metabolite ratio lowest in CYP2C19 intermediate metabolizers: single dose 0.69 vs 1.54 (NM) vs 3.08 (RM), p = 0.0143; steady state 1.38 vs 2.02 vs 4.20, p = 0.0033. Diarrhea 7 of 18	Direction reproduced in an external validation cohort from the FDA Epidiolex	Same parent trial (NCT05490511) as So 2025 [33]; overlapping subsets of differing size [V]	CYP2C19 status associates with CBD exposure and parent-to-metabolite ratio	Secondary analysis; small genotype subgroups; phase 1 metabolism only; direction of the CYP2C19 effect is paradoxical and unexplained by the authors; phenotype grouping raises type

Source (ref.)	Design [status]	Population and n	Formulation and dosing	Effect size and precision	Replication	Dataset overlap	Inference supported	Key limitations
		IM n = 7, NM n = 15, RM n = 6 [V]		(39%) vs 1 of 15 (7%), p = 0.0463 [V]	clinical pharmacology review [V]			I and type II error risk, as the authors state
Bansal et al., 2023 [44]	Randomized crossover controlled interaction study in healthy adults [V]	18 healthy adults [V]	Brownie containing CBD-dominant cannabis extract (640 mg CBD + 20 mg Δ⁹-THC), Δ⁹-THC-dominant extract (20 mg), or placebo, with a CYP probe cocktail [V]	Δ⁹-THC AUC increased 161% with the CBD-dominant extract; probe AUC increases: omeprazole 207%, losartan 77%, midazolam 56%, caffeine 39%; no inhibition by the THC-only extract [V]	No independent clinical replication identified. The authors' physiologically based pharmacokinetic model predicted the observed exposures within 26%, which is model prediction performance rather than independent clinical replication [V]	None	CBD raises oral Δ⁹-THC exposure, consistent with CYP2C9 inhibition	Cannabis extract in a food matrix, not an approved product; CBD dose far above nabiximols exposure; relevance to nabiximols untested
Cortopassi et al., 2020 [41]; Grayson et al., 2018 [42]	Case reports; both indexed as Case Reports [V]	One patient each; Cortopassi a 46-year-old male on warfarin for deep venous thrombosis [V]	FDA-approved cannabidiol (Epidiolex), titrated [V]	Approximately 20% warfarin dose reduction required (Cortopassi); the earlier case reached INR 6.86 and required a 30% weekly dose reduction [V]	Two independent reports, not controlled replication [V]	Independent of each other	Association between CBD initiation and raised INR	Case reports; no controlled exposure data; mechanism unassigned; confounding by comedication and indication not excluded
Rubin et al., 1977 [88]	Open human disposition study with ¹⁴C-radiolabel; oral and intravenous administration [V]	Eight male volunteers aged 23–43 years, single centre; the terminal half-life of total radioactivity was derived from five of them [V]	Oral 2 mg ¹⁴C-nabilone (50 μCi) as a polyvinylpyrrolidone coprecipitate after a 10-hour fast; intravenous 0.5 mg (12 μCi); not the marketed capsule [V]	Parent plasma half-life approximately 2 h; total radioactivity half-life 20.6 h (SE ± 1.3; range 17–25 h, n = 5); faecal elimination approximately 65% and urinary approximately 20% of dose [V]	None identified	None	Nabilone metabolites persist far longer than parent; the authors conclude effects may be exerted at least in part by one or more active metabolites	Eight subjects; 1977 methodology; an experimental PVP coprecipitate rather than the approved capsule; metabolite activity inferred from the mismatch between short parent half-life and long duration of action, not measured. The study did not identify the metabolites recovered [V]. Nabilone's parent-drug activity is established, but the contribution of potentially active metabolites remains unresolved
Calapai et al., 2023 [11]	Descriptive analysis followed by disproportionality analysis in VigiBase, using the reporting odds ratio (ROR) and information component (IC); case set defined a priori as	12,702 CBD-related ICSRs in VigiBase to 15 May 2023, of which 5,466 (43.0%) serious; analysis set 820 ICSRs (6.5%) reporting one or more of the five terms;	Epidiolex named in 753 of 820 ICSRs (91.8%); unspecified cannabidiol 47 (5.7%); CBD oil 15 (1.8%); doses not reported [V]	ROR (95% CI): weight decreased 5.19 (4.54–5.70), n = 456; hypophagia 3.68 (3.22–5.27), n = 30; insomnia 1.60 (1.40–1.83), n = 221; dizziness 0.23 (0.20–0.27), n = 118; palpitations 0.12 (0.10–0.19), n =	None identified for these terms. A separate EudraVigilance analysis from the same group	Not assessed	Signals used only to prioritize which convergences to investigate; no metabolic pathway, genotype, or analyte is linked to any of the five terms	Disproportionality is not a measure of exposure, incidence, or causation; the five terms were chosen a priori for a single mechanism, so the analysis is not a screen of CBD safety; 92.0% of reports from one

Source (ref.)	Design [status]	Population and n	Formulation and dosing	Effect size and precision	Replication	Dataset overlap	Inference supported	Key limitations
	five MedDRA preferred terms selected for plausibility at the 5-HT1A receptor; comparator all other VigiBase reports; reported following READUS-PV [V]	754 (92.0%) from the USA; 463 (56.5%) reported by consumers; age not reported in 685 (83.5%) [V]		16. IC025 positive for the first three (2.2, 1.3, 0.5) [V]	(Ammendolia 2023 [10]) is descriptive and reports no disproportionality estimate			country and 56.5% from consumers; age missing in 83.5%; reporting, indication, and notoriety bias not excluded. The hypophagia interval is asymmetric about its point estimate and is reproduced as printed

Selection for appraisal: studies supplying a quantitative value reproduced in the main text or Table 1, or underpinning one of the framework hypotheses. This is a structured descriptive appraisal; no validated risk-of-bias, review-appraisal, or certainty instrument was applied, and it does not substitute for one.

Domain coverage. The columns record the constraints that bear on an exposure estimate, and they map onto domains common to established appraisal frameworks: Design [status] and Formulation and dosing cover risk arising from the study design and from departure of the product or dose from the approved medicine; Population and n covers selection and applicability; Effect size and precision covers imprecision; Replication and Dataset overlap cover independence of the evidence base and the risk of counting one dataset twice; and Key limitations records residual threats, including open-label conduct, interim analysis, and confounding by comedication. Reporting bias was not assessed at study level and no small-study or funnel-plot analysis was undertaken, because no quantitative pooling was performed. The mapping is given to show which domains are covered; it does not claim equivalence with a validated instrument.

Verification status: every entry is marked [V], verified against the indexed record or full text of the primary source. Entries were checked against PubMed and PubMed Central on 24 September 2026; the Calapai 2023 entry was checked against the full text on 27 September 2026; the VanLandingham 2020 and Wray 2023 entries were checked against the full text on 30 September 2026. These checks verified already included sources and did not extend the literature-coverage end date of 13 August 2026.

Sources verified via PubMed and PubMed Central (retrieved 24 September 2026). DOIs: 10.1038/clpt.2008.213; 10.1002/cpdd.665; 10.1111/epi.13060; 10.1002/cpt.3504; 10.1111/ajt.15398; 10.1111/cts.70455; 10.1002/cpt.2973; 10.1093/ajhp/zxaa268; 10.1016/j.ebcr.2017.10.001; 10.1002/cpt197722185; 10.3390/ph16101420 (full text); 10.1002/jcph.1634 (full text); 10.1002/cpdd.1262 (full text). Trial registry record NCT05490511 retrieved from ClinicalTrials.gov.
